# Deciphering genetic causality between plasma BDNF and 91 circulating inflammatory proteins through bidirectional mendelian randomization

**DOI:** 10.1038/s41598-025-95546-1

**Published:** 2025-03-25

**Authors:** Yesheng Sun, Xizi Shi, Melanie Ohm, Martin Korte, Marta Zagrebelsky

**Affiliations:** 1 Division of Cellular Neurobiology, Zoological Institute, TU Braunschweig, Braunschweig, Germany; 2 Research Group Neuroinflammation and Neurodegeneration, Helmholtz Centre for Infection Research, AG NIND, Braunschweig, Germany

**Keywords:** BDNF, Neurotrophin family, Inflammatory cytokines, Aging, Mendelian randomization, Immunology, Cytokines, Inflammation, Neuroscience, Diseases of the nervous system, Molecular neuroscience, Neuroimmunology, Neurotrophic factors

## Abstract

Prior studies reported an association between the levels of brain-derived neurotrophic factor (BDNF) circulating in the bloodstream and those of different inflammatory factors. However, their causal relationship remains unclear. Here, we performed a Mendelian randomization (MR) study to investigate the causal relationships between plasma BDNF levels and 91 circulating inflammatory proteins to shed light on the possible role of BDNF in the pathogenesis and progression of inflammation-related neurological diseases in order to distinguish correlation from possible causal effects. Data for plasma BDNF levels were derived from a genome-wide association study (GWAS) encompassing 3,301 European participants. Genetic association estimates for 91 inflammation proteins were extracted from a GWAS meta-analysis that enrolled 14,824 European participants. The primary MR analysis employed the inverse variance weighted (IVW) method and was corroborated by additional methods including MR-Egger, weighted median, weighted mode, and simple mode. Analyses of sensitivity were performed by evaluating the heterogeneity, horizontal pleiotropy, and robustness of the results. Genetic evidence indicated that elevated plasma BDNF levels possibly contribute to decreased concentrations of 13 inflammation proteins (OR: 0.951–0.977), including beta-nerve growth factor (Beta-NGF), caspase 8 (CASP-8), interleukin-15 receptor subunit alpha (IL-15RA), interleukin-17 A (IL-17 A), interleukin-17 C (IL-17 C), interleukin-2 (IL-2), interleukin-20 (IL-20), interleukin-20 receptor subunit alpha (IL-20RA), interleukin-24 (IL-24), interleukin-33 (IL-33), leukemia inhibitory factor (LIF), neurturin (NRTN), as well as neurotrophin-3 (NT-3). The associations between BDNF and IL-33 remained statistically significant after FDR correction (FDR > 0.05). Furthermore, reverse MR analysis showed that C-C motif chemokine 23 (CCL23), CUB domain-containing protein 1 (CDCP1), and NRTN is suggestive for a positive causal effect on BDNF plasma levels (OR: 1.240–1.422). Moreover, 5 proteins are likely to be associated with lower plasma levels of BDNF (OR: 0.742–0.971), including adenosine deaminase (ADA), cystatin D (CST5), interleukin-13 (IL-13), interleukin-17 A (IL-17 A), and vascular endothelial growth factor A (VEGF-A). Genetically determined plasma BDNF levels influence IL-33 and are possibly associated with 12 circulating inflammatory proteins. The data suggest that 8 inflammatory proteins exhibit either negative or protective roles to BDNF levels, respectively. Of these, 5 are negatively associated with BDNF levels, while 3 play protective roles. These findings may offer new theoretical and empirical insights into the pathogenesis and progression of inflammation-related neurological diseases.

**Supplementary Information:**

The online version contains supplementary material available at 10.1038/s41598-025-95546-1.

## Introduction

BDNF, a member of the neurotrophin family, and its precursor proBDNF are widely distributed across various brain regions including the cortex and hippocampus, and play critical roles in enhancing neurogenesis^1,2^, neuroprotection^3^, neuronal survival^4^, and synaptic plasticity^5–7^ in the developing and adult central nervous system (CNS). BDNF levels in the brain decline with increasing age^8^. Moreover, low levels of BDNF lead to deficits in hippocampal and cortical neural plasticity and are associated with neurodegenerative disorders such as Alzheimer’s disease (AD)^9^, Parkinson’s disease (PD)^10^, and Huntington’s disease (HD)^11^. Similarly, BDNF levels in plasma decreased significantly with increasing age^12,13^, and low plasma BDNF levels are a correlate of the risk of developing AD^14^, mild cognitive impairment (MCI)^14^, as well as suicidal behaviour in major depression^15^. Central BDNF levels are known to be positively correlated with peripheral BDNF levels, and peripheral BDNF levels reflect central BDNF levels^16–18^. Chronic inflammation is well known to be associated with a broad spectrum of neurodegenerative diseases^19,20^. Sustained, chronic neuroinflammatory processes contribute to the onset and progression of neurodegenerative pathologies and other neurological conditions^21,22^. However, the potential effect of BDNF in modulating the levels of inflammation remains controversial.

Exogenous BDNF administration depressed microglial activation by lipopolysaccharide (LPS) injection in the substantia nigra in aged mice^23^. BDNF delivered to the CNS via the transformed bone marrow stem cells significantly reduced inflammatory infiltrating cells in the brain and spinal cord^24^. Moreover, pro-inflammatory cytokines (TNF-α) levels were significantly downregulated while anti-inflammatory cytokines (IL-10) levels were upregulated in both intranasal and intracerebral administration of BDNF in ischemic stroke mice^25,26^. In addition, Han et al. showed that BDNF overexpression in the hippocampus suppressed microglial activation and expression of TNF-α and IL-6 in streptozotocin-induced Type I diabetes^27^. In contrast to the aforementioned studies, a clinical study including 624 patients showed increased BDNF levels in patients with PD^28^, suggesting that elevated levels of BDNF may be involved in inflammation progression^29^, a discrepancy that may be attributed to the inherent complexities of observational studies and factors such as the stage of PD and the effects of pharmacological interventions. Furthermore, intrathecal injection of BDNF activated astrocytes and microglia, then increased the release of pro-inflammatory cytokines (TNF-α and IL-1β) in cystitis rat model^30^. In addition, a cohort study indicated that serum BDNF levels in AD patients were significantly lower than controls, but there was no significant difference in TNF-α and IL-1β among the groups, insinuating there was no correlation between BDNF and inflammation such as cytokines TNF-α and IL-1β^31^. These disparate conclusions might be attributable to the inherent complexity of observational studies, characterized by potential reverse causation bias and residual confounding.

Mendelian randomization (MR) is an approach that utilizes genetic variants as instruments to assess the causal effect of exposures on outcomes to enhance a causal inference^32^. Compared with traditional observational studies, causal estimates derived from MR analysis can minimize the effect of confounding factors on causal estimates because of the use of genetic variants. These are randomly assigned to the offspring during meiosis in a population, thus genetic variant allocation is not influenced by environmental or lifestyle factors^33^. Recently, two-sample MR analysis has been applied to investigate the relationship between BDNF and neurological diseases, indicating that individuals genetically predisposed to higher plasma levels of BDNF are less likely to develop AD, non-traumatic intracranial haemorrhage, epilepsy, or focal epilepsy^34–36^. However, the associations of BDNF levels in plasma with those of different inflammatory proteins have not yet been investigated using this approach. Therefore, this study aimed to evaluate the causal effect between plasma BDNF levels and inflammatory proteins by performing two-sample MR analysis using pooled data from large-scale genome-wide association studies (GWAS).

## Methods

### Study design

**Fig. 1:**
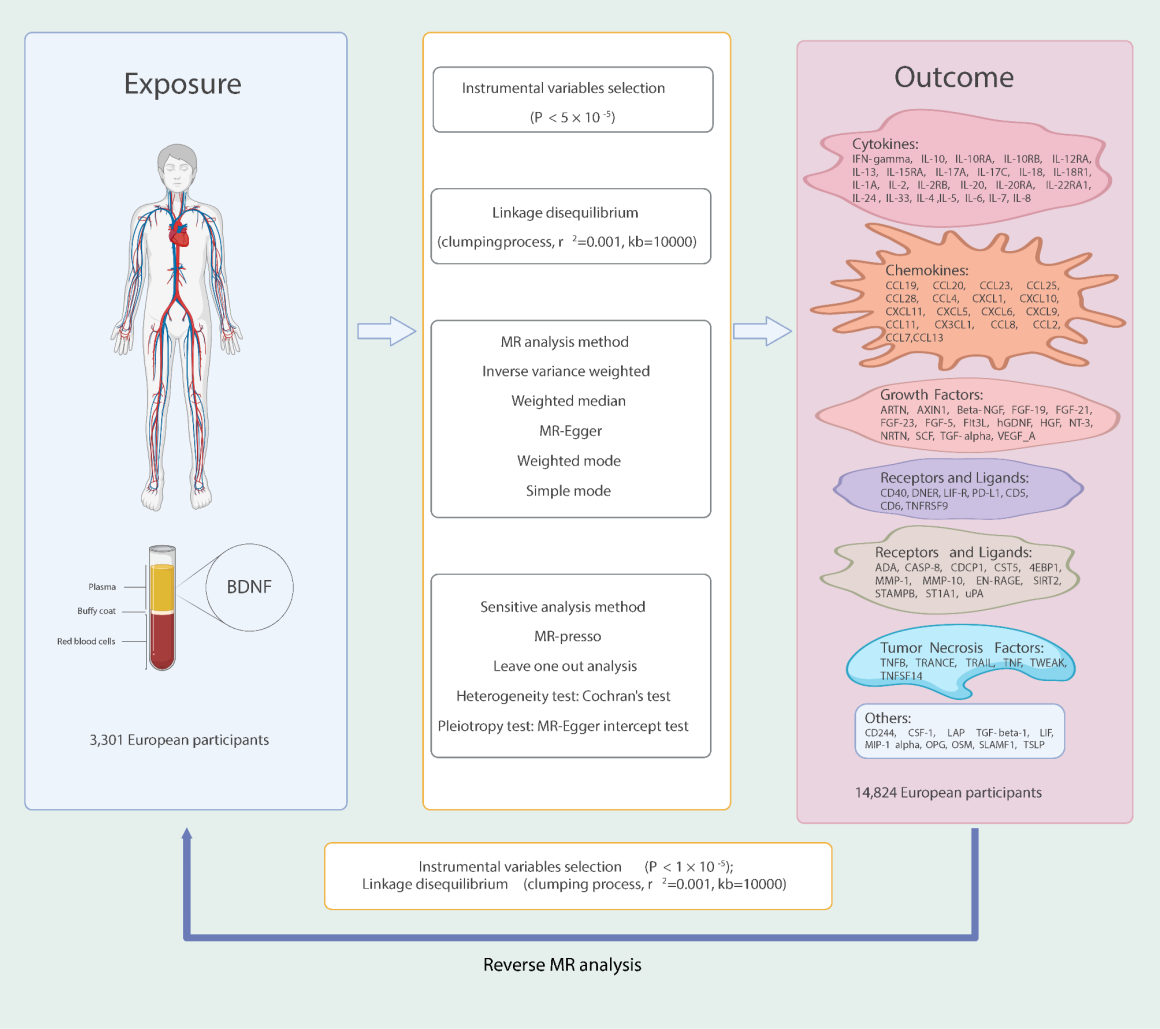
Illustration of study design and instrumental variable assumptions underlying Mendelian randomization. Figure created in BioRender (2025) https://BioRender.com/s00k123.

An overview of the study design is illustrated in Fig. 1. Briefly, for the process of MR analysis, three assumptions must be satisfied: (1) relevance assumption: instrumental variables (IVs) are strongly correlated with the exposure of interest; (2) independence assumption: IVs must not be associated with confounders of the exposure-outcome association; (3) exclusivity assumption: IVs affect the outcome variable solely through their influence on the exposure. The analytic approach follows the STROBE-MR guidelines^37^. In forward analysis, we investigated the potential causal effect of circulating plasma BDNF levels on 91 inflammatory proteins, with plasma BDNF levels as the exposure factors and 91 circulating inflammatory proteins as the outcomes. Furthermore, we investigated the potential causal effect of circulating inflammatory proteins on plasma BDNF levels and the possibility of reverse causation in the reverse MR.

## Data source

The GWAS data source for plasma BDNF levels is derived from a study including 3,301 European blood donors who were older than 18 years (accession numbers GCST90240466) and can be downloaded from the GWAS Catalog (https://www.ebi.ac.uk/gwas/)^38^. Most participants in the study were in good health, as the criteria for blood donation exclude anyone with a history of significant illnesses, such as heart attacks, strokes, cancer, HIV, or hepatitis B and C, as well as individuals who have recently been ill or had an infection. Researchers created and analyzed a detailed genetic map of the human plasma proteome using an enhanced version of the aptamer-based multiplex protein assay, which detected a total of 3,622 plasma proteins. The updated GWAS summary statistics for 91 inflammatory proteins were derived from a comprehensive genome-wide meta-analysis, in a sample of 14,824 European ancestry individuals from 11 cohorts^39^. Inflammatory proteins were generated by measuring genome-wide genetic data and plasma proteomics data with the Olink Target-96 Inflammation immunoassay panel. GWAS analysis within each cohort was performed by applying an additive genetic association model based on linear regression, and the impact of inflammatory protein was reported as changes in normalized protein levels per allele copy. Adjustments for population substructure were made using genetic principal components, with covariates such as age and sex included to control for confounders. The data are publicly available in the EBI GWAS Catalog (accession numbers GCST90274758 to GCST90274848) and can be downloaded from https://www.phpc.cam.ac.uk/ceu/proteins. All data are publicly available, and ethical approval and informed consent have been obtained in original studies.

## Instrumental variables selection criteria

To obtain a substantial number of single nucleotide polymorphisms (SNPs) and avoid excluding SNPs that may genuinely be associated with exposure, we did not use the traditional genome-wide significance threshold (*P* < 5 × 10^− 8^) owing to a small number of SNPs being included^40^. Instead, independent single nucleotide SNPs at the genome-wide significance level (*P* < 5 × 10^− 5^) were selected as IVs for BDNF, which was widely used in previous MR studies^35,36^. The SNPs strongly associated with 91 inflammatory factors (*P* < 1 × 10^− 5^) were selected as instrumental variables^41^. These SNPs were further pruned by a clumping r^2^ value of 0.001 within a 10,000 kb window to ensure the isolation of the instrumental variables^42^. The filtration criteria for instrumental variables differ between BDNF and inflammatory proteins due to variations in sample size in the GWAS datasets, the number of available SNPs, and their significance levels. To avoid inaccurate results stemming from an insufficient number of SNPs, different filtration criteria were performed. In addition, palindromic SNPs were excluded by harmonizing the exposure and outcome data. Finally, the F-value of each SNP was calculated, and SNPs with weak instrumental variables (F-value < 10) were excluded^43^.

## Sensitivity analysis

A series of sensitivity tests were performed to confirm the robustness of the findings. Cochran’s Q test was employed to identify heterogeneity among the effect estimates of each SNP^44^. MR-Egger intercept test and MR Pleiotropy Residual Sum and Outlier (MR-PRESSO) were deployed to investigate horizontal pleiotropy^45,46^. In addition, a leave-one-out analysis was conducted to evaluate whether the MR estimate was driven or biased by a single SNP. The final results were presented using scatter plots, forest plots, leave-one-out, and funnel plots.

### Statistical analysis

We performed MR analysis to investigate the causal connections between plasma BDNF levels and 91 circulating inflammatory proteins using various established MR methodologies. The inverse-variance weighted method (IVW), which is considered the most effective analysis method using reliable IVs, was used as the main analysis technique in our study^47^, supplemented by other methods such as MR-Egger, weighted median, weighted mode, and simple mode for further analysis. The IVW method combines Wald estimates for each SNP and obtains an overall estimate of the exposure’s effect on the outcome^48^. If significant heterogeneity was detected (*P* < 0.05), we applied the random effects IVW model. Otherwise, the fixed-effect IVW model was utilized. MR-PRESSO test was employed to identify and correct for outliers in the IVW linear regression analysis.

A correlation was considered statistically significant if the P-value for the IVW was less than 0.05, other methods were performed to improve the reliability of causal inference^46,49^. False discovery rate (FDR) correction instead of the Bonferroni correction was performed to correct the false positive in genome wide association analysis, as Bonferroni is a highly stringent method that tends to identify only a small number of SNPs in MR analysis (typically < = 3 or < 5% of SNPs tested). MR Results with an FDR greater than 0.05 but a p-value less than 0.05 were classified as nominally significant causal relationships, indicating potential emerging trends meriting further investigation. All analyses were performed by the R software (version 4.2.3), two-sample MR package (version 0.5.8), and MRPRESSO package (version 1.0).

## Results

### Causal associations of genetically predicted BDNF plasma levels with those of inflammatory proteins

**Fig. 2:**
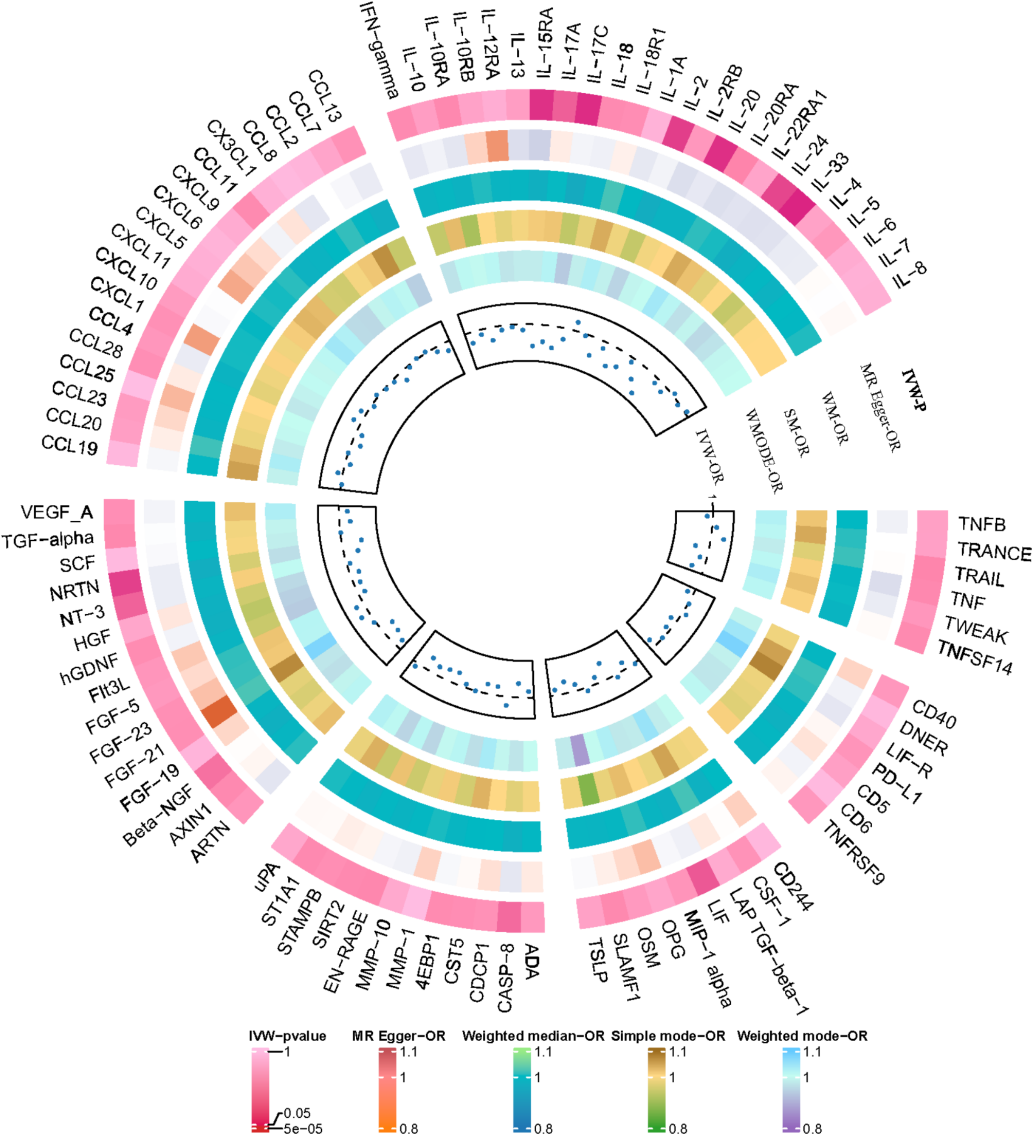
Circle diagram of plasma BDNF on 91 circulating inflammatory proteins using the five methods (IVW, MR-Egger, weighted median, weighted mode, and simple mode).

**Fig. 3:**
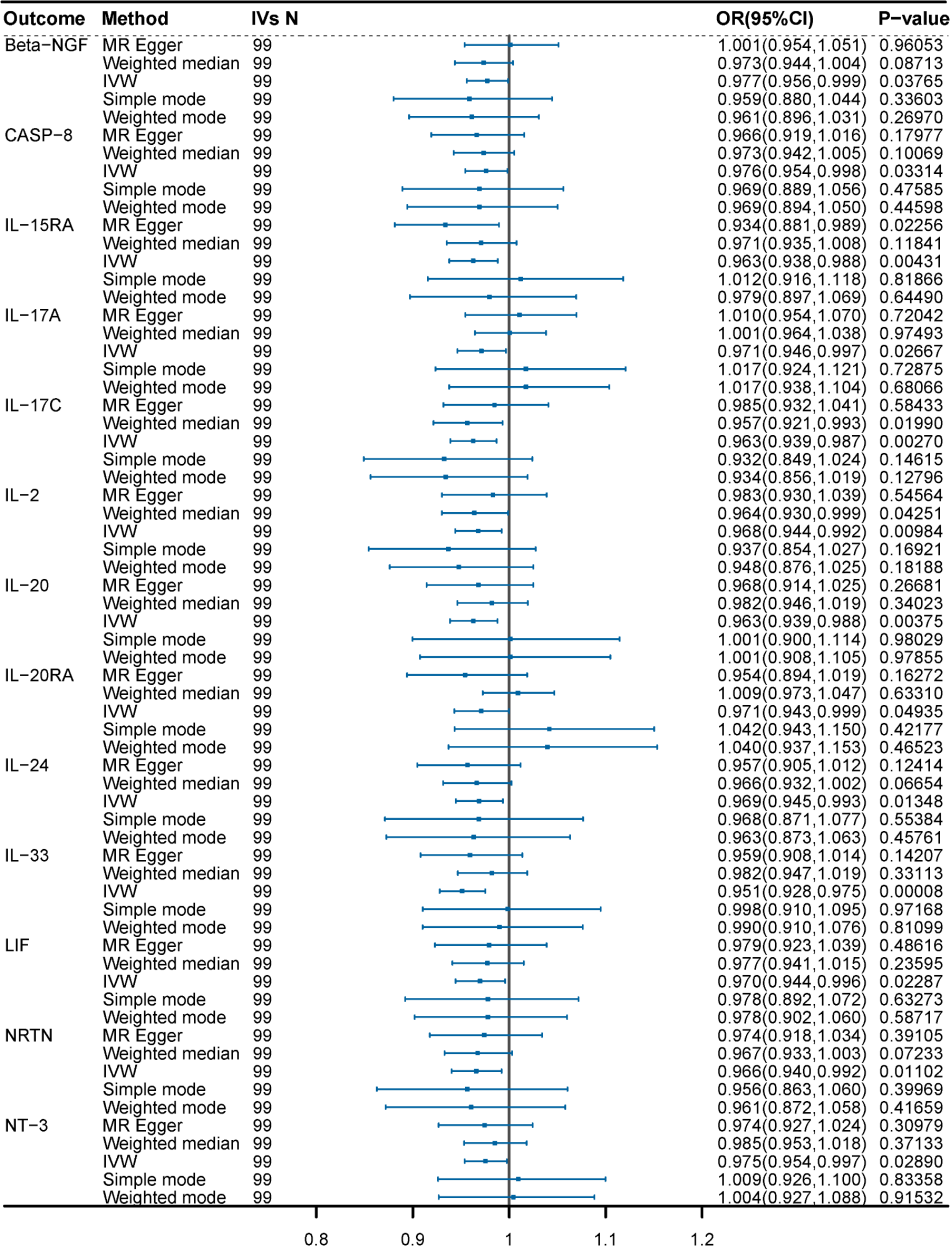
MR analysis of plasma BDNF on 13 circulating inflammatory proteins by IVW method (*P* < 0.05).

**Fig. 4:**
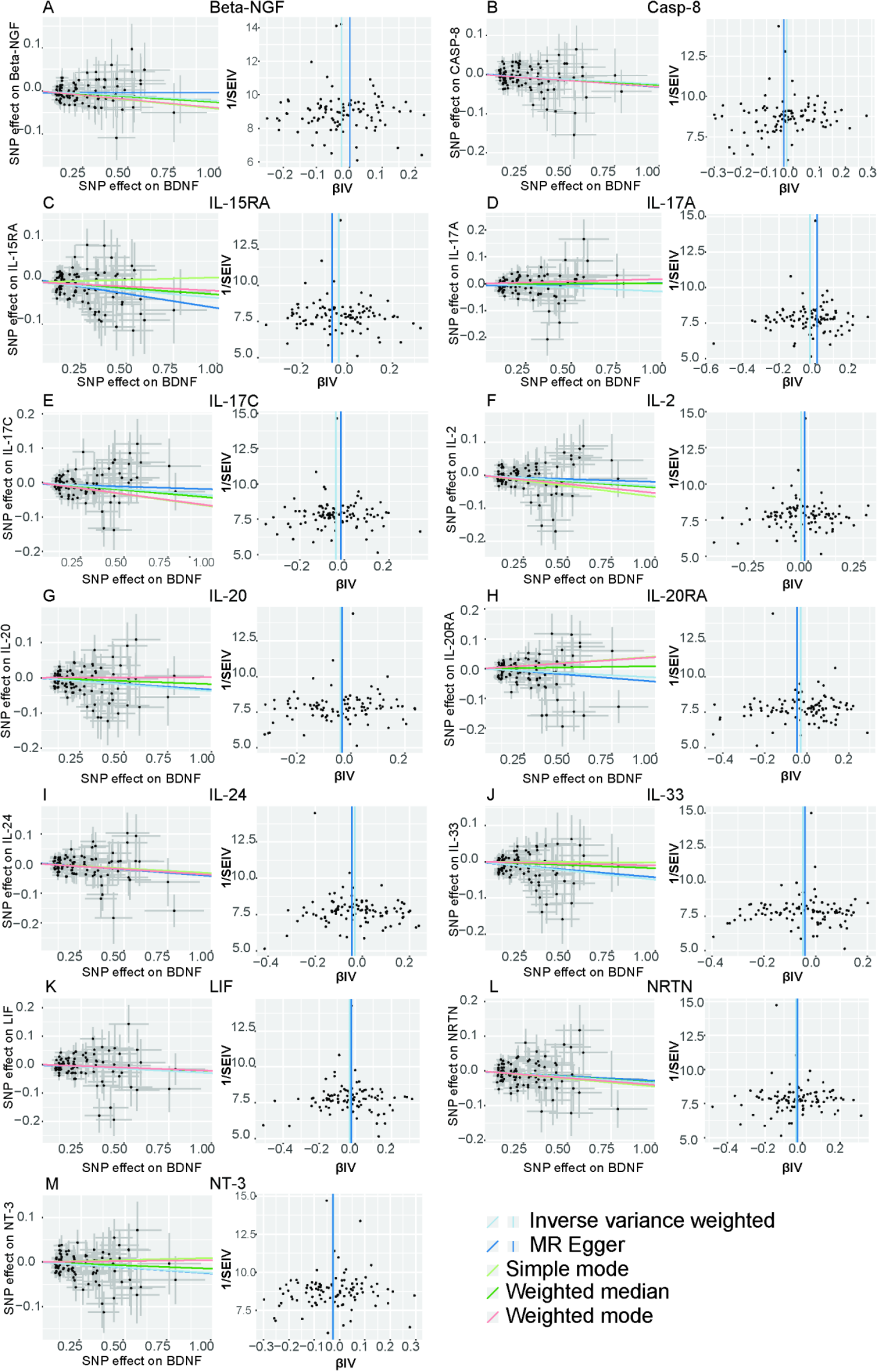
Scatter and funnel plots of MR analyses for plasma BDNF in 13 circulating inflammatory proteins. Scatter plots illustrate individual IV associations with plasma BDNF versus individual IV associations with 13 circulating inflammatory proteins in black dots and the funnel plots illustrate the IVW (blue line) and MR-Egger (dark blue line) MR estimate of plasma BDNF SNP with 13 circulating inflammatory proteins (A-M).

To investigate the causal effect of genetically predicted BDNF plasma levels on the 91 circulating inflammatory proteins (outcome factors), a two-sample MR analysis was performed (Fig. 2, Supplement Table S1). Using the IVW method, we identified that genetically predicted plasma BDNF levels were associated with 13 of the 91 outcome factors (Fig. 3). Specifically, genetically predicted high plasma BDNF level was significantly associated with decreased levels of Beta-NGF (OR = 0.977, 95% CI = 0.956–0.999, *P* = 0.03765, P_FDR_=0.28555), CASP-8 (OR = 0.976, 95% CI = 0.954–0.998, *P* = 0.03314, P_FDR_=0.27416), IL-2 (OR = 0.968, 95% CI = 0.944–0.992, *P* = 0.01269, P_FDR_=0.16721), IL-15RA (OR = 0.963, 95% CI = 0.938–0.988, *P* = 0.00431, P_FDR_=), IL-17 A (OR = 0.971, 95% CI = 0.946–0.997, *P* = 0.02667, P_FDR_=0.26299), IL-17 C (OR = 0.963, 95% CI = 0.939–0.987, *P* = 0.0027, P_FDR_=0.09809), IL-20 (OR = 0.963, 95% CI = 0.939–0.988, *P* = 0.00375, P_FDR_=0.09809), IL-20RA (OR = 0.971, 95% CI = 0.943–0.999, *P* = 0.04935, P_FDR_=0.34543), IL-24 (OR = 0.969, 95% CI = 0.945–0.993, *P* = 0.01348, P_FDR_=0.17520), IL-33 (OR = 0.951, 95% CI = 0.928–0.975, *P* = 0.00008, P_FDR_=0.00719), LIF (OR = 0.970, 95% CI = 0.944–0.996, *P* = 0.02287, P_FDR_=0.26018), NRTN (OR = 0.966, 95% CI = 0.940–0.992, *P* = 0.01102, P_FDR_=0.16721), as well as NT-3 (OR = 0.975, 95% CI = 0.954–0.997, *P* = 0.02890, P_FDR_=0.26299). The associations between BDNF and IL-33 remained statistically significant after FDR correction (FDR > 0.05). Supplementary Table S2 presents IVs for the BDNF plasma levels (*P* < 5 × 10^− 5^). MR-Egger regression, weighted median, weighted mode, and simple mode analyses also show the same trend, enhancing the overall robustness of the findings (Fig. 4). MR-Egger and MR-PRESSO tests (Supplement Table S3) revealed no evidence of horizontal pleiotropy, and no obvious heterogeneity was found according to Cochrane’s Q test. SNP effect sizes for predicted BDNF were visualized in scatter plots (Fig. 4) for the inflammatory proteins, and the forest plot (Supplementary Figure S1-S14) showed the causal association of each BDNF-related SNP on inflammatory proteins, and funnel plots (Fig. 4) revealed no significant heterogeneity among selected IVs (Supplementary Figure S1-S39). Leave-one-out analysis showed that no single SNP was driving the bias of the estimates (Supplementary Figure S15-S26).

## The causal effect of inflammatory proteins on BDNF levels

**Fig. 5:**
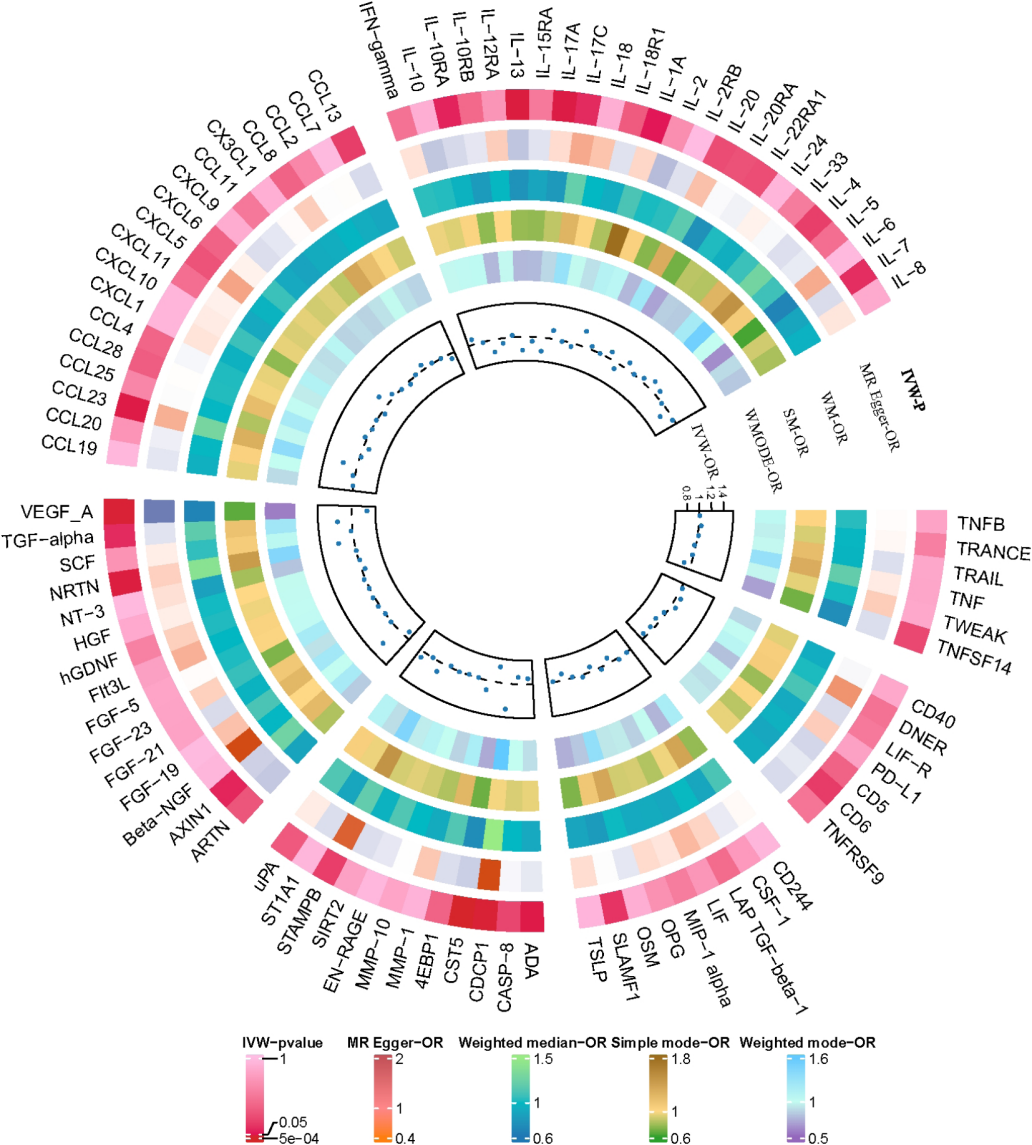
Circle diagram of 91 circulating inflammatory proteins on plasma BDNF using the five methods (IVW, MR-Egger, weighted median, weighted mode, and simple mode).

**Fig. 6:**
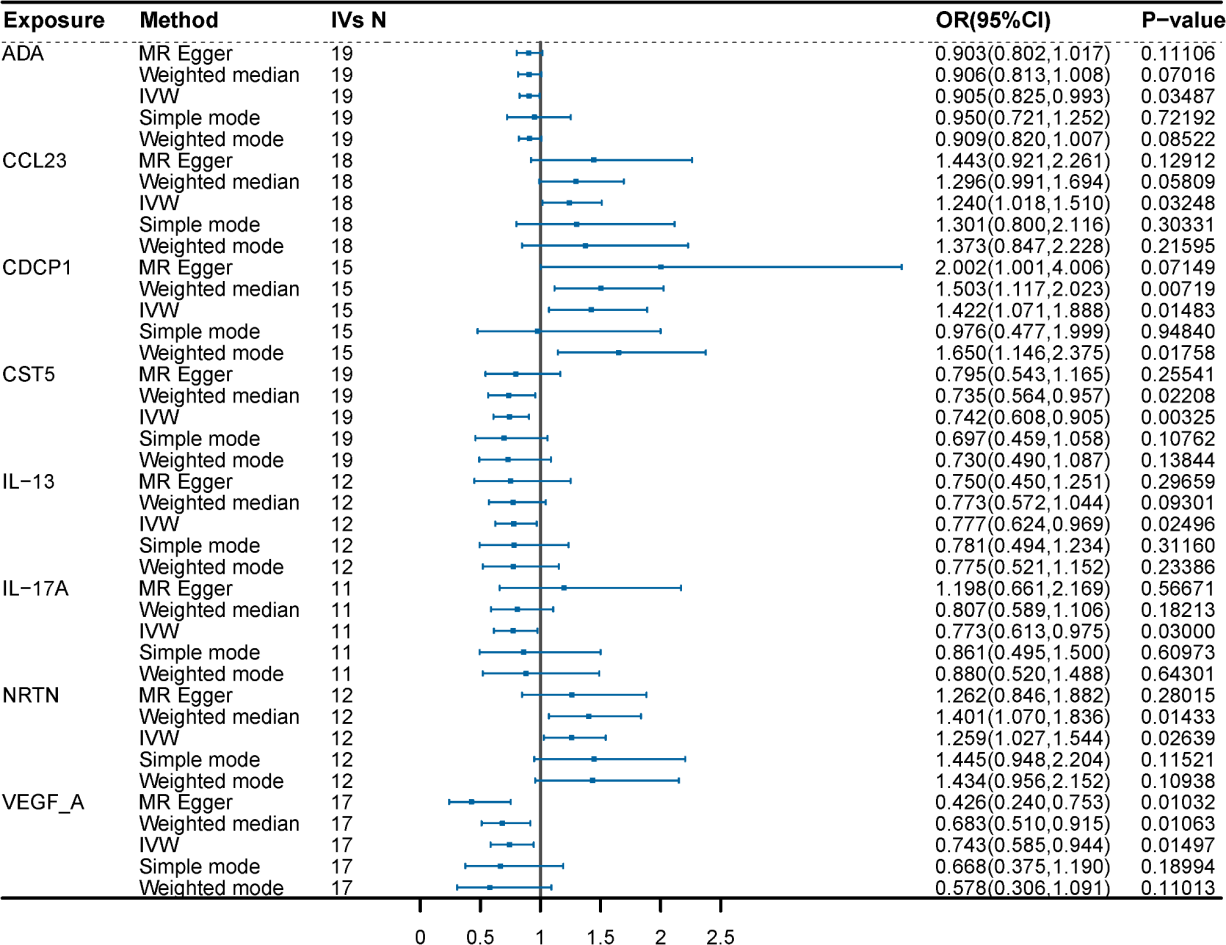
MR analysis of 8 circulating inflammatory proteins on plasma BDNF by IVW method (*P* < 0.05).

**Fig. 7:**
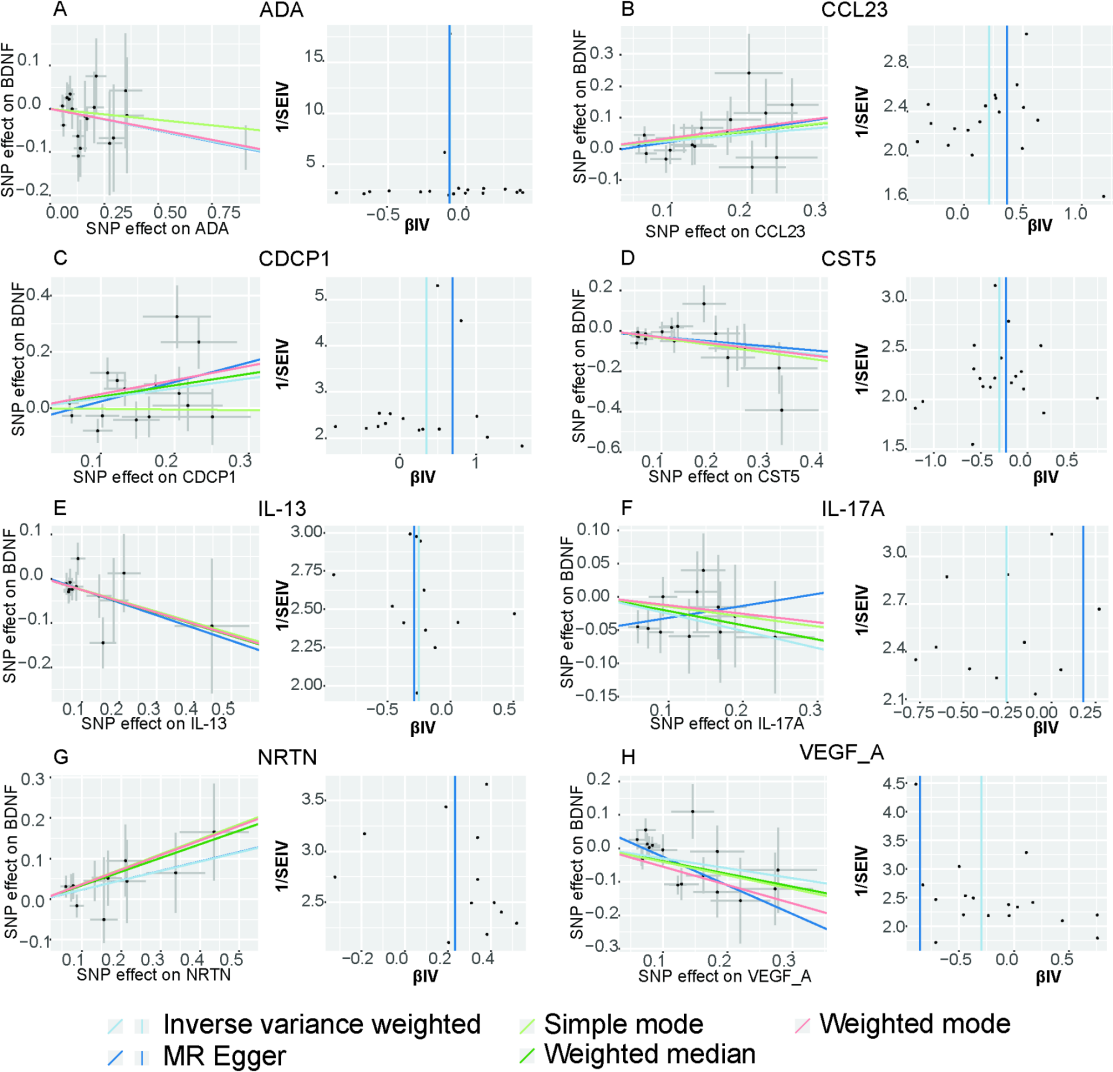
Scatter and funnel plots of MR analyses for 8 circulating inflammatory proteins in plasma BDNF. Scatter plots illustrate individual IV associations with 8 circulating inflammatory proteins versus individual IV associations with plasma BDNF in black dots AND the funnel plots illustrate the IVW (blue line) and MR-Egger (dark blue line) MR estimate of 8 circulating inflammatory proteins SNP with plasma BDNF proteins (A-H).

When considering 91 circulating inflammatory factors as exposures and the BDNF levels as outcomes, reverse MR analysis results are in Fig. 5 and Supplement Table S4. The IVW results showed a statistically significant negative correlation between plasma levels of ADA (OR = 0.905, 95% CI = 0.825–0.993, *P* = 0.03487, P_FDR_=0.39667), CST5 (OR = 0.742, 95% CI = 0.608–0.905, *P* = 0.00325, P_FDR_=0.29545), IL-13 (OR = 0.777, 95% CI = 0.624–0.969, *P* = 0.02496, P_FDR_=0.39667), IL-17 A (OR = 0.773, 95% CI = 0.613–0.975, *P* = 0.0300, P_FDR_=0.39667), VEGF-A (OR = 0.971, 95% CI = 0.943–0.999, *P* = 0.01497, P_FDR_=0.39667) and those of BDNF. The results also showed positive associations between elevated levels of CCL23 (OR = 1.240, 95% CI = 1.018,1.510, *P* = 0.03248, P_FDR_=0.39667), CDCP1 (OR = 1.422, 95% CI = 1.071–1.888, *P* = 0.01483, P_FDR_=0.39667), NRTN (OR = 1.259, 95% CI = 1.027–1.544, *P* = 0.02639, P_FDR_=0.39667) and increased BDNF levels in plasma (Fig. 6). However, the reliability of the genetically predicted effect of these 8 circulating inflammatory proteins on BDNF identified in the reverse MR analysis is not confirmed (FDR > 0.05). Supplementary Table S5 presents IVs for the 91 circulating inflammatory factors (*P* < 1 × 10^− 5^). The same trend was observed in MR-Egger regression, weighted median, weighted mode, and simple mode analyses, enhancing the overall robustness of the findings (Fig. 7). MR-Egger and MR-PRESSO tests (Supplement Table S6) revealed no evidence of horizontal pleiotropy, and no obvious heterogeneity was found according to Cochrane’s Q test. SNP effect sizes for predicted inflammatory proteins were visualized in scatter plots (Fig. 7) for the BDNF, and the forest plot (Supplementary Figure S27-S34) showed the causal association of each inflammatory proteins-related SNP on BDNF, and funnel plots (Fig. 7) revealed no significant heterogeneity among selected IVs. Leave-one-out analysis showed that no single SNP was driving the bias of the estimates (Supplementary Figure S35-S42).

## Discussion

In this study, we conducted a bidirectional MR study that offers a genetic insight into the potential causal relationship between plasma BDNF and 91 circulating inflammatory proteins. Our findings suggest that genetically predicted high plasma BDNF levels exhibited decreased levels of Beta-NGF, CASP-8, IL-2, IL-15RA, IL-17 A, IL-17 C, IL-20, IL-20RA, IL-24, IL-33, LIF, NRTN, and NT-3, with CDCP1 representing the strongest positive correlation based on standardized MR analysis. Furthermore, levels of CCL23, CDCP1, and NRTN are positively associated with the BDNF level. Conversely, CST5 exhibits the strongest negative association with BDNF levels, along with other factors such as ADA, IL-13, IL-17 A, and VEGF-A, which are also inversely related to BDNF levels. The sensitivity analyses support the robustness of these results.

BDNF is the most widely distributed neurotrophin in the CNS and plays an important role in neurological diseases in which neuroinflammation appears to be highly implicated^50,51^. Traditionally, clinical studies quantifying BDNF levels were limited to measuring it in blood or cerebral spinal fluid^52,53^. Moreover, direct measurements of BDNF mRNA or protein in the brain can only be taken post-mortem^54^. However, BDNF may indirectly cross the blood-brain barrier (BBB) by retrograde axonal transport^55,56^, and peripheral BDNF levels reflect central BDNF levels^57^. Accordingly, peripheral BDNF levels are a feasible indicator of central protein expression. Since BDNF plays a critical role in the regeneration, survival, and maintenance of neuronal function^58^, a relative increase in BDNF may act as a compensatory mechanism to minimize neuronal damage. However, schizophrenia patients are unable to produce sufficient amounts of BDNF to mitigate the inflammatory damage induced by IL-2^59^, and a negative correlation between BDNF and IL-2 levels was observed in our study. Similarly, data from a cross-sectional study have shown that first-episode depression and recurrent depressive disorder are associated with lower serum concentrations of BDNF and higher IL-2 levels^60^. IL-17 A, a pro-inflammatory cytokine, is the hallmark cytokine of T helper 17 (Th17) cell subset and is produced by Th17 cells, NK cells, NKT cells, and γδ-T cells^61^. In spinal cord injury patients, a higher level of BDNF was observed in the plasma within 24 h, and the elevated level of BDNF was strongly positively correlated with the percentage of NK cells, while, the production of IL-17 A was reduced during the early period of spinal cord injury^62^, which parallels our observations. LPS injection upregulated IL-33 expression in the amygdala, whereas IL-33 deficiency attenuated LPS-induced anxiety-like behaviour in mice via the γ-aminobutyric acid (GABA) circuit between the amygdala and the prefrontal cortex^63^. Moreover, BDNF levels were elevated in the amygdala of IL-33 deficient mice, while IL-33 suppressed BDNF expression through the NF-κB pathway^63^, which is consistent with our findings. In AD, the accumulation of β-amyloid (Aβ) leads to the formation of neuritic plaques outside nerve cells and neurofibrillary tangles inside nerve cells by hyperphosphorylated tau protein, disrupting physiological functions and resulting in synaptic dysfunction, neuronal loss, and microglial activation^64^; concomitantly, BDNF level was decreased in AD patients^65,66^. Exogenous IL-33 administration significantly polarized microglia toward an anti-inflammatory phenotype, reduced pro-inflammatory gene expression, and enhanced the phagocytic activity of microglia, thereby improving Aβ pathology in AD mice by promoting the recruitment of microglia and decreasing amyloid plaque deposition^67,68^. However, clinical observational studies have shown that AD patients with high IL-33 expression do not have lower levels of Aβ and tau^69^. Beta-NGF is a neurotrophic factor and neuropeptide primarily, which is decreased in major depressive disorder patients after antidepressant pharmacotherapy, while BDNF levels are increased^70^. Similarly, lower BDNF and higher NT-3 serum levels are observed in depressed patients with schizophrenia^71^. Chronic stress reduced BDNF mRNA levels and increased NT-3 mRNA levels in the hippocampus^72^. Oxidative stress and inflammation are associated with the late phase of stroke, leading to the activation of CASP8 and increased CASP8 activity levels, whereas BDNF levels remain low^73,74^. The balance of BDNF and these factors may play a crucial role in inflammation-related neurological diseases.

MR results in this study indicate that genetically predicted high plasma BDNF levels are associated with decreased levels of pro-inflammatory makers such as IL-15RA, IL-17 C, IL-20, IL-20RA, IL-24, LIF, and NRTN. In reverse MR analyses, the levels of CCL23, CDCP1, and NRTN are positively associated, while ADA, CST5, IL-13, IL-17 A, and VEGF-A are negatively associated with the BDNF levels. So far the relationship between BDNF and these factors has not yet been addressed. IL-15RA, a high-affinity IL-15 binding protein expressed by glial cells and neurons throughout the brain, is essential for mediating IL-15 signalling^75,76^. Increased intrathecal production of IL-15 is observed in patients with multiple sclerosis^77^. Few papers describe the role of IL-17 C in humans or mice, and none involve the CNS^78,79^, Waisman, et al. concluded in their review that IL-17 C may indirectly contribute to CNS inflammation by acting on T cells rather than directly affecting the CNS^80^. The role of IL-20, IL-20RA, and IL-24 in the CNS remains unclear despite the reported expression by glial cells and an upregulation of IL-20 in the ischemic brain that may contribute to brain injury^81^. LIF is increasingly recognized as a potential therapeutic target for multiple sclerosis, as elevated expression of LIF receptor enhances regulatory T cell numbers, thereby contributing to the hyperactive immune system characteristic of the disease^82^. NRTN is a member of the glial cell line-derived neurotrophic factor family, which has been demonstrated to notably enhance dopaminergic neuron survival and behavioural function in animal models^83,84^. CCL23, a chemokine implicated in inflammation and host defence responses, has been frequently reported as a potential blood biomarker for mild cognitive impairment to AD progression^85^. Not uniquely, some research groups have considered CCL23 as a novel CC chemokine involved in human brain injury and a promising biomarker of injury in ischemic stroke patients^86,87^. Elevated level of CDCP1 was associated with higher risks of incident all-cause and AD dementia^88^. Adenosine deaminase acting on RNA1 (ADAR1) is a member of the ADAR family, which inducer alleviates the depressive-like behavior of chronic unpredictable stress mice by rescuing the decreased BDNF protein in the prefrontal cortex, hippocampus, and serum^89^. A decrease in plasma ADA activity in patients with major depression, which correlated negatively with the severity of the illness, suggested that adenosine might be involved in the pathological changes associated with major depression^90^. ADA CST5 was identified as the optimal biomarker for traumatic brain injury (TBI) due to its ability to differentiate between mild and severe injuries within the first hour of injury, effectively identifying patients with severe TBI from all other cohorts^91^. VEGF was noted to indirectly promote neurogenic effects by stimulating endothelial cell production and the release of BDNF, thereby supporting the survival and integration of new-born neurons in the adult songbird neostriatum^92^. IL-13 possesses anti-inflammatory properties and is markedly decreased in patients with acute severe stroke^93^. The results of this study suggest that genetically predicted high plasma BDNF levels might influence various inflammatory and neurotrophic factors, potentially impacting neurological health and disease. Specifically, reduced levels of certain interleukins and neurotrophic factors could affect immune signalling and brain injury responses. Increased levels of chemokines and other proteins linked to cognitive decline and dementia could highlight new biomarkers for these conditions.

IL-6 and C-reactive protein (CRP) are widely recognized as key biomarkers for neuroinflammation. However, our findings revealed no significant causal relationship between IL-6, CRP, and BDNF. Clinical studies investigating the causal relationships or interactions between the circulating cytokines IL-6 and CRP, and BDNF in neurological diseases (AD^94^ and HD^95^) yielded inconsistent findings. Circulating levels of IL-6, CRP, and BDNF in AD patients have been controversial for several decade, and its significance regarding AD etiology remains unclear. Previous study suggested that there were no significant differences in IL-6 levels between the control group and both mild to moderate AD patients and severe AD patients^96^. While some studies reported higher plasma IL-6 levels in AD^97^, the relationship between AD severity and circulating IL-6 levels has been inconsistent^98^. Additionally, no differences in serum CRP and BDNF levels were observed between AD patients and control groups^99^. Post-mortem studies have revealed a reduction in BDNF levels and expression in several brain regions of AD patients, especially in the hippocampus and cortex^65,66^. However, other studies have reported inconsistent findings^100,101^. Since BDNF in the bloodstream is primarily found in platelets and released during clotting when serum is obtained^102^, variations in the release of BDNF from these platelets could explain inconsistencies among different studies. Furthermore, confounding elements, such as unreported drugs usage, comorbid conditions, and sleep issues, may also contribute to the variability in measured BDNF and circulating cytokines levels. In addition, methodological factors like the timing of blood collection, the delay between sample collection and analysis, and the effects of freeze-thaw cycles can also impact the results. Plasma IL-6 levels were elevated in HD patients across all stages of the disease and increased with disease progression^103^. A study on cancer-related cognitive impairment also found a negative correlation between BDNF and plasma IL-6 levels^104^. Conversely, Wertz et al.,^105^ demonstrated that IL-6 deficiency exacerbated behavioral phenotypes in an HD mouse model, leading to the dysregulation of several genes associated with synaptic function, including the BDNF receptor Ntrk2. These findings suggested that IL-6 may have played an early protective role in HD. A significant elevation in CRP levels was observed in pre-manifest HD patients compared to the control group, but this difference was not seen in those with manifest HD^106^. No significant difference in plasma BDNF was observed between the PD group and healthy controls^106^. In contrast, another study indicated that CRP levels were reduced in early HD^107^.

Our study is highly relevant for the following reasons: The two-sample MR design largely avoided confounding bias, reverse causation, and various biases compared with traditional observational studies. The population of exposure and outcome datasets are non-overlapping individuals of European ancestry, which can reduce the ethnic differences affecting the polymorphism of genes. A total of five MR analysis methods were applied to the evaluation of the consistency of causal effects. Multiple sensitivity analyses including Cochran’s Q, MR Egger, and MR PRESSO utilized in this MR study yielded robust evidence. Whereas, some limitations exist in this study. Since most of the participants in GWAS are of European origin, this may limit the extrapolation of research results to other populations. Clinical trials are not currently designed to test of association between BDNF and inflammation factors, making it difficult to evaluate the power of MR study. Insufficient data, including age and gender, are available, which hinders in-depth analysis. Additionally, confounding factors could arise if these genetic variants are associated with other unmeasured variables that in turn influence the outcome. Furthermore, these variants can correlate with various environmental or lifestyle factors that also impact the outcome, introducing potential common causes that violate the assumption of independence and can lead to biased estimates. More animal or human trials are needed to explore the relationship between BDNF and inflammation.

## Conclusion

In summary, the MR analysis indicated that genetically predicted plasma BDNF levels are causally linked to IL-33, and possibly to 12 circulating inflammatory proteins (Beta-NGF, CASP-8, IL-2, IL-15RA, IL-17 A, IL-17 C, IL-20, IL-20RA, IL-24, LIF, NRTN, and NT-3) shown to be differently involved in the pathogenesis or outcome of several neurological diseases. Reverse analysis suggested 3 inflammatory proteins (CCL23, CDCP1, and NRT) to be positively correlated with BDNF levels, which may enhance immune responses, contribute to neuroprotection, and support neuron survival, and 5 proteins (ADA, CST5, IL-13, IL-17 A, and VEGF-A) likely to be negatively correlated with BDNF levels that could reduce inflammation, affect brain injury recovery, and impair angiogenesis and neurogenesis. Our study offers new insights into the role of BDNF in regulating the levels of circulating inflammatory proteins and paves the way for future research and the development of therapeutic strategies in inflammation-related neurological diseases.

## Electronic supplementary material

Below is the link to the electronic supplementary material.

Supplementary Material 1

Supplementary Material 2

## Data Availability

The data analyzed in this study are all publicly available. The GWAS data source for plasma BDNF levels (accession numbers GCST90240466) can be downloaded from the GWAS Catalog (https://www.ebi.ac.uk/gwas/). The GWAS summary statistics for 91 inflammatory proteins are publicly available in the EBI GWAS Catalog (accession numbers GCST90274758 to GCST90274848) and can be downloaded from https://www.phpc.cam.ac.uk/ceu/proteins.

## References

[1] Leschik, J. et al. Brain-derived neurotrophic factor expression in serotonergic neurons improves stress resilience and promotes adult hippocampal neurogenesis. *Prog Neurobiol.* **217**, 102333. 10.1016/j.pneurobio.2022.102333 (2022). 35872219 10.1016/j.pneurobio.2022.102333

[2] Scharfman, H. et al. Increased neurogenesis and the ectopic granule cells after intrahippocampal BDNF infusion in adult rats. *Exp. Neurol.* **192**, 348–356. 10.1016/j.expneurol.2004.11.016 (2005). 15755552 10.1016/j.expneurol.2004.11.016

[3] Han, B. H. & Holtzman, D. M. BDNF protects the neonatal brain from hypoxic-ischemic injury in vivo via the ERK pathway. *J. Neurosci.* **20**, 5775–5781. 10.1523/jneurosci.20-15-05775.2000 (2000). 10908618 10.1523/JNEUROSCI.20-15-05775.2000 PMC6772561

[4] Zagrebelsky, M. & Korte, M. Are TrkB receptor agonists the right tool to fulfill the promises for a therapeutic value of the brain-derived neurotrophic factor? *Neural Regen Res.* **19**, 29–34. 10.4103/1673-5374.374138 (2024). 37488840 10.4103/1673-5374.374138 PMC10479861

[5] Kellner, Y. et al. The BDNF effects on dendritic spines of mature hippocampal neurons depend on neuronal activity. *Front. Synaptic Neurosci.* **6**, 5. 10.3389/fnsyn.2014.00005 (2014). 24688467 10.3389/fnsyn.2014.00005 PMC3960490

[6] Zagrebelsky, M., Tacke, C. & Korte, M. BDNF signaling during the lifetime of dendritic spines. *Cell. Tissue Res.* **382**, 185–199. 10.1007/s00441-020-03226-5 (2020). 32537724 10.1007/s00441-020-03226-5 PMC7529616

[7] Korte, M. et al. Hippocampal long-term potentiation is impaired in mice lacking brain-derived neurotrophic factor. *Proc. Natl. Acad. Sci. U S A*. **92**, 8856–8860. 10.1073/pnas.92.19.8856 (1995). 7568031 10.1073/pnas.92.19.8856 PMC41066

[8] Erickson, K. I. et al. Brain-derived neurotrophic factor is associated with age-related decline in hippocampal volume. *J. Neurosci.* **30**, 5368–5375. 10.1523/jneurosci.6251-09.2010 (2010). 20392958 10.1523/JNEUROSCI.6251-09.2010 PMC3069644

[9] Amidfar, M., de Oliveira, J., Kucharska, E., Budni, J. & Kim, Y. K. The role of CREB and BDNF in neurobiology and treatment of Alzheimer’s disease. *Life Sci.* **257**, 118020. 10.1016/j.lfs.2020.118020 (2020). 32603820 10.1016/j.lfs.2020.118020

[10] Kang, S. S., Wu, Z., Liu, X., Edgington-Mitchell, L. & Ye, K. Treating Parkinson’s disease via activation of BDNF/TrkB signaling pathways and Inhibition of Delta-Secretase. *Neurotherapeutics* **19**, 1283–1297. 10.1007/s13311-022-01248-1 (2022). 35595958 10.1007/s13311-022-01248-1 PMC9587159

[11] Zuccato, C. & Cattaneo, E. Role of brain-derived neurotrophic factor in Huntington’s disease. *Prog Neurobiol.* **81**, 294–330. 10.1016/j.pneurobio.2007.01.003 (2007). 17379385 10.1016/j.pneurobio.2007.01.003

[12] Lommatzsch, M. et al. The impact of age, weight and gender on BDNF levels in human platelets and plasma. *Neurobiol. Aging*. **26**, 115–123. 10.1016/j.neurobiolaging.2004.03.002 (2005). 15585351 10.1016/j.neurobiolaging.2004.03.002

[13] Fujimura, H. et al. Brain-derived neurotrophic factor is stored in human platelets and released by agonist stimulation. *Thromb. Haemost*. **87**, 728–734 (2002). 12008958

[14] Nagata, T. et al. Plasma BDNF levels are correlated with aggressiveness in patients with amnestic mild cognitive impairment or alzheimer disease. *J. Neural Transm (Vienna)*. **121**, 433–441. 10.1007/s00702-013-1121-y (2014). 24253237 10.1007/s00702-013-1121-y

[15] Kim, Y. K. et al. Low plasma BDNF is associated with suicidal behavior in major depression. *Prog Neuropsychopharmacol. Biol. Psychiatry*. **31**, 78–85. 10.1016/j.pnpbp.2006.06.024 (2007). 16904252 10.1016/j.pnpbp.2006.06.024

[16] Klein, A. B. et al. Blood BDNF concentrations reflect brain-tissue BDNF levels across species. *Int. J. Neuropsychopharmacol.* **14**, 347–353. 10.1017/s1461145710000738 (2011). 20604989 10.1017/S1461145710000738

[17] Yarube, I. U. et al. Association between cognition and peripheral brain-derived neurotrophic factor in a sample of normal adults in Kano, Nigeria. *Nigerian J. Basic. Clin. Sci.* **16**, 55–59 (2019).

[18] Harris, A. P. et al. The role of brain-derived neurotrophic factor in learned fear processing: an awake rat fMRI study. *Genes Brain Behav.* **15**, 221–230. 10.1111/gbb.12277 (2016). 26586578 10.1111/gbb.12277 PMC4819698

[19] Voet, S., Srinivasan, S., Lamkanfi, M. & van Loo, G. Inflammasomes in neuroinflammatory and neurodegenerative diseases. *EMBO Mol. Med.* **11**10.15252/emmm.201810248 (2019). 10.15252/emmm.201810248 PMC6554670 31015277

[20] Stephenson, J., Nutma, E., van der Valk, P. & Amor, S. Inflammation in CNS neurodegenerative diseases. *Immunology* **154**, 204–219. 10.1111/imm.12922 (2018). 29513402 10.1111/imm.12922 PMC5980185

[21] Ransohoff, R. M. How neuroinflammation contributes to neurodegeneration. *Science* **353**, 777–783. 10.1126/science.aag2590 (2016). 27540165 10.1126/science.aag2590

[22] Subhramanyam, C. S., Wang, C., Hu, Q. & Dheen, S. T. Microglia-mediated neuroinflammation in neurodegenerative diseases. *Semin Cell. Dev. Biol.* **94**, 112–120. 10.1016/j.semcdb.2019.05.004 (2019). 31077796 10.1016/j.semcdb.2019.05.004

[23] Wu, S. Y. et al. BDNF reverses aging-related microglial activation. *J. Neuroinflammation*. **17**, 210. 10.1186/s12974-020-01887-1 (2020). 32664974 10.1186/s12974-020-01887-1 PMC7362451

[24] Makar, T. K. et al. Brain derived neurotrophic factor treatment reduces inflammation and apoptosis in experimental allergic encephalomyelitis. *J. Neurol. Sci.* **270**, 70–76. 10.1016/j.jns.2008.02.011 (2008). 18374360 10.1016/j.jns.2008.02.011

[25] Jiang, Y. et al. Intranasal brain-derived neurotrophic factor protects brain from ischemic insult via modulating local inflammation in rats. *Neuroscience* **172**, 398–405. 10.1016/j.neuroscience.2010.10.054 (2011). 21034794 10.1016/j.neuroscience.2010.10.054

[26] Jiang, Y. et al. Effects of brain-derived neurotrophic factor on local inflammation in experimental stroke of rat. *Mediators Inflamm.* **2010**, 372423. 10.1155/2010/372423 (2010). 21490702 10.1155/2010/372423 PMC3068595

[27] Han, R. et al. BDNF alleviates neuroinflammation in the hippocampus of type 1 diabetic mice via blocking the aberrant HMGB1/RAGE/NF-κB pathway. *Aging Dis.* **10**, 611–625. 10.14336/ad.2018.0707 (2019). 31165005 10.14336/AD.2018.0707 PMC6538223

[28] Ventriglia, M. et al. Serum brain-derived neurotrophic factor levels in different neurological diseases. *Biomed. Res. Int.* **2013**, 901082. 10.1155/2013/901082 (2013). 24024214 10.1155/2013/901082 PMC3760208

[29] Zhang, X. et al. Positive feedback loop of autocrine BDNF from microglia causes prolonged microglia activation. *Cell. Physiol. Biochem.* **34**, 715–723. 10.1159/000363036 (2014). 25171395 10.1159/000363036

[30] Ding, H. et al. BDNF promotes activation of astrocytes and microglia contributing to neuroinflammation and mechanical allodynia in cyclophosphamide-induced cystitis. *J. Neuroinflammation*. **17**, 19. 10.1186/s12974-020-1704-0 (2020). 31931832 10.1186/s12974-020-1704-0 PMC6958761

[31] Yasutake, C. et al. TNF-alpha and IL-1beta levels in dementia patients: comparison between Alzheimer’s disease and vascular dementia. *Eur. Arch. Psychiatry Clin. Neurosci.* **256**, 402–406. 10.1007/s00406-006-0652-8 (2006). 16783499 10.1007/s00406-006-0652-8

[32] Emdin, C. A., Khera, A. V., Kathiresan, S. & Mendelian Randomization *Jama* ;**318**:1925–1926. doi: 10.1001/jama.2017.17219 (2017). 29164242 10.1001/jama.2017.17219

[33] Wang, X. et al. Causal association between serum Thyrotropin and obesity: A bidirectional, Mendelian randomization study. *J. Clin. Endocrinol. Metab.* **106**, e4251–e4259. 10.1210/clinem/dgab183 (2021). 33754627 10.1210/clinem/dgab183 PMC8475201

[34] Chen, S. et al. Exploring the causality between plasma Brain-Derived neurotrophic factor and neurological diseases: A Mendelian randomization study. *J. Alzheimers Dis.* **96**, 135–148. 10.3233/jad-230693 (2023). 37742652 10.3233/JAD-230693

[35] You, J. et al. Association between plasma brain-derived neurotrophic factor level and Alzheimer’s disease: a Mendelian randomization study. *Curr. Neurovasc Res.*10.2174/0115672026281995231227070637 (2024). 38279765 10.2174/0115672026281995231227070637

[36] Wang, W. et al. Relationship between plasma brain-derived neurotrophic factor levels and neurological disorders: an investigation using Mendelian randomisation. *Heliyon* **10**, e30415. 10.1016/j.heliyon.2024.e30415 (2024). 38707431 10.1016/j.heliyon.2024.e30415 PMC11068855

[37] Skrivankova, V. W. et al. Strengthening the reporting of observational studies in epidemiology using Mendelian randomization: the STROBE-MR statement. *Jama* **326**, 1614–1621. 10.1001/jama.2021.18236 (2021). 34698778 10.1001/jama.2021.18236

[38] Sun, B. B. et al. Genomic atlas of the human plasma proteome. *Nature* **558**, 73–79. 10.1038/s41586-018-0175-2 (2018). 29875488 10.1038/s41586-018-0175-2 PMC6697541

[39] Zhao, J. H. et al. Genetics of Circulating inflammatory proteins identifies drivers of immune-mediated disease risk and therapeutic targets. *Nat. Immunol.* **24**, 1540–1551. 10.1038/s41590-023-01588-w (2023). 37563310 10.1038/s41590-023-01588-w PMC10457199

[40] Panagiotou, O. A. & Ioannidis, J. P. What should the genome-wide significance threshold be? Empirical replication of borderline genetic associations. *Int. J. Epidemiol.* **41**, 273–286. 10.1093/ije/dyr178 (2012). 22253303 10.1093/ije/dyr178

[41] Liu, Y., Zhang, Y., Du, L. & Chen, D. The genetic relationships between immune cell traits, Circulating inflammatory proteins and preeclampsia/eclampsia. *Front. Immunol.* **15**, 1389843. 10.3389/fimmu.2024.1389843 (2024). 38873604 10.3389/fimmu.2024.1389843 PMC11170637

[42] Zhao, D., Sui, Y. & Sun, Y. Causal relationship between immune cells and hypopituitarism: bidirectional Mendelian randomization study. *World Neurosurg.* **194**, 123411. 10.1016/j.wneu.2024.10.140 (2025). 39522807 10.1016/j.wneu.2024.10.140

[43] Pierce, B. L., Ahsan, H. & Vanderweele, T. J. Power and instrument strength requirements for Mendelian randomization studies using multiple genetic variants. *Int. J. Epidemiol.* **40**, 740–752. 10.1093/ije/dyq151 (2011). 20813862 10.1093/ije/dyq151 PMC3147064

[44] Burgess, S., Butterworth, A. & Thompson, S. G. Mendelian randomization analysis with multiple genetic variants using summarized data. *Genet. Epidemiol.* **37**, 658–665. 10.1002/gepi.21758 (2013). 24114802 10.1002/gepi.21758 PMC4377079

[45] Verbanck, M., Chen, C. Y., Neale, B. & Do, R. Detection of widespread horizontal Pleiotropy in causal relationships inferred from Mendelian randomization between complex traits and diseases. *Nat. Genet.* **50**, 693–698. 10.1038/s41588-018-0099-7 (2018). 29686387 10.1038/s41588-018-0099-7 PMC6083837

[46] Bowden, J., Davey Smith, G. & Burgess, S. Mendelian randomization with invalid instruments: effect Estimation and bias detection through Egger regression. *Int. J. Epidemiol.* **44**, 512–525. 10.1093/ije/dyv080 (2015). 26050253 10.1093/ije/dyv080 PMC4469799

[47] Burgess, S., Scott, R. A., Timpson, N. J., Davey Smith, G. & Thompson, S. G. Using published data in Mendelian randomization: a blueprint for efficient identification of causal risk factors. *Eur. J. Epidemiol.* **30**, 543–552. 10.1007/s10654-015-0011-z (2015). 25773750 10.1007/s10654-015-0011-z PMC4516908

[48] Burgess, S., Dudbridge, F. & Thompson, S. G. Combining information on multiple instrumental variables in Mendelian randomization: comparison of allele score and summarized data methods. *Stat. Med.* **35**, 1880–1906. 10.1002/sim.6835 (2016). 26661904 10.1002/sim.6835 PMC4832315

[49] Bowden, J., Davey Smith, G., Haycock, P. C. & Burgess, S. Consistent Estimation in Mendelian randomization with some invalid instruments using a weighted median estimator. *Genet. Epidemiol.* **40**, 304–314. 10.1002/gepi.21965 (2016). 27061298 10.1002/gepi.21965 PMC4849733

[50] Lima Giacobbo, B. et al. Brain-Derived neurotrophic factor in brain disorders: focus on neuroinflammation. *Mol. Neurobiol.* **56**, 3295–3312. 10.1007/s12035-018-1283-6 (2019). 30117106 10.1007/s12035-018-1283-6 PMC6476855

[51] Azman, K. F. & Zakaria, R. Recent advances on the role of Brain-Derived neurotrophic factor (BDNF) in neurodegenerative diseases. *Int. J. Mol. Sci.* **23**10.3390/ijms23126827 (2022). 10.3390/ijms23126827 PMC9224343 35743271

[52] Laske, C. et al. BDNF serum and CSF concentrations in Alzheimer’s disease, normal pressure hydrocephalus and healthy controls. *J. Psychiatr Res.* **41**, 387–394. 10.1016/j.jpsychires.2006.01.014 (2007). 16554070 10.1016/j.jpsychires.2006.01.014

[53] Mori, Y. et al. Serum BDNF as a potential biomarker of Alzheimer’s disease: verification through assessment of serum, cerebrospinal fluid, and medial Temporal lobe atrophy. *Front. Neurol.* **12**, 653267. 10.3389/fneur.2021.653267 (2021). 33967943 10.3389/fneur.2021.653267 PMC8102980

[54] Sheldrick, A., Camara, S., Ilieva, M., Riederer, P. & Michel, T. M. Brain-derived neurotrophic factor (BDNF) and neurotrophin 3 (NT3) levels in post-mortem brain tissue from patients with depression compared to healthy individuals - a proof of concept study. *Eur. Psychiatry*. **46**, 65–71. 10.1016/j.eurpsy.2017.06.009 (2017). 29102768 10.1016/j.eurpsy.2017.06.009

[55] Pan, W., Banks, W. A., Fasold, M. B., Bluth, J. & Kastin, A. J. Transport of brain-derived neurotrophic factor across the blood-brain barrier. *Neuropharmacology* **37**, 1553–1561. 10.1016/s0028-3908(98)00141-5 (1998). 9886678 10.1016/s0028-3908(98)00141-5

[56] DiStefano, P. S. et al. The neurotrophins BDNF, NT-3, and NGF display distinct patterns of retrograde axonal transport in peripheral and central neurons. *Neuron* **8**, 983–993. 10.1016/0896-6273(92)90213-w (1992). 1375039 10.1016/0896-6273(92)90213-w

[57] Karege, F., Schwald, M. & Cisse, M. Postnatal developmental profile of brain-derived neurotrophic factor in rat brain and platelets. *Neurosci. Lett.* **328**, 261–264. 10.1016/s0304-3940(02)00529-3 (2002). 12147321 10.1016/s0304-3940(02)00529-3

[58] Adachi, N., Numakawa, T., Richards, M., Nakajima, S. & Kunugi, H. New insight in expression, transport, and secretion of brain-derived neurotrophic factor: implications in brain-related diseases. *World J. Biol. Chem.* **5**, 409–428. 10.4331/wjbc.v5.i4.409 (2014). 25426265 10.4331/wjbc.v5.i4.409 PMC4243146

[59] Zhang, X. Y. et al. Interaction of BDNF with cytokines in chronic schizophrenia. *Brain Behav. Immun.* **51**, 169–175. 10.1016/j.bbi.2015.09.014 (2016). 26407757 10.1016/j.bbi.2015.09.014

[60] Jeenger, J., Singroha, V., Sharma, M. & Mathur, D. M. C-reactive protein, brain-derived neurotrophic factor, interleukin-2, and stressful life events in drug-naive first-episode and recurrent depression: A cross-sectional study. *Indian J. Psychiatry*. **60**, 334–339. 10.4103/psychiatry.IndianJPsychiatry_169_18 (2018). 30405261 10.4103/psychiatry.IndianJPsychiatry_169_18 PMC6201676

[61] Segal, B. H. et al. Neutrophil interactions with T cells, platelets, endothelial cells, and of course tumor cells. *Immunol. Rev.* **314**, 13–35. 10.1111/imr.13178 (2023). 36527200 10.1111/imr.13178 PMC10174640

[62] Xu, L. et al. Elevated plasma BDNF levels are correlated with NK cell activation in patients with traumatic spinal cord injury. *Int. Immunopharmacol.* **74**, 105722. 10.1016/j.intimp.2019.105722 (2019). 31255880 10.1016/j.intimp.2019.105722

[63] Zhuang, X. et al. IL-33 in the basolateral amygdala integrates neuroinflammation into anxiogenic circuits via modulating BDNF expression. *Brain Behav. Immun.* **102**, 98–109. 10.1016/j.bbi.2022.02.019 (2022). 35181439 10.1016/j.bbi.2022.02.019

[64] Si, Z-Z. et al. Targeting neuroinflammation in Alzheimer’s disease: from mechanisms to clinical applications. *Neural Regeneration Res.* **18**, 708–715. 10.4103/1673-5374.353484 (2023). 10.4103/1673-5374.353484 PMC9700083 36204826

[65] Peng, S., Wuu, J., Mufson, E. J. & Fahnestock, M. Precursor form of brain-derived neurotrophic factor and mature brain-derived neurotrophic factor are decreased in the pre-clinical stages of Alzheimer’s disease. *J. Neurochem*. **93**, 1412–1421. 10.1111/j.1471-4159.2005.03135.x (2005). 15935057 10.1111/j.1471-4159.2005.03135.x

[66] Holsinger, R. M., Schnarr, J., Henry, P., Castelo, V. T. & Fahnestock, M. Quantitation of BDNF mRNA in human parietal cortex by competitive reverse transcription-polymerase chain reaction: decreased levels in Alzheimer’s disease. *Brain Res. Mol. Brain Res.* **76**, 347–354. 10.1016/s0169-328x(00)00023-1 (2000). 10762711 10.1016/s0169-328x(00)00023-1

[67] Fu, A. K. Y. et al. IL-33 ameliorates Alzheimer’s disease-like pathology and cognitive decline. *Proceedings of the National Academy of Sciences*. ;113:E2705-E2713. doi: (2016). 10.1073/pnas.1604032113 10.1073/pnas.1604032113 PMC4868478 27091974

[68] Lau, S. F. et al. IL-33-PU.1 transcriptome reprogramming drives functional state transition and clearance activity of microglia in Alzheimer’s disease. *Cell. Rep.* **31**, 107530. 10.1016/j.celrep.2020.107530 (2020). 32320664 10.1016/j.celrep.2020.107530

[69] Liang, C-S. et al. The role of interleukin-33 in patients with mild cognitive impairment and Alzheimer’s disease. *Alzheimers Res. Ther.* **12**, 86. 10.1186/s13195-020-00652-z (2020). 32678011 10.1186/s13195-020-00652-z PMC7367330

[70] Martino, M. et al. NGF serum levels variations in major depressed patients receiving Duloxetine. *Psychoneuroendocrinology* **38**, 1824–1828. 10.1016/j.psyneuen.2013.02.009 (2013). 23507186 10.1016/j.psyneuen.2013.02.009

[71] Wysokiński, A. Serum levels of brain-derived neurotrophic factor (BDNF) and neurotrophin-3 (NT-3) in depressed patients with schizophrenia. *Nord J. Psychiatry*. **70**, 267–271. 10.3109/08039488.2015.1087592 (2016). 26548545 10.3109/08039488.2015.1087592

[72] Smith, M. A., Makino, S., Kvetnansky, R. & Post, R. M. Stress and glucocorticoids affect the expression of brain-derived neurotrophic factor and neurotrophin-3 mRNAs in the hippocampus. *J. Neurosci.* **15**, 1768–1777. 10.1523/jneurosci.15-03-01768.1995 (1995). 7891134 10.1523/JNEUROSCI.15-03-01768.1995 PMC6578156

[73] Stanne, T. M. et al. Low Circulating acute Brain-Derived neurotrophic factor levels are associated with poor Long-Term functional outcome after ischemic stroke. *Stroke* **47**, 1943–1945. 10.1161/strokeaha.115.012383 (2016). 27301948 10.1161/STROKEAHA.115.012383

[74] Pascotini, E. T. et al. Apoptotic markers and DNA damage are related to late phase of stroke: involvement of dyslipidemia and inflammation. *Physiol. Behav.* **151**, 369–378. 10.1016/j.physbeh.2015.08.005 (2015). 26253215 10.1016/j.physbeh.2015.08.005

[75] Schluns, K. S., Stoklasek, T. & Lefrançois, L. The roles of interleukin-15 receptor alpha: trans-presentation, receptor component, or both? *Int. J. Biochem. Cell. Biol.* **37**, 1567–1571. 10.1016/j.biocel.2005.02.017 (2005). 15896666 10.1016/j.biocel.2005.02.017

[76] Pan, W. et al. Brain interleukin-15 in neuroinflammation and behavior. *Neurosci. Biobehav Rev.* **37**, 184–192. 10.1016/j.neubiorev.2012.11.009 (2013). 23201098 10.1016/j.neubiorev.2012.11.009 PMC3563733

[77] Beck, R. D. Jr. et al. Changes in hippocampal IL-15, related cytokines, and neurogenesis in IL-2 deficient mice. *Brain Res.* **1041**, 223–230. 10.1016/j.brainres.2005.02.010 (2005). 15829231 10.1016/j.brainres.2005.02.010

[78] Chang, S. H. et al. Interleukin-17 C promotes Th17 cell responses and autoimmune disease via interleukin-17 receptor E. *Immunity* **35**, 611–621. 10.1016/j.immuni.2011.09.010 (2011). 21982598 10.1016/j.immuni.2011.09.010 PMC5800502

[79] Olcott, C. M., Han, J. K., Cunningham, T. D. & Franzese, C. B. Interleukin-9 and interleukin-17 C in chronic rhinosinusitis. *Int. Forum Allergy Rhinol*. **6**, 841–847. 10.1002/alr.21745 (2016). 26989880 10.1002/alr.21745

[80] Waisman, A., Hauptmann, J. & Regen, T. The role of IL-17 in CNS diseases. *Acta Neuropathol.* **129**, 625–637. 10.1007/s00401-015-1402-7 (2015). 25716179 10.1007/s00401-015-1402-7

[81] Chen, W. Y. & Chang, M. S. IL-20 is regulated by hypoxia-inducible factor and up-regulated after experimental ischemic stroke. *J. Immunol.* **182**, 5003–5012. 10.4049/jimmunol.0803653 (2009). 19342680 10.4049/jimmunol.0803653

[82] Janssens, K. et al. Leukemia inhibitory factor tips the immune balance towards regulatory T cells in multiple sclerosis. *Brain Behav. Immun.* **45**, 180–188. 10.1016/j.bbi.2014.11.010 (2015). 25514345 10.1016/j.bbi.2014.11.010

[83] Widenfalk, J. et al. Neurturin and glial cell line-derived neurotrophic factor receptor-beta (GDNFR-beta), novel proteins related to GDNF and GDNFR-alpha with specific cellular patterns of expression suggesting roles in the developing and adult nervous system and in peripheral organs. *J. Neurosci.* **17**, 8506–8519. 10.1523/jneurosci.17-21-08506.1997 (1997). 9334423 10.1523/JNEUROSCI.17-21-08506.1997 PMC6573771

[84] Horger, B. A. et al. Neurturin exerts potent actions on survival and function of midbrain dopaminergic neurons. *J. Neurosci.* **18**, 4929–4937. 10.1523/jneurosci.18-13-04929.1998 (1998). 9634558 10.1523/JNEUROSCI.18-13-04929.1998 PMC6792569

[85] Faura, J. et al. CCL23: A chemokine associated with progression from mild cognitive impairment to Alzheimer’s disease. *J. Alzheimers Dis.* **73**, 1585–1595. 10.3233/jad-190753 (2020). 31958084 10.3233/JAD-190753 PMC8010612

[86] Simats, A. et al. CCL23: a new CC chemokine involved in human brain damage. *J. Intern. Med.* **283**, 461–475. 10.1111/joim.12738 (2018). 29415332 10.1111/joim.12738

[87] Bonaventura, A. & Montecucco, F. CCL23 is a promising biomarker of injury in patients with ischaemic stroke. *J. Intern. Med.* **283**, 476–478. 10.1111/joim.12742 (2018). 29443424 10.1111/joim.12742

[88] Chen, J. et al. Peripheral inflammatory biomarkers are associated with cognitive function and dementia: Framingham heart study offspring cohort. *Aging Cell.* **22**, e13955. 10.1111/acel.13955 (2023). 37584418 10.1111/acel.13955 PMC10577533

[89] Zhang, X. et al. The involvement of ADAR1 in antidepressant action by regulating BDNF via miR-432. *Behav. Brain Res.* **402**, 113087. 10.1016/j.bbr.2020.113087 (2021). 33412228 10.1016/j.bbr.2020.113087

[90] Elgün, S., Keskinege, A. & Kumbasar, H. Dipeptidyl peptidase IV and adenosine deaminase activity. Decrease in depression. *Psychoneuroendocrinology* **24**, 823–832. 10.1016/s0306-4530(99)00039-6 (1999). 10581653 10.1016/s0306-4530(99)00039-6

[91] Hill, L. J. et al. Cystatin D (CST5): an ultra-early inflammatory biomarker of traumatic brain injury. *Sci. Rep.* **7**, 5002. 10.1038/s41598-017-04722-5 (2017). 28694499 10.1038/s41598-017-04722-5 PMC5504020

[92] Louissaint, A. Jr., Rao, S., Leventhal, C. & Goldman, S. A. Coordinated interaction of neurogenesis and angiogenesis in the adult Songbird brain. *Neuron* **34**, 945–960. 10.1016/s0896-6273(02)00722-5 (2002). 12086642 10.1016/s0896-6273(02)00722-5

[93] Li, X. et al. The prognostic value of serum cytokines in patients with acute ischemic stroke. *Aging Dis.* **10**, 544–556. 10.14336/ad.2018.0820 (2019). 31164999 10.14336/AD.2018.0820 PMC6538221

[94] Ng, A. et al. IL-6, TNF- α and CRP in elderly patients with depression or Alzheimer’s disease: systematic review and Meta-Analysis. *Sci. Rep.* **8**, 12050. 10.1038/s41598-018-30487-6 (2018). 30104698 10.1038/s41598-018-30487-6 PMC6089986

[95] Soltani Khaboushan, A. et al. Circulating levels of inflammatory biomarkers in Huntington’s disease: A systematic review and meta-analysis. *J. Neuroimmunol.* **385**, 578243. 10.1016/j.jneuroim.2023.578243 (2023). 37984118 10.1016/j.jneuroim.2023.578243

[96] Bonotis, K. et al. Systemic immune aberrations in Alzheimer’s disease patients. *J. Neuroimmunol.* **193**, 183–187. 10.1016/j.jneuroim.2007.10.020 (2008). 18037502 10.1016/j.jneuroim.2007.10.020

[97] Licastro F, Pedrini S, Caputo L, Annoni G, Davis LJ, Ferri C, Casadei V, Grimaldi LME. Increased plasma levels of interleukin-1, interleukin-6 and α-1-antichymotrypsin in patients with Alzheimer’s disease: peripheral inflammation or signals from the brain? *Journal of Neuroimmunology*. 2000;103:97–102. doi: 10.1016/S0165-5728(99)00226-X. 10.1016/s0165-5728(99)00226-x 10674995

[98] Huang, C-W. et al. Potential blood biomarker for disease severity in the Taiwanese population with Alzheimer’s disease. *Am. J. Alzheimer’s Disease Other Dementias*^®^. **28**, 75–83. 10.1177/1533317512467674 (2013). 10.1177/1533317512467674 PMC10697223 23230229

[99] Lu, G., Liu, W., Huang, X. & Zhao, Y. Complement factor H levels are decreased and correlated with serum C-reactive protein in late-onset Alzheimer’s disease. *Arq. Neuropsiquiatr.* **78**, 76–80. 10.1590/0004-282x20190151 (2020). 32022122 10.1590/0004-282X20190151

[100] Durany, N. et al. Brain-derived neurotrophic factor and neurotrophin-3 levels in Alzheimer’s disease brains. *Int. J. Dev. Neurosci.* **18**, 807–813 (2000). 11154850

[101] Murer, M. G. et al. An immunohistochemical study of the distribution of brain-derived neurotrophic factor in the adult human brain, with particular reference to Alzheimer’s disease. *Neuroscience* **88**, 1015–1032. 10.1016/s0306-4522(98)00219-x (1999). 10336117 10.1016/s0306-4522(98)00219-x

[102] Trajkovska, V. et al. Measurements of brain-derived neurotrophic factor: methodological aspects and demographical data. *Brain Res. Bull.* **73**, 143–149. 10.1016/j.brainresbull.2007.03.009 (2007). 17499648 10.1016/j.brainresbull.2007.03.009

[103] Eide, S., Misztal, M. & Feng, Z-P. Interleukin-6 as a marker of Huntington’s disease progression: systematic review and meta-analysis. *Brain Behav. Immun. - Health*. **30**, 100635. 10.1016/j.bbih.2023.100635 (2023). 37215308 10.1016/j.bbih.2023.100635 PMC10196779

[104] Yap, N. Y., Toh, Y. L., Tan, C. J., Acharya, M. M. & Chan, A. Relationship between cytokines and brain-derived neurotrophic factor (BDNF) in trajectories of cancer-related cognitive impairment. *Cytokine* **144**, 155556. 10.1016/j.cyto.2021.155556 (2021). 33985854 10.1016/j.cyto.2021.155556 PMC8585614

[105] Wertz, M. H. et al. Interleukin-6 deficiency exacerbates Huntington’s disease model phenotypes. *Mol. Neurodegeneration*. **15**, 29. 10.1186/s13024-020-00379-3 (2020). 10.1186/s13024-020-00379-3 PMC7247164 32448329

[106] Wang, R. et al. Metabolic and hormonal signatures in pre-manifest and manifest Huntington’s disease patients. *Front. Physiol.* **5**10.3389/fphys.2014.00231 (2014). 10.3389/fphys.2014.00231 PMC4066441 25002850

[107] Silajdžić, E. et al. A critical evaluation of inflammatory markers in Huntington’s disease plasma. *J. Huntington’s Disease*. **2**, 125–134. 10.3233/JHD-130049 (2013). 25063434 10.3233/JHD-130049

