# Supplementary material for: Deciphering genetic causality between plasma BDNF and 91 circulating inflammatory proteins through bidirectional mendelian randomization
Source: Sci Rep. 2025 Mar 25;15:10312. doi: 10.1038/s41598-025-95546-1 (PMC11937598; doi:10.1038/s41598-025-95546-1)

Figure S1. Forest plot of the causal effect of BDNF on Beta-NGF.


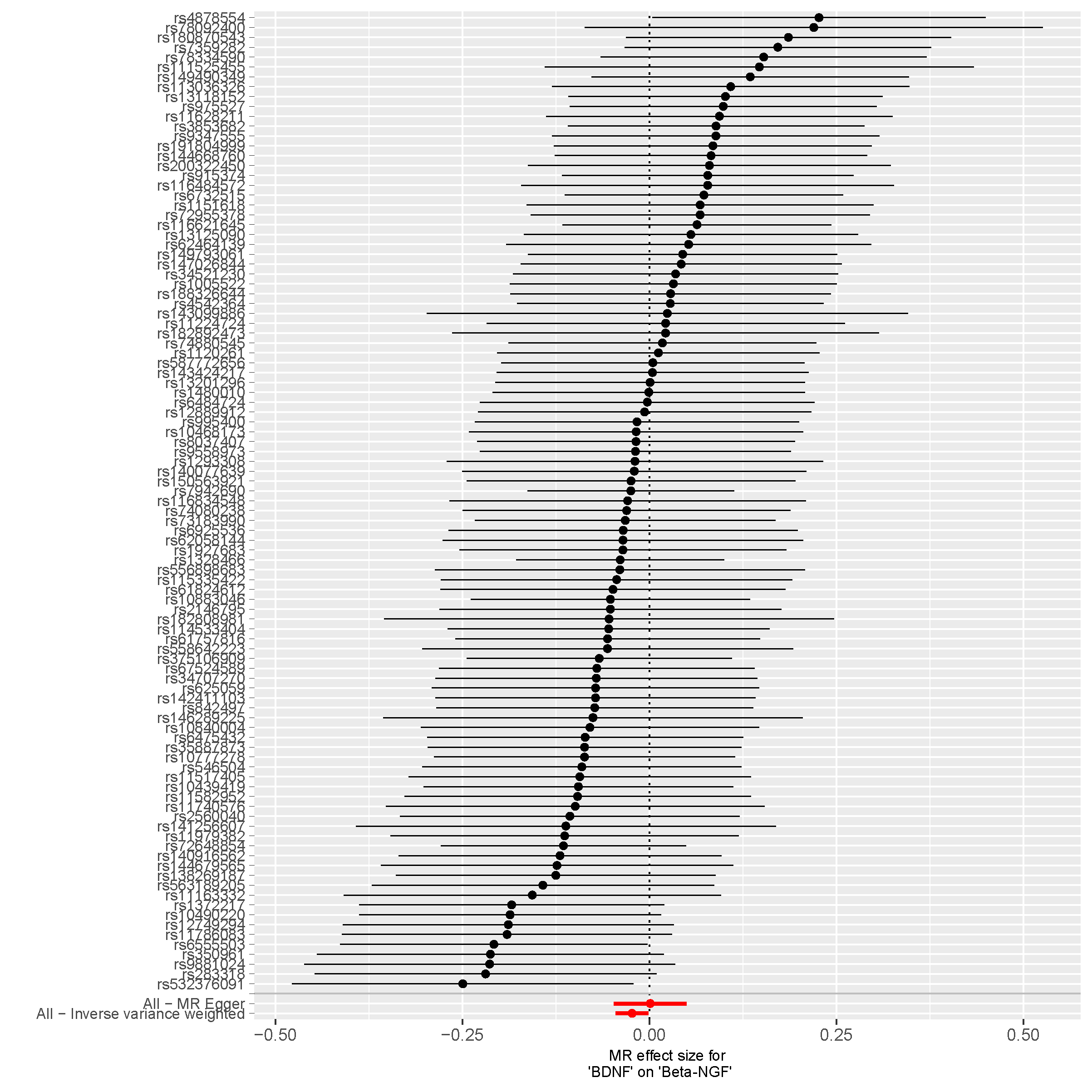


Figure S2. Forest plot of the causal effect of BDNF on CASP-8.


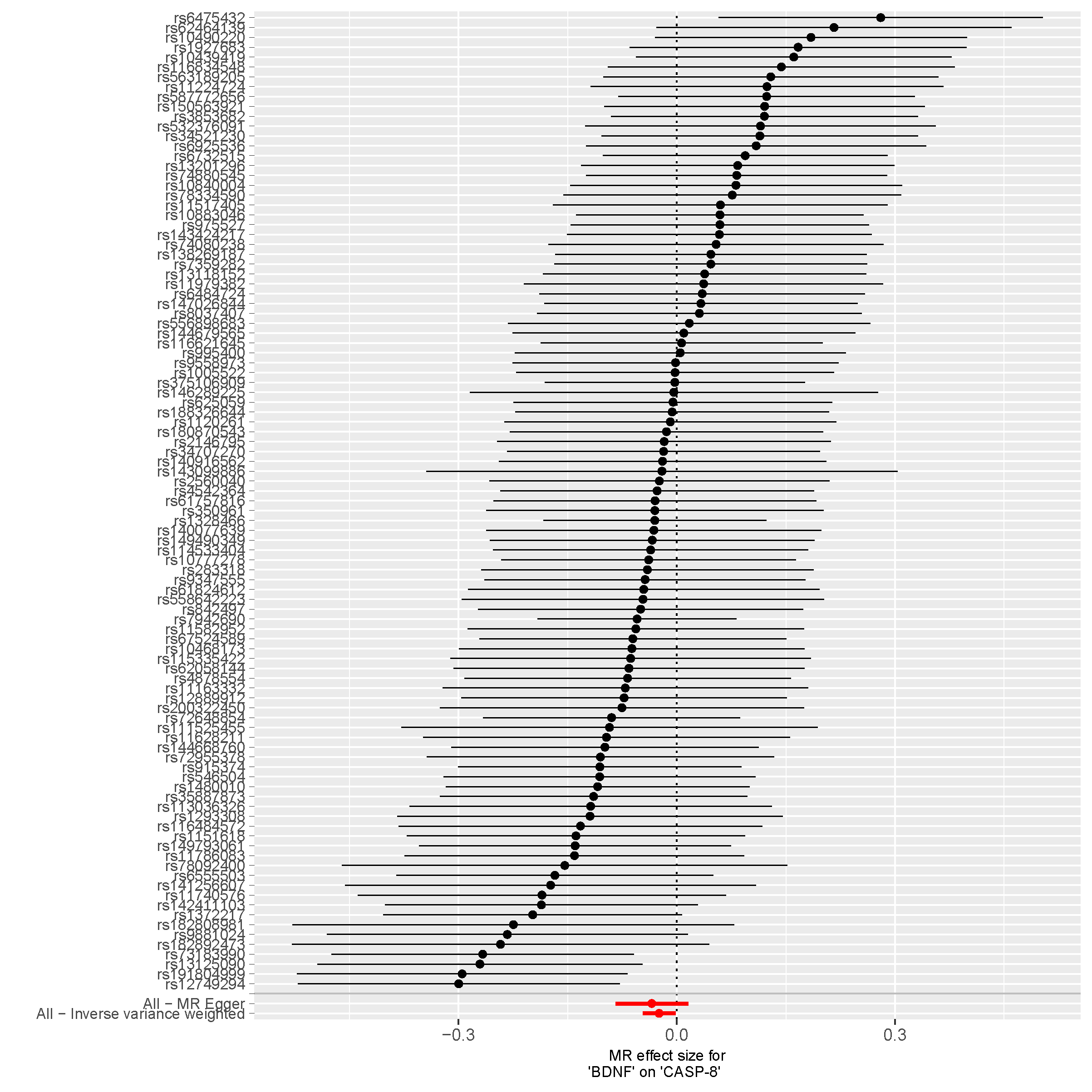


Figure S3. Forest plot of the causal effect of BDNF on IL-15RA.


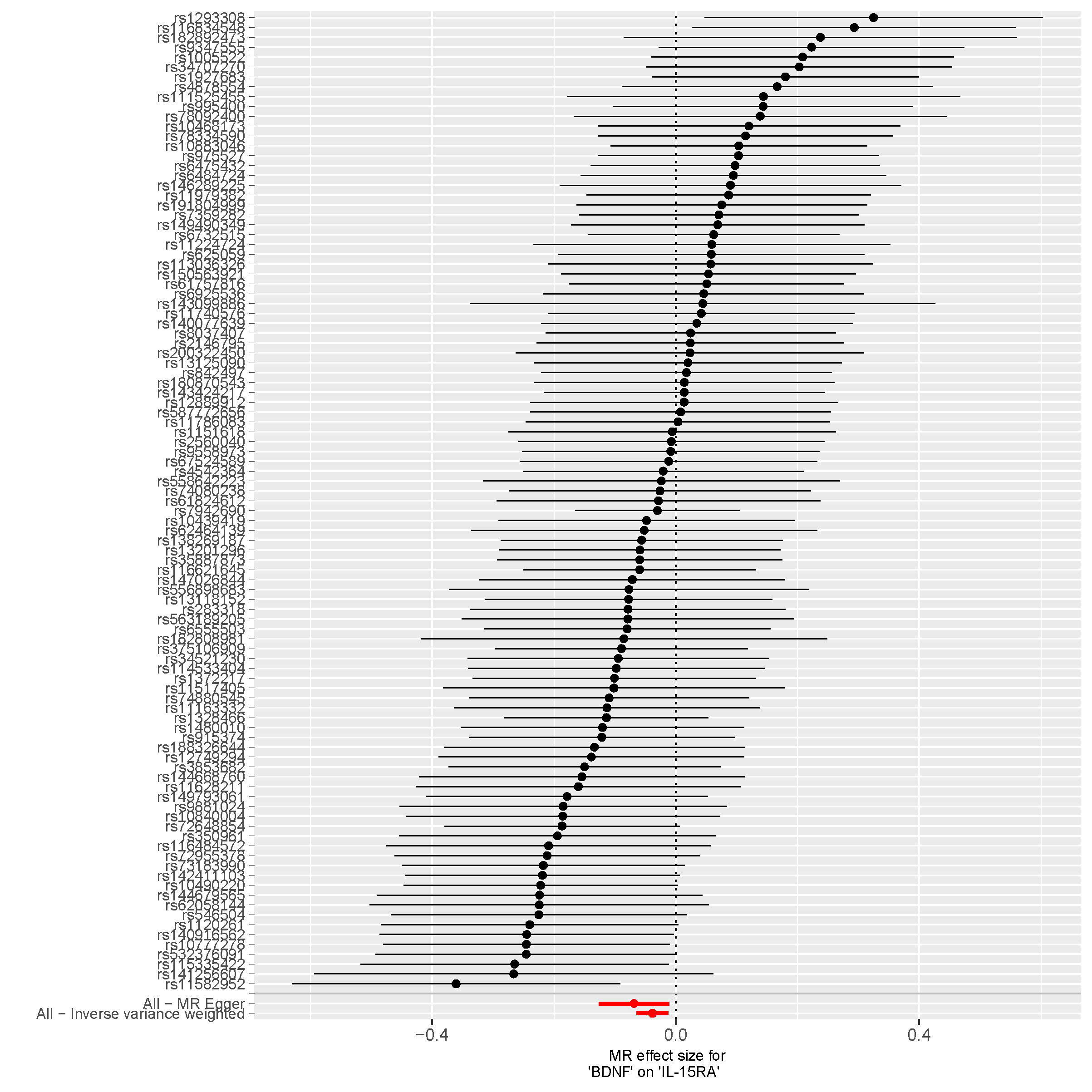


Figure S4. Forest plot of the causal effect of BDNF on IL-17A.


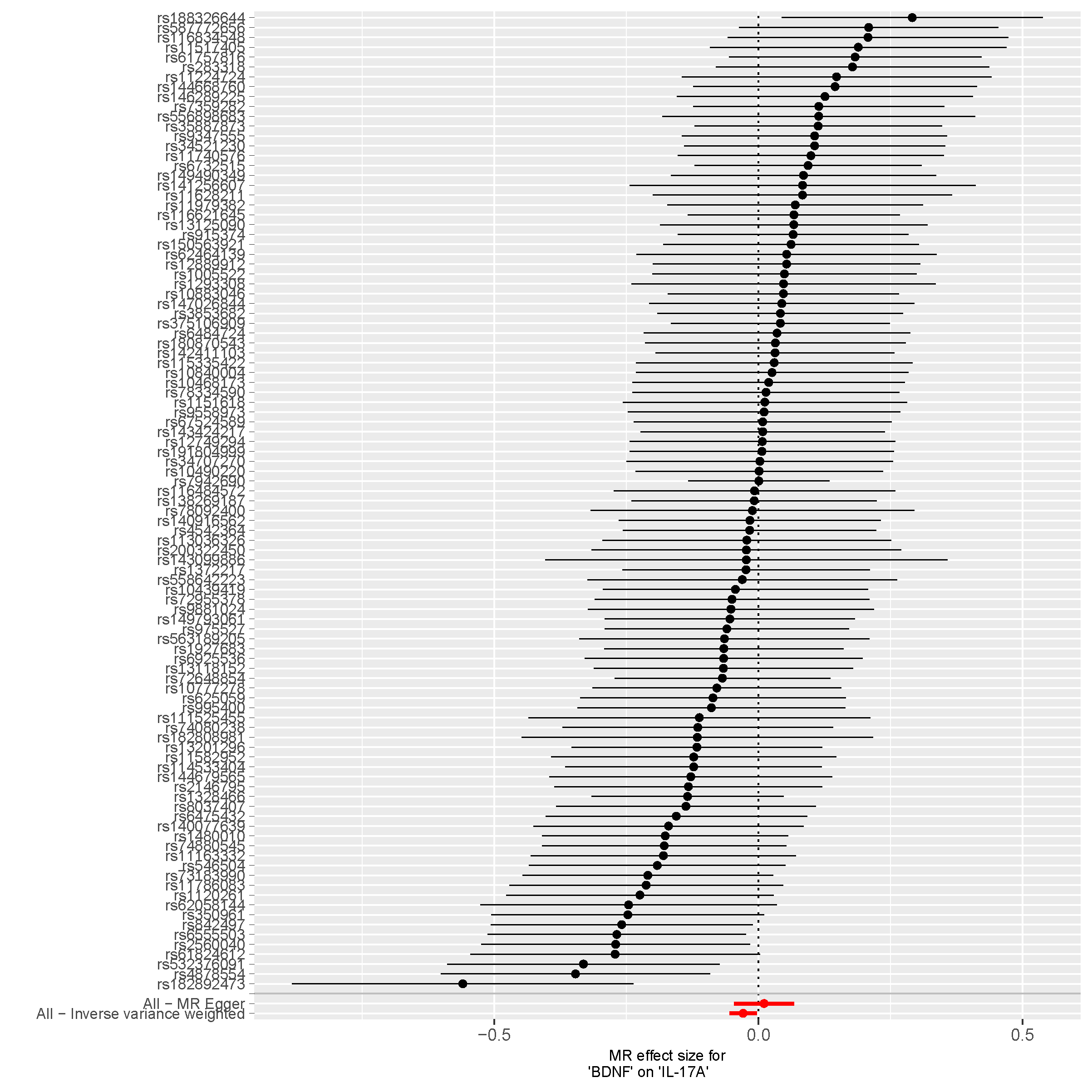


Figure S5. Forest plot of the causal effect of BDNF on IL-17C.


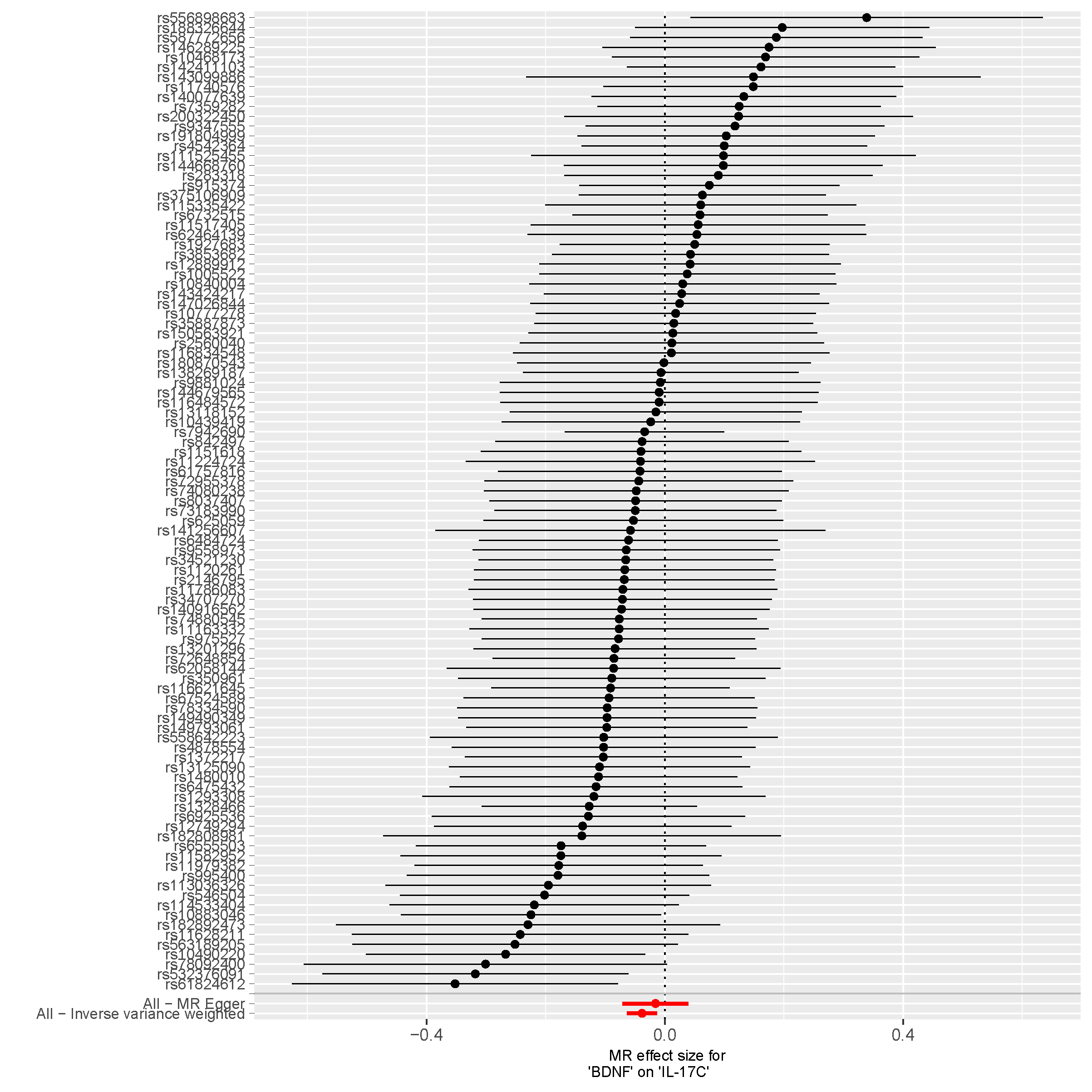


Figure S6. Forest plot of the causal effect of BDNF on IL-2.


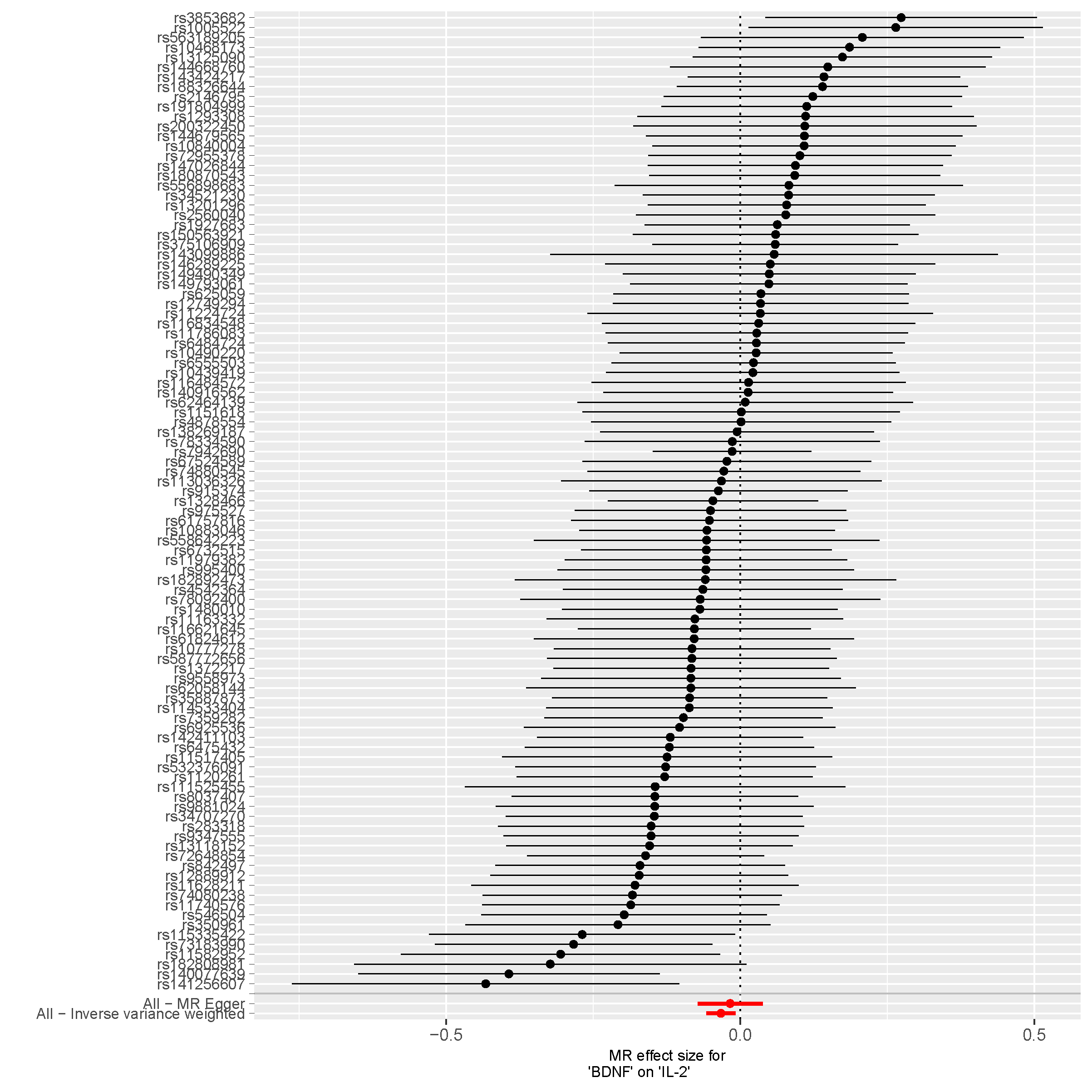


Figure S7. Forest plot of the causal effect of BDNF on IL-20.


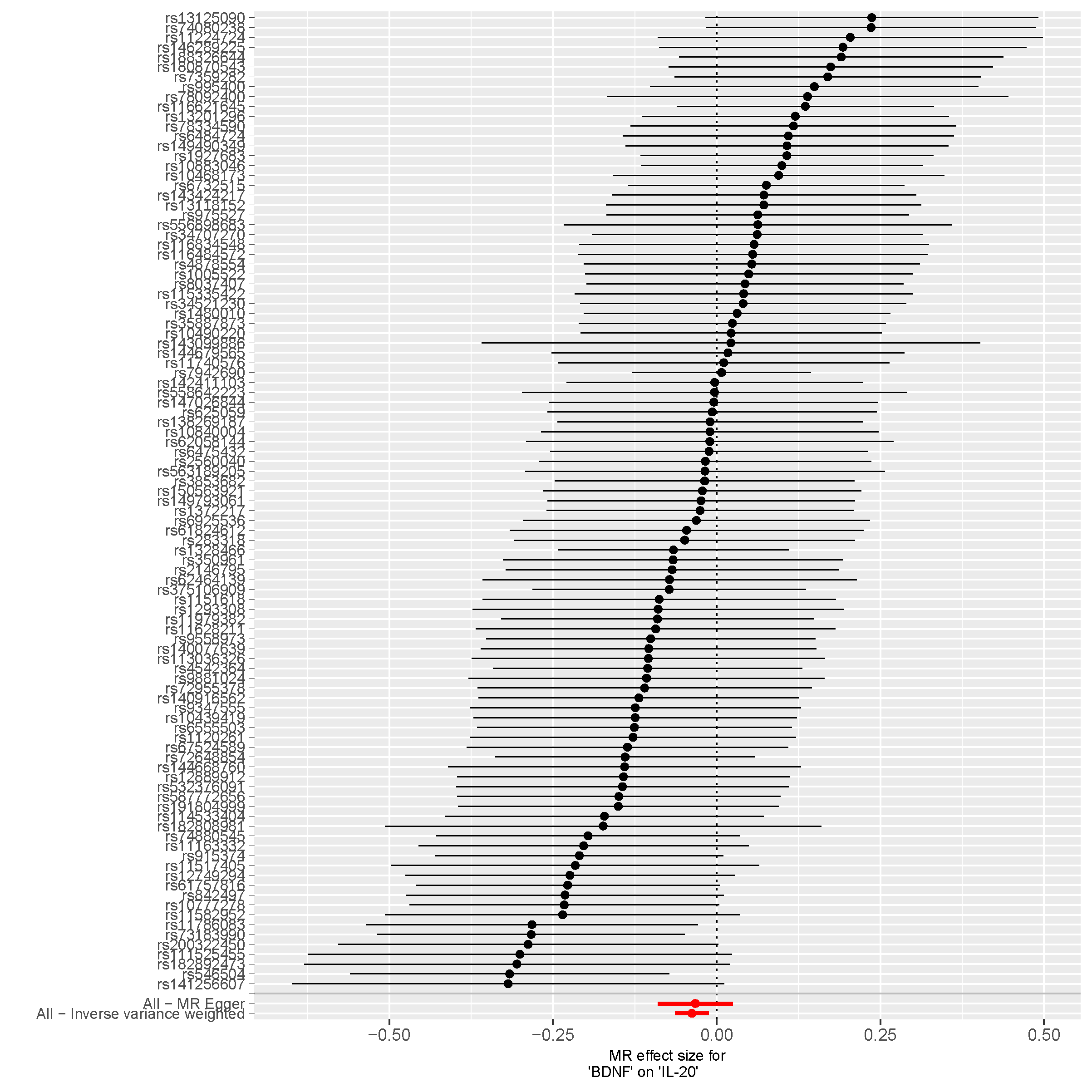


Figure S8. Forest plot of the causal effect of BDNF on IL-20RA.


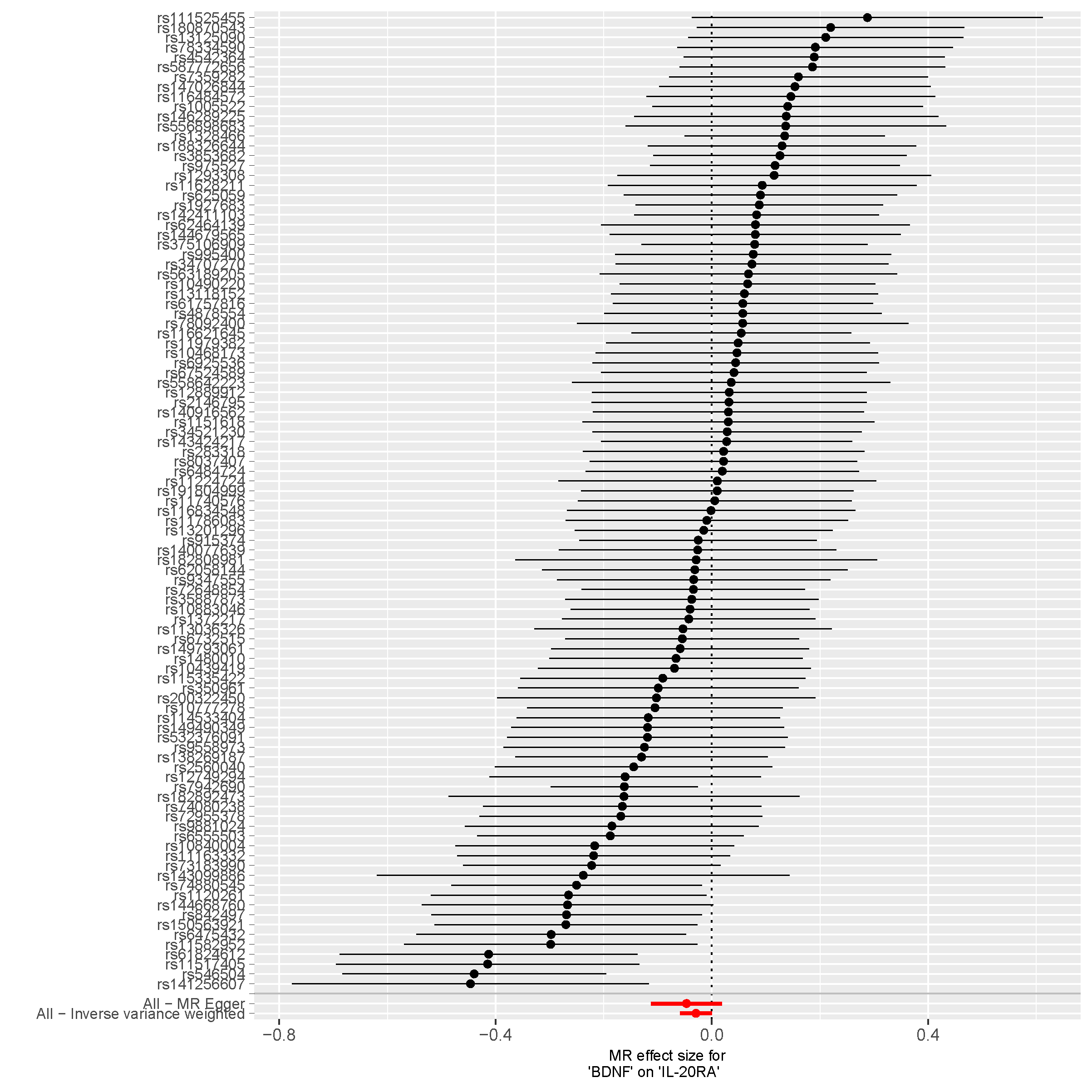


Figure S9. Forest plot of the causal effect of BDNF on IL-24.


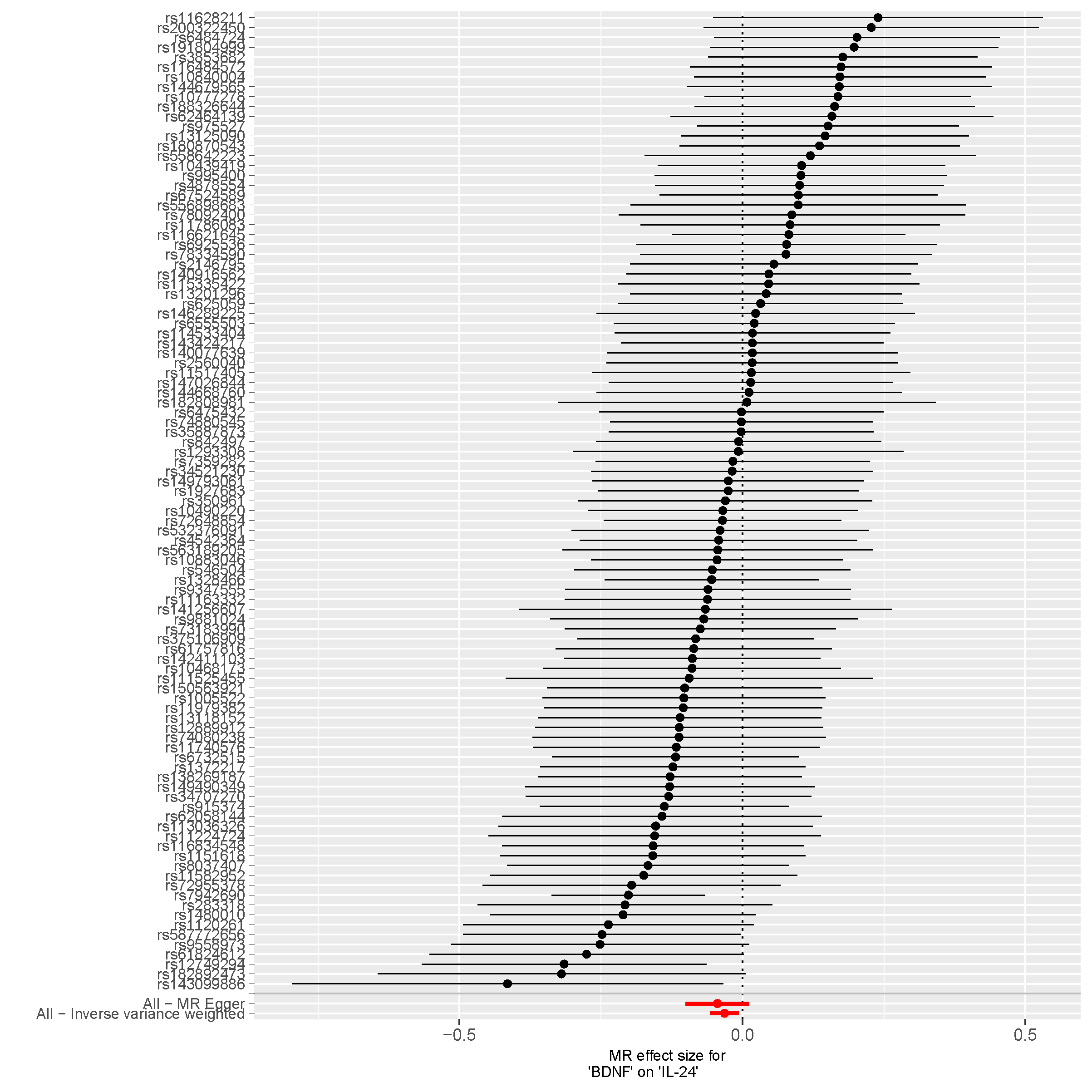


Figure S10. Forest plot of the causal effect of BDNF on IL-33.


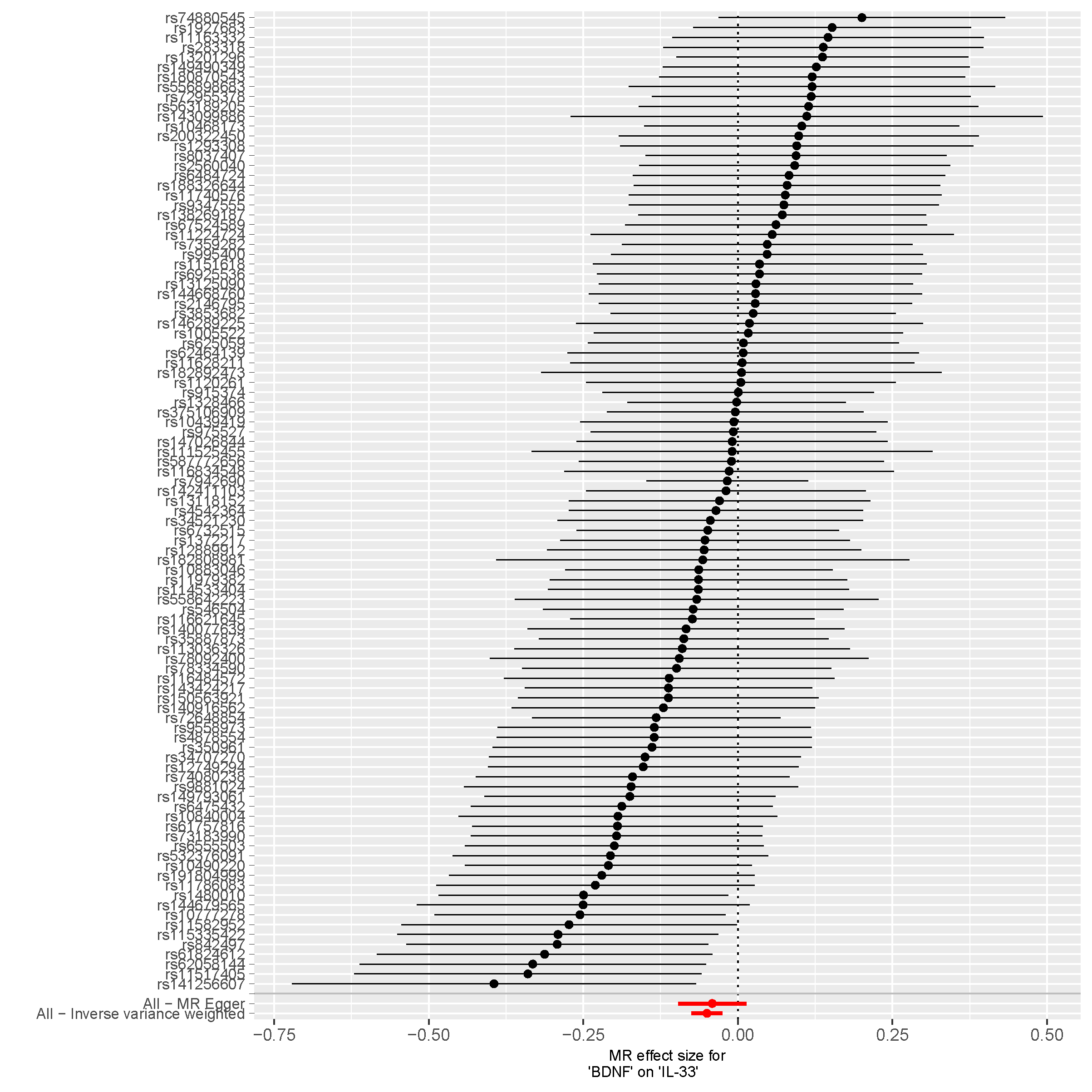


Figure S11. Forest plot of the causal effect of BDNF on LIF.


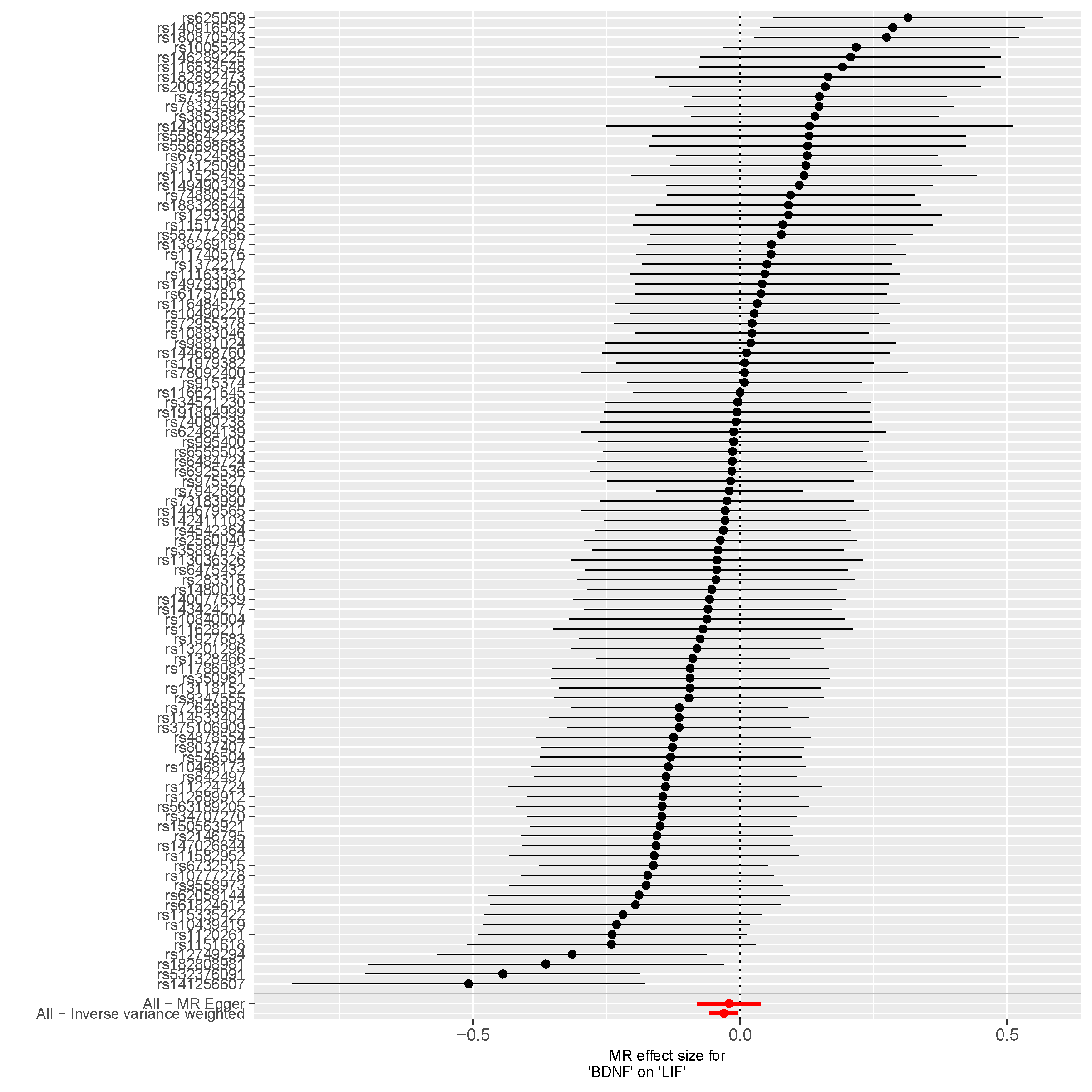


Figure S12. Forest plot of the causal effect of BDNF on NRTN.


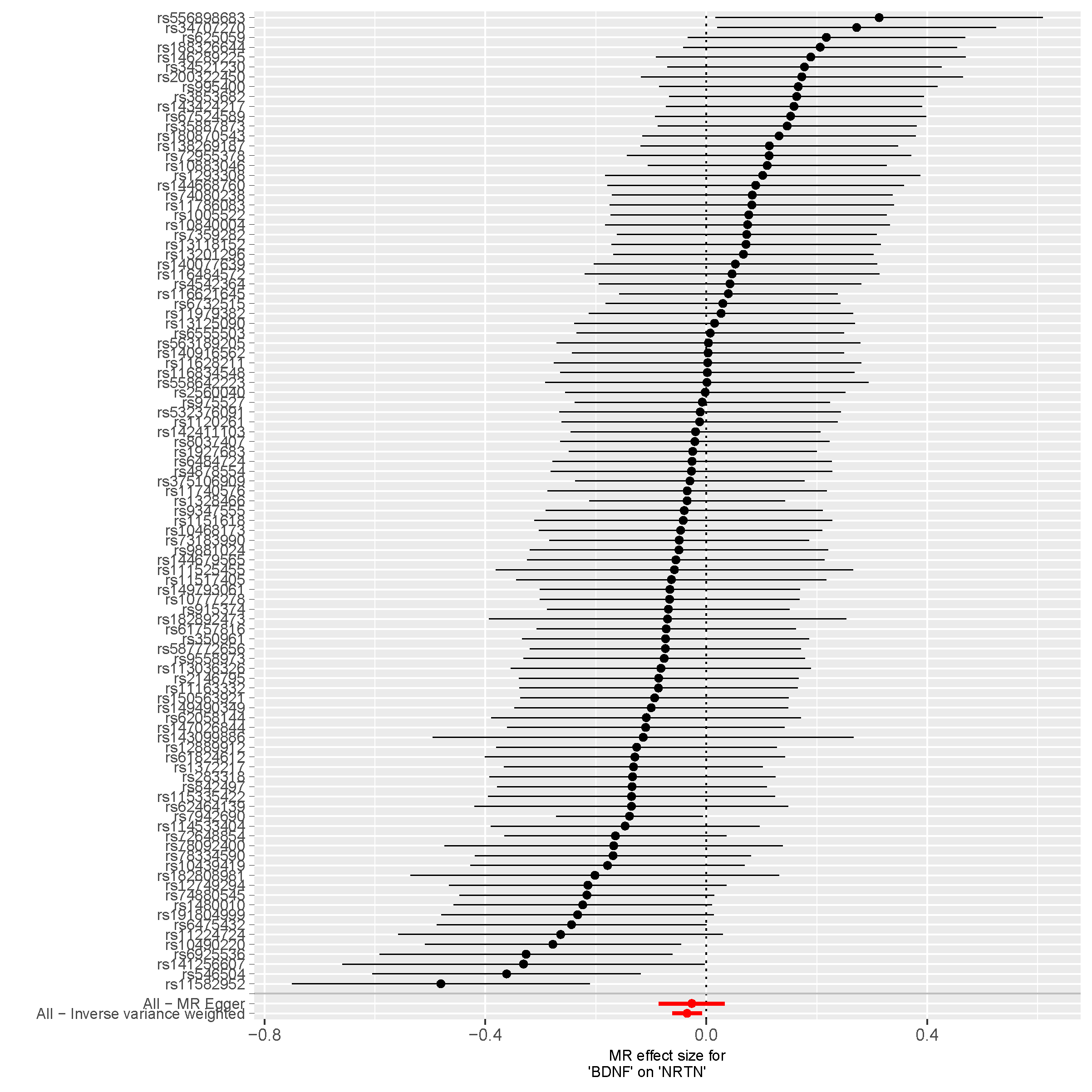


Figure S13. Forest plot of the causal effect of BDNF on NT-3.


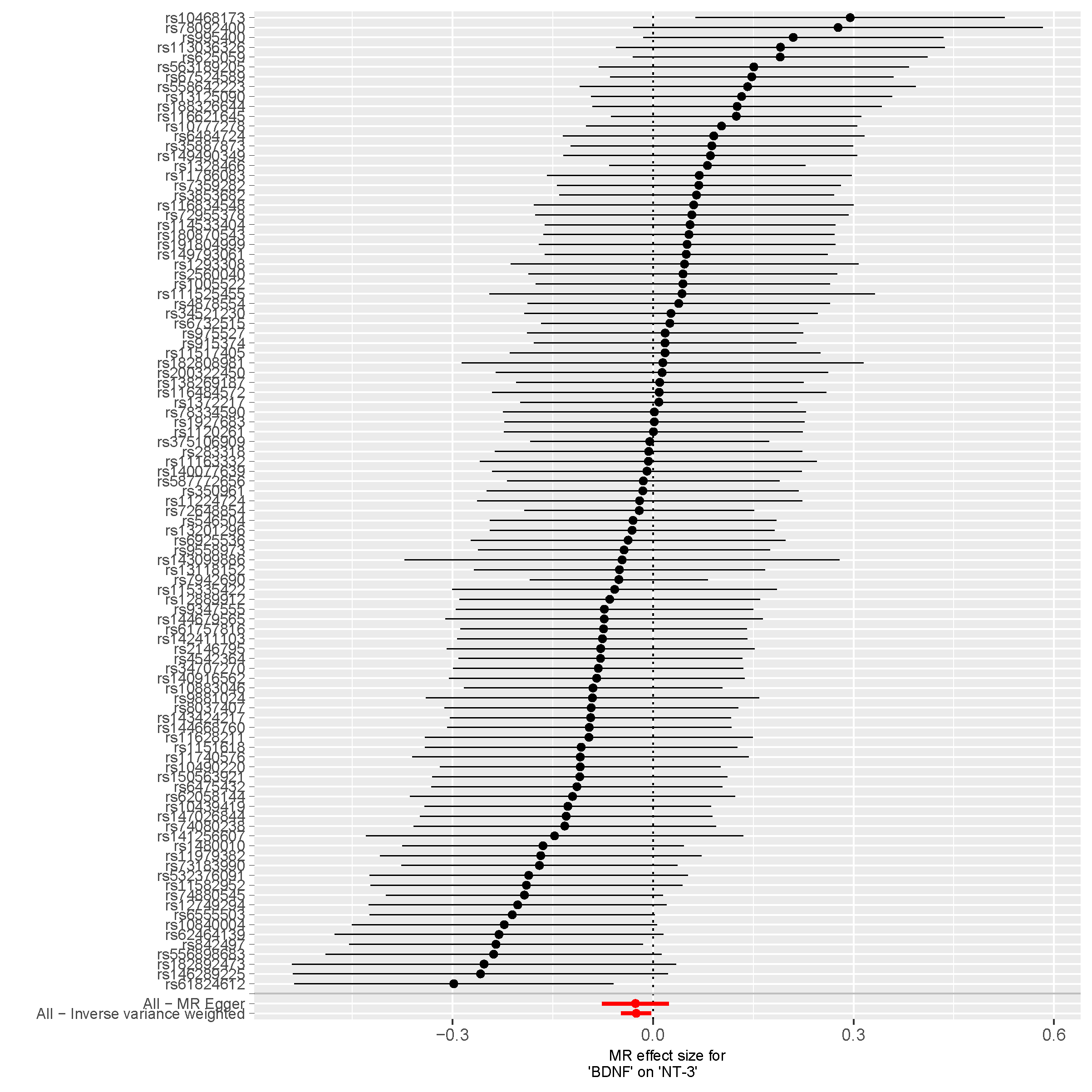


Figure S14. Leave-one-out analysis for the causal effect of BDNF on Beta-NGF.
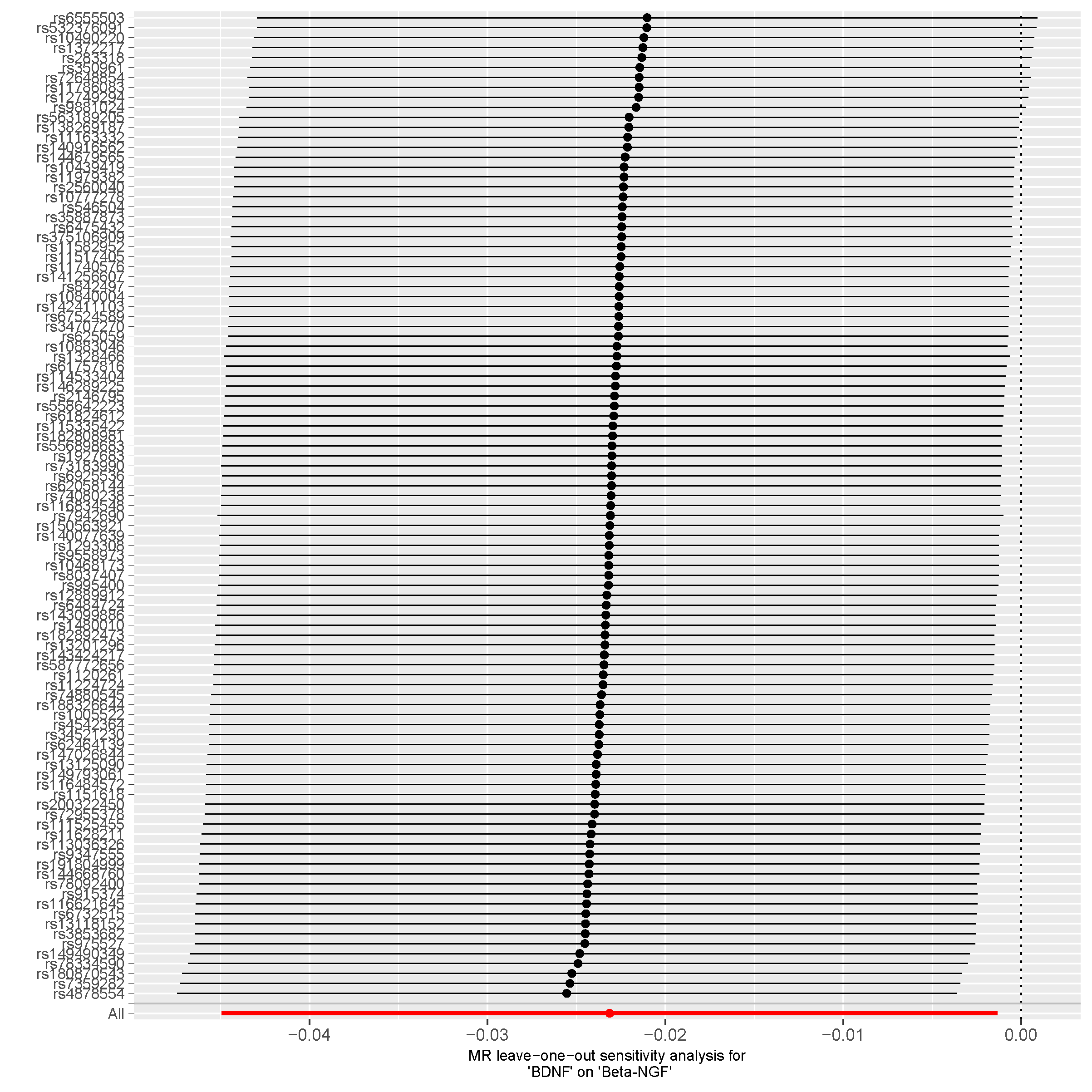


Figure S15. Leave-one-out analysis for the causal effect of BDNF on CASP-8.
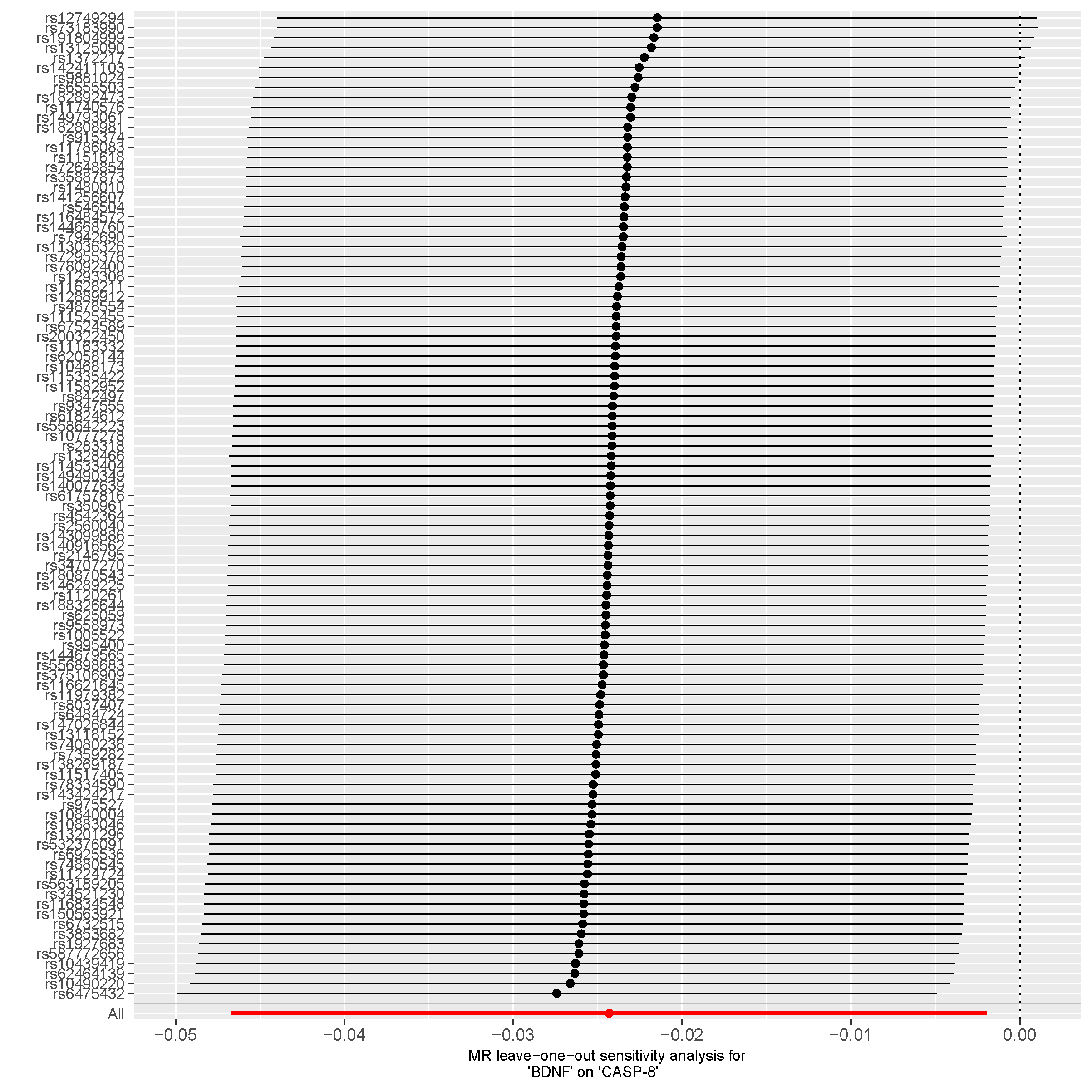


Figure S16. Leave-one-out analysis for the causal effect of BDNF on IL-15RA.
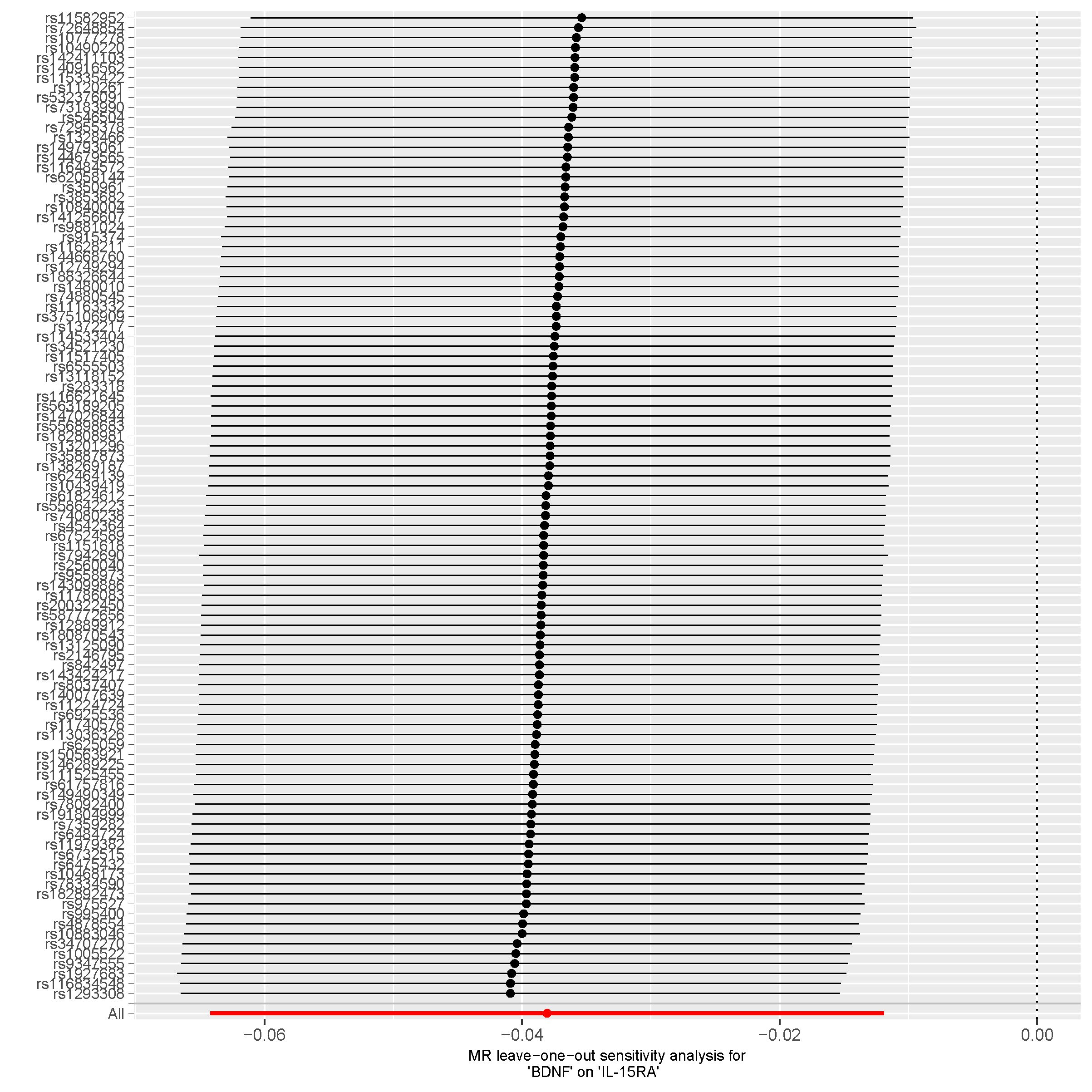


Figure S17. Leave-one-out analysis for the causal effect of BDNF on IL-17A.
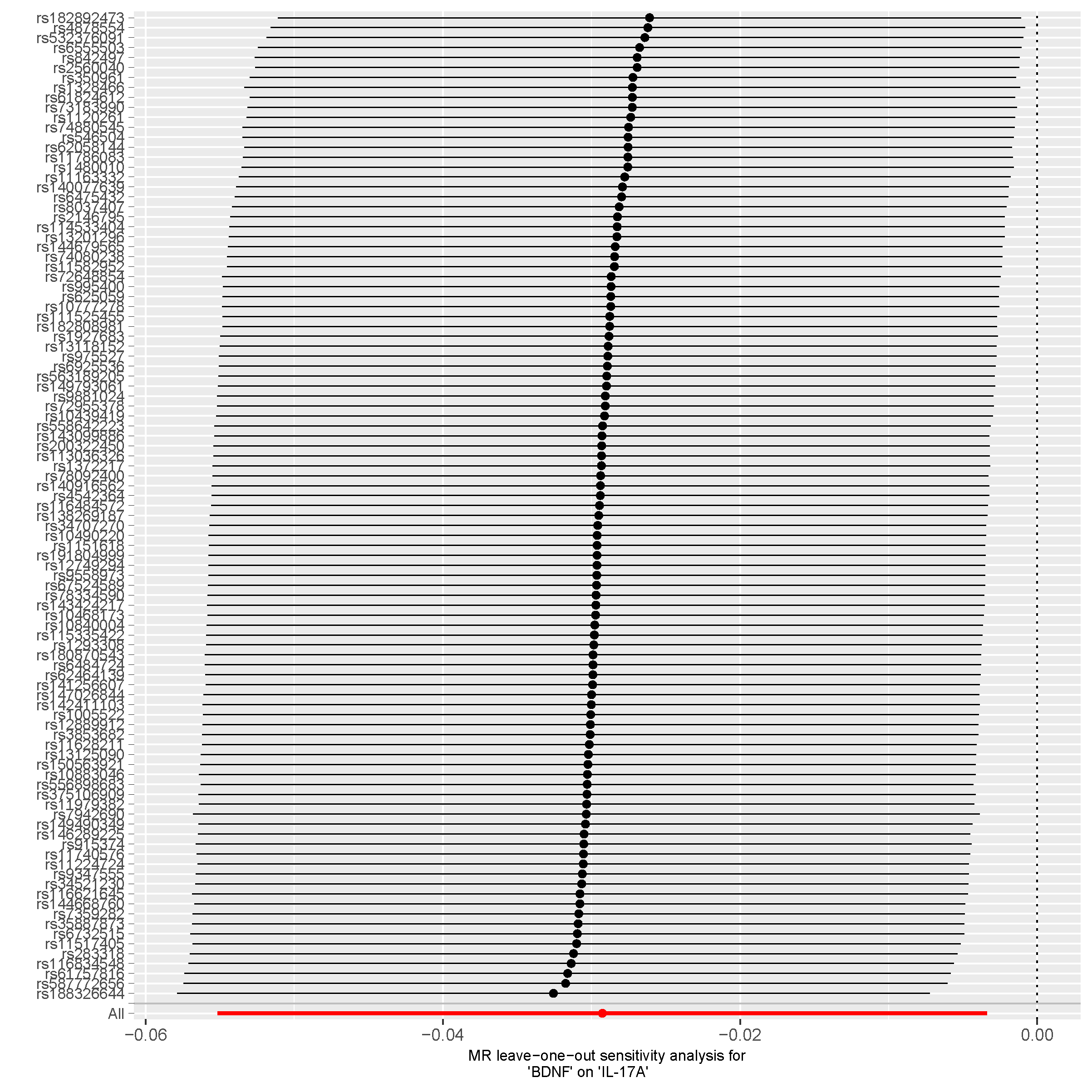


Figure S18. Leave-one-out analysis for the causal effect of BDNF on IL-17C.
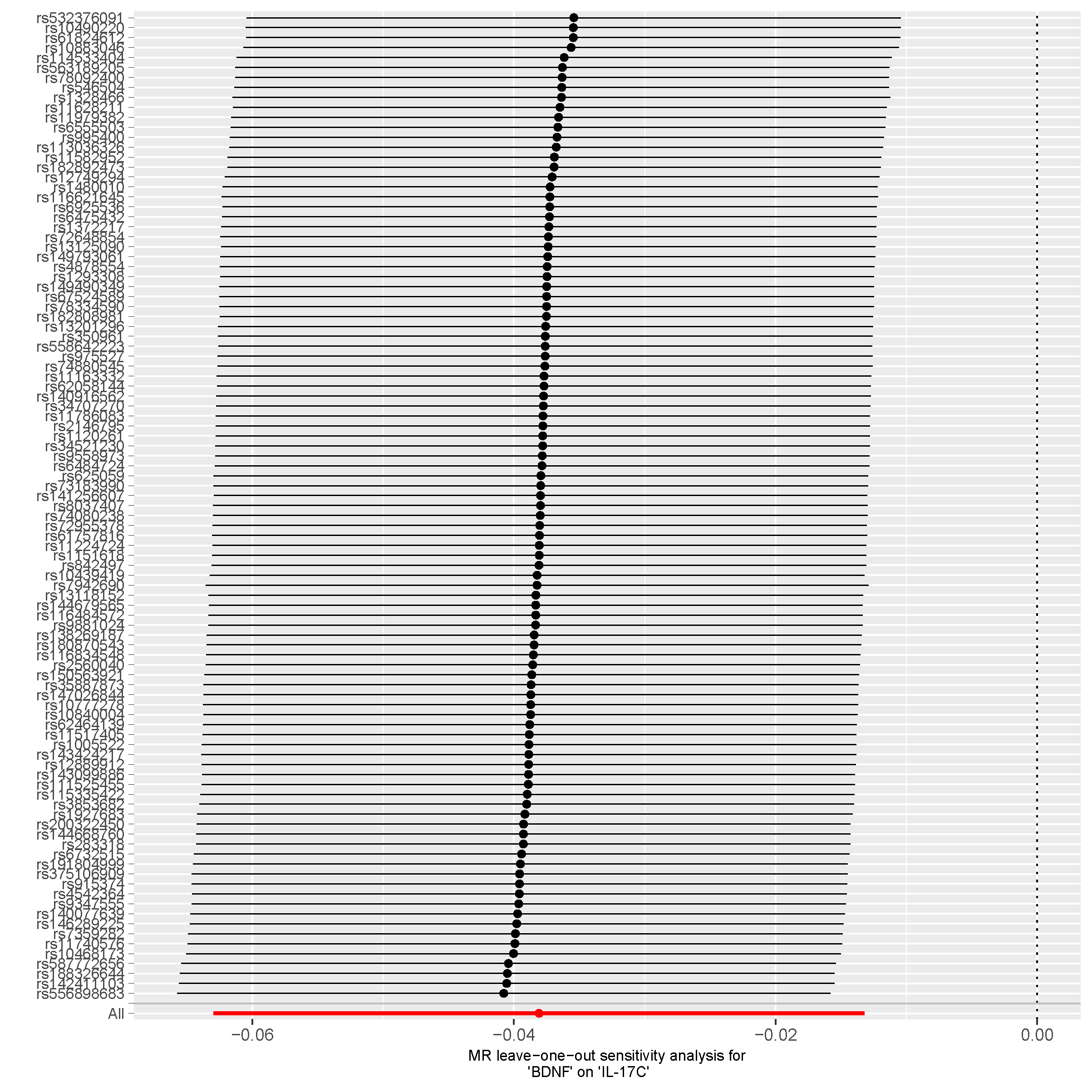


Figure S19. Leave-one-out analysis for the causal effect of BDNF on IL-2.
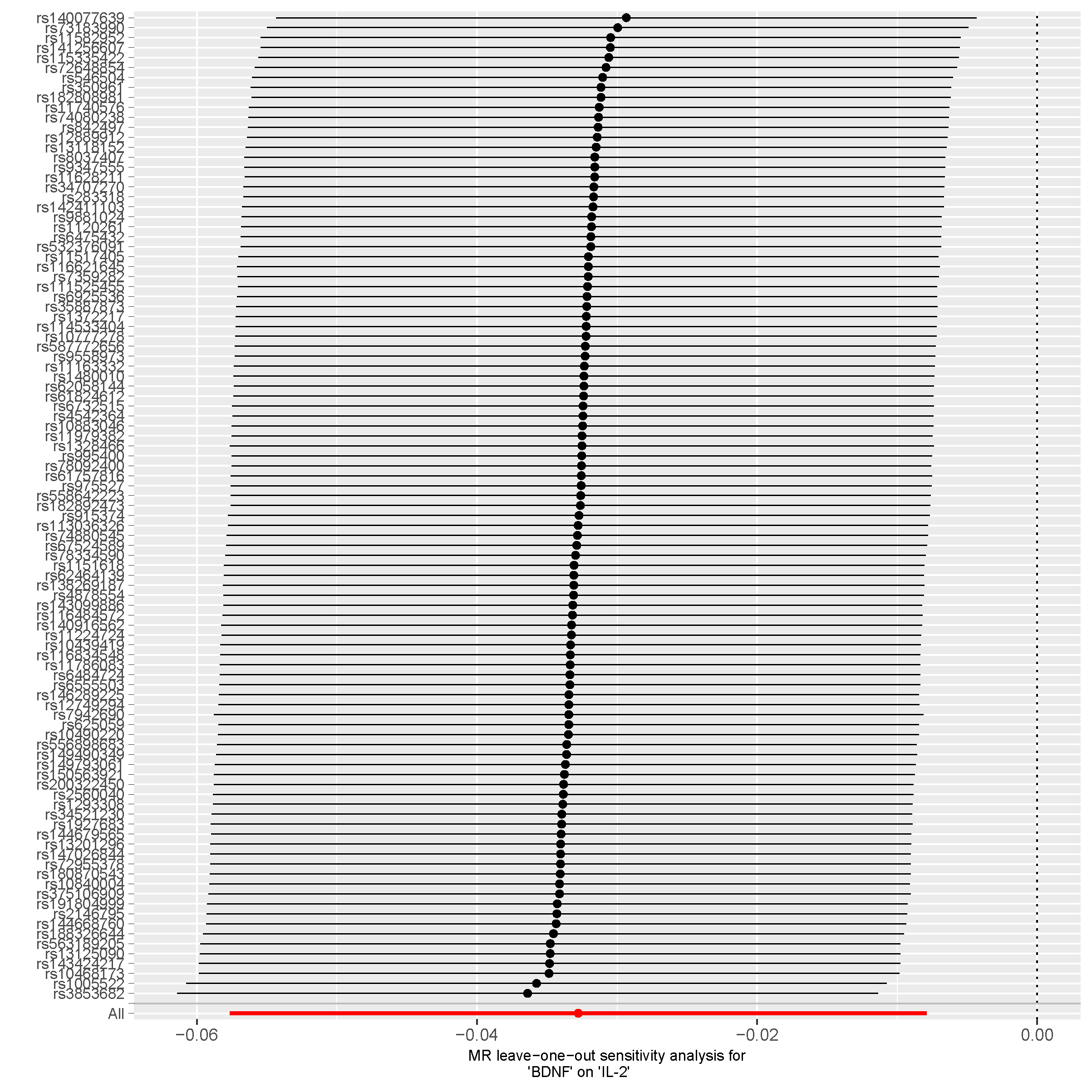


Figure S20. Leave-one-out analysis for the causal effect of BDNF on IL-20.
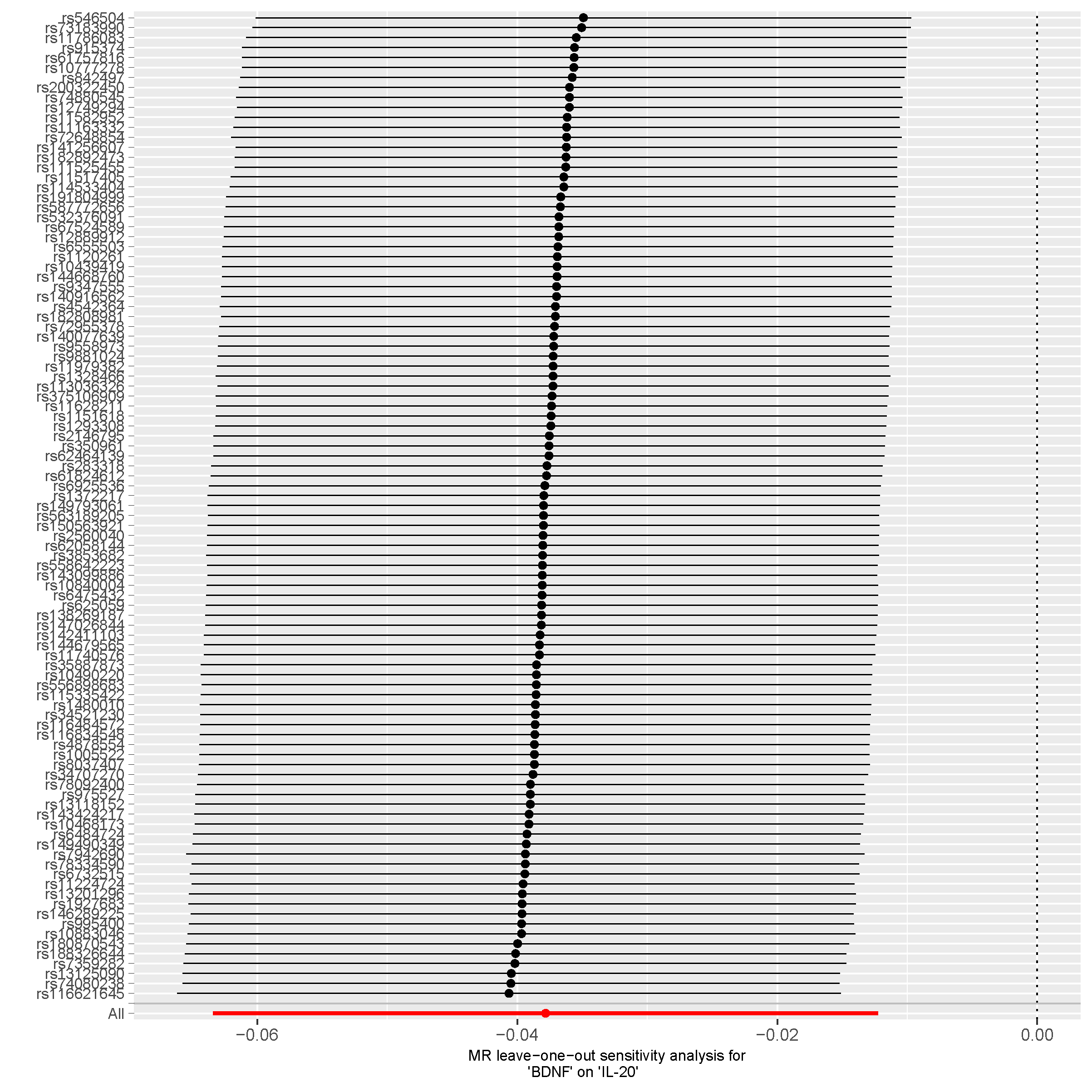


Figure S21. Leave-one-out analysis for the causal effect of BDNF on IL-20RA.
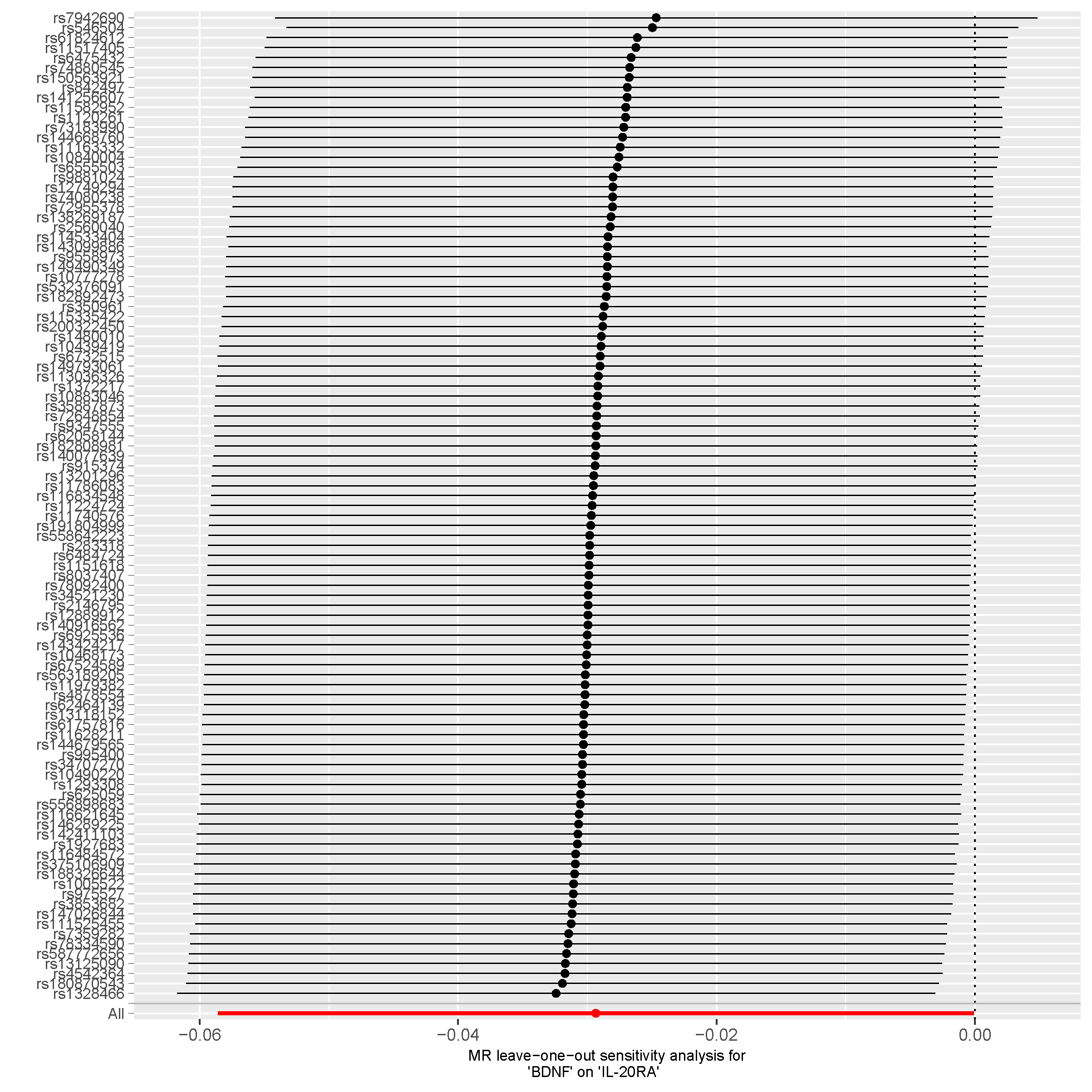


Figure S22. Leave-one-out analysis for the causal effect of BDNF on IL-24.
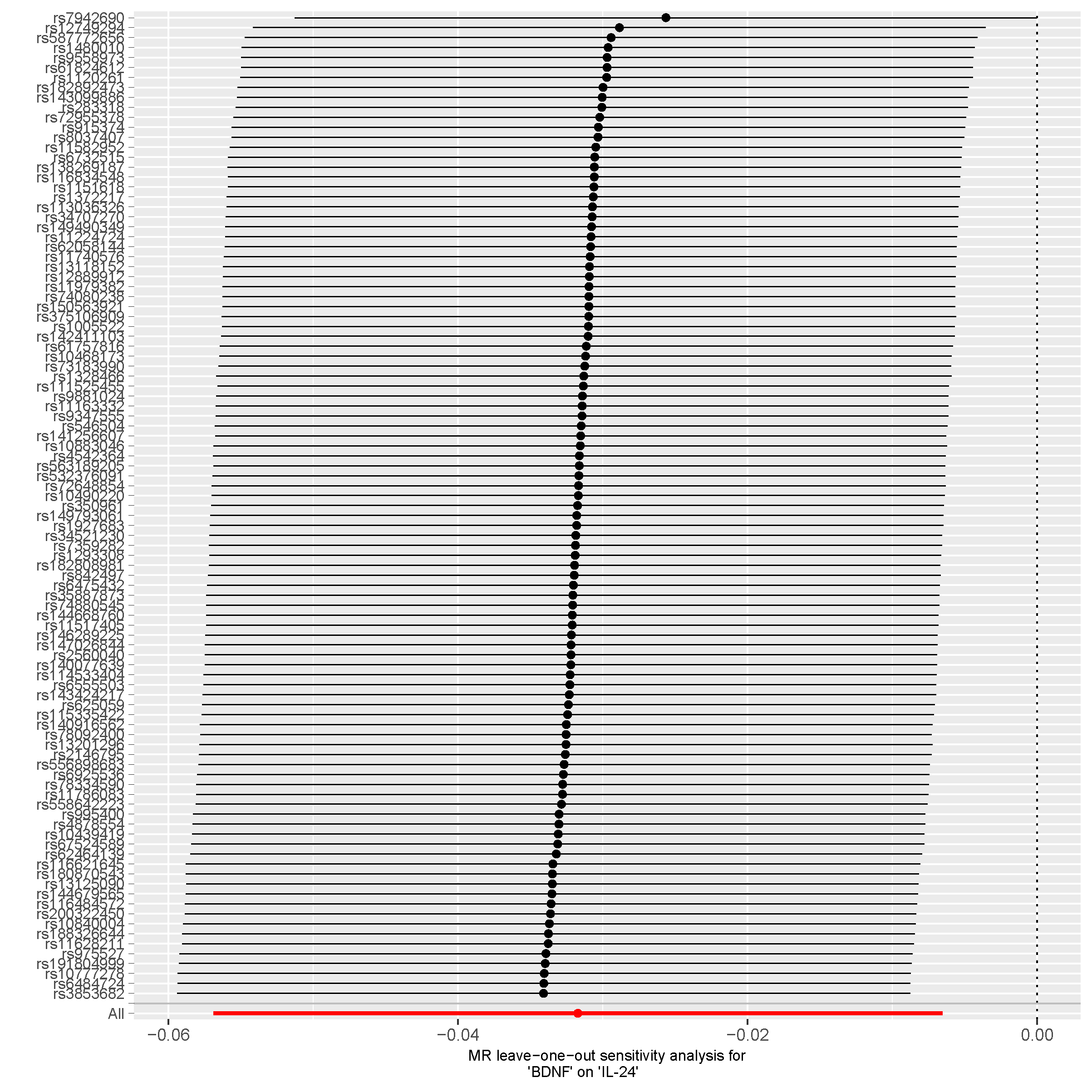


Figure S23. Leave-one-out analysis for the causal effect of BDNF on IL-33.
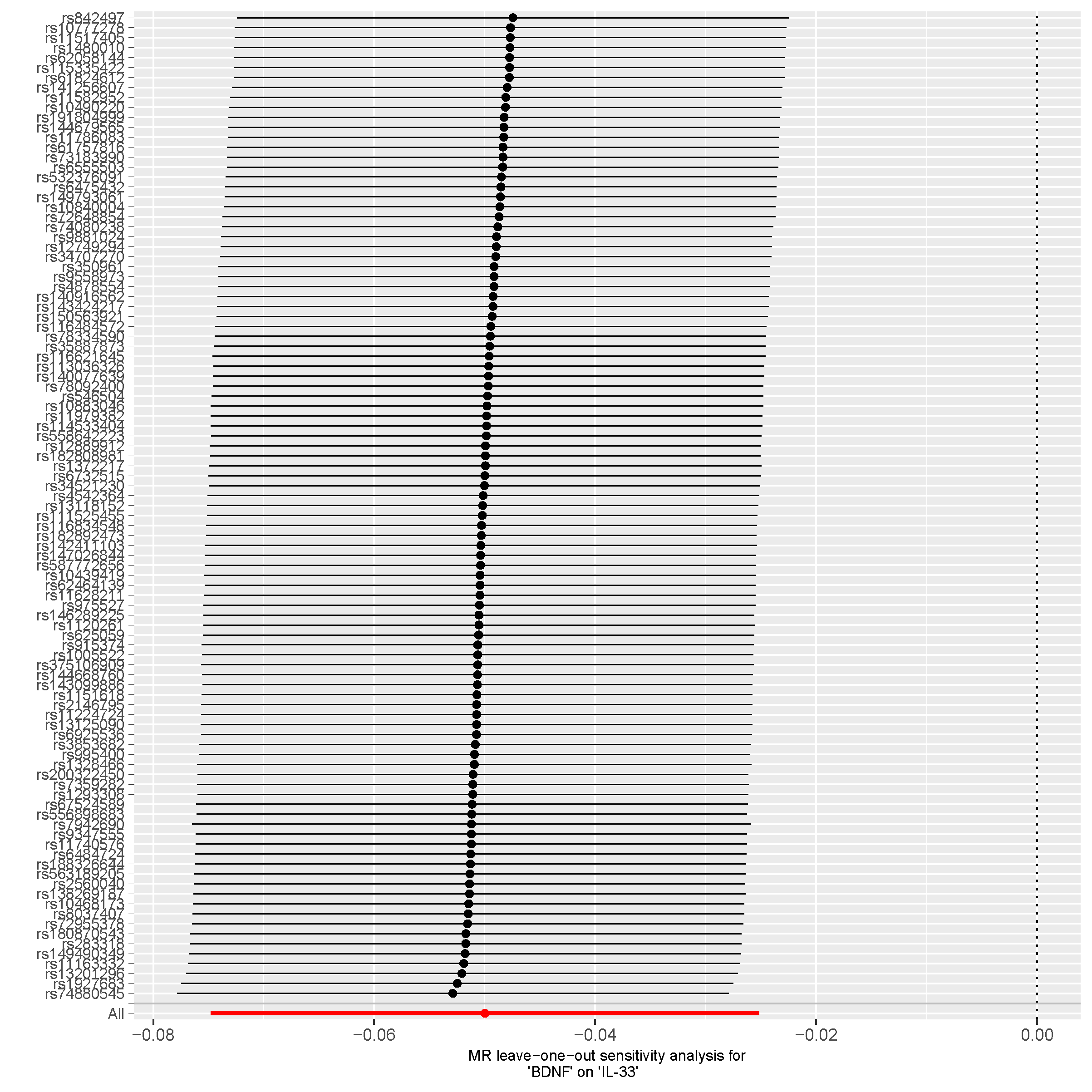


Figure S24. Leave-one-out analysis for the causal effect of BDNF on LIF.
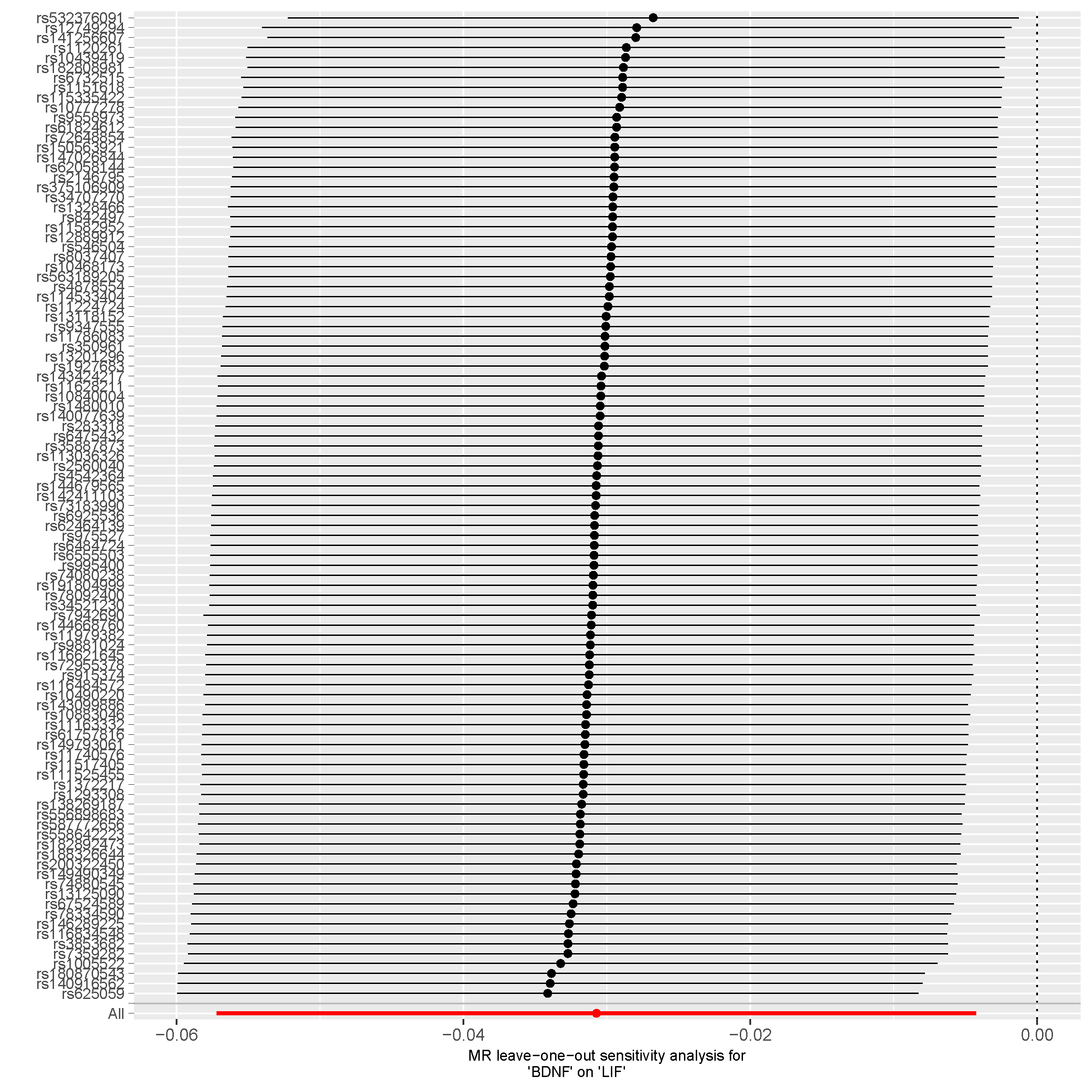


Figure S25. Leave-one-out analysis for the causal effect of BDNF on NRTN.
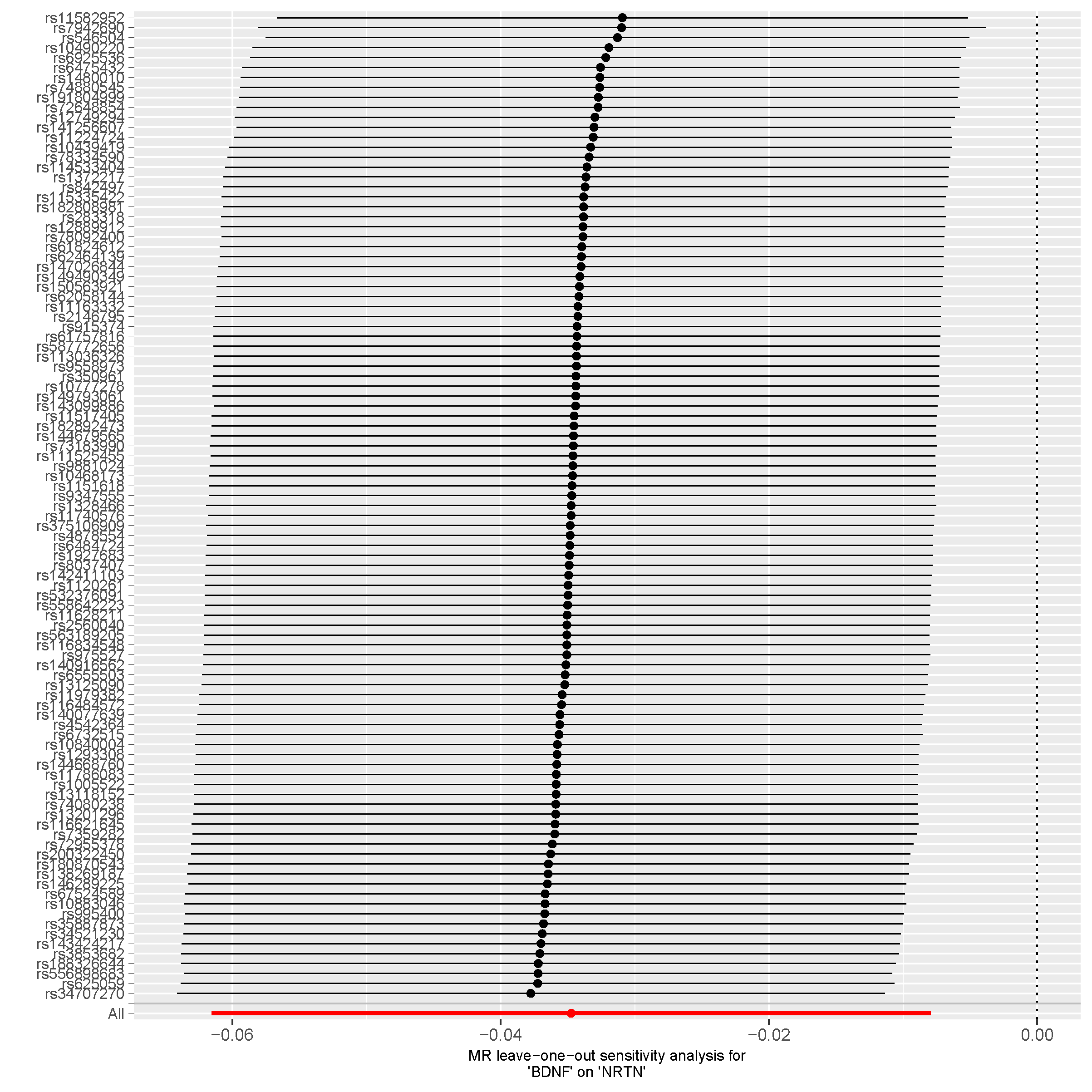


Figure S26. Leave-one-out analysis for the causal effect of BDNF on NT-3.
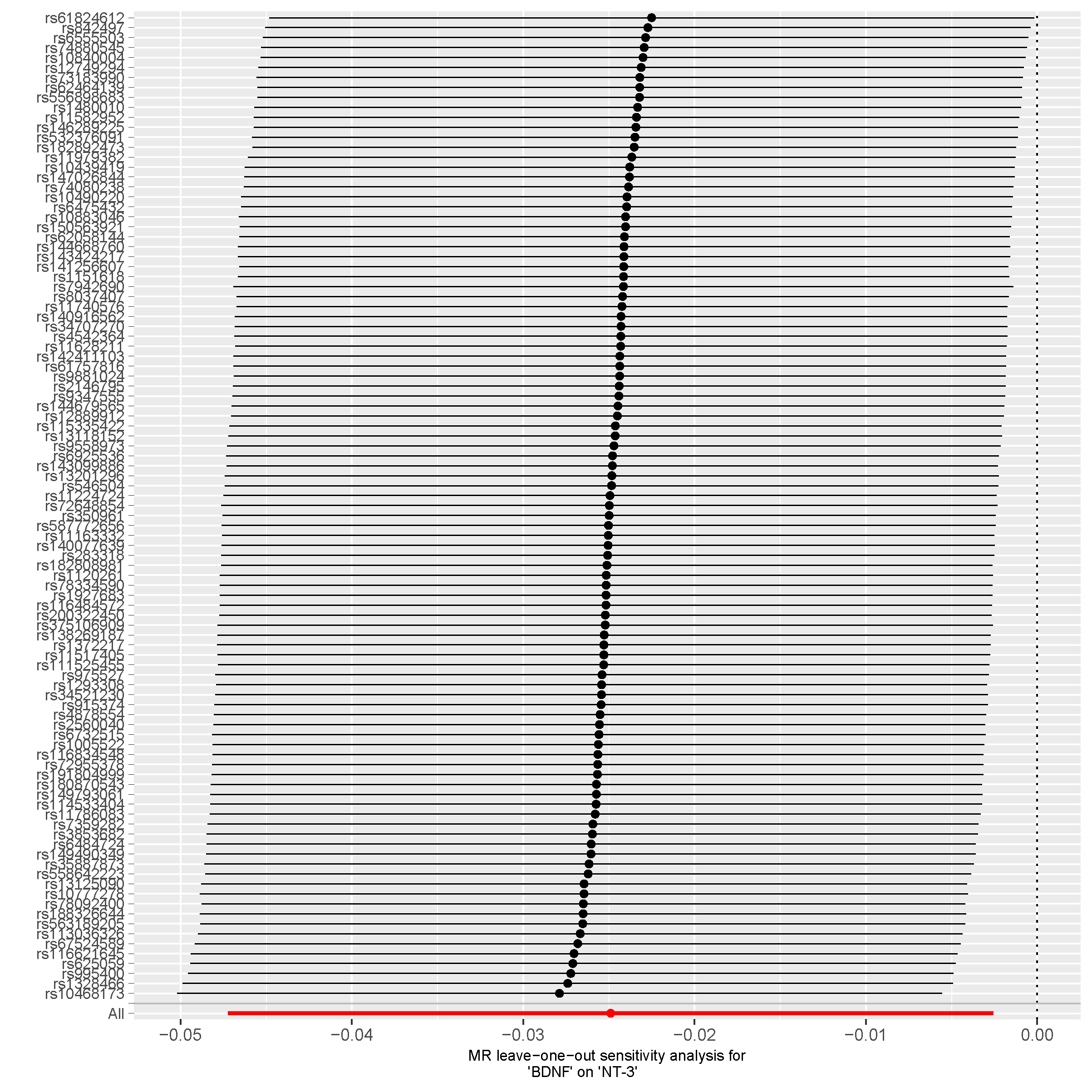


Figure S27. Forest plot to visualize the causal effect of ADA on BDNF.
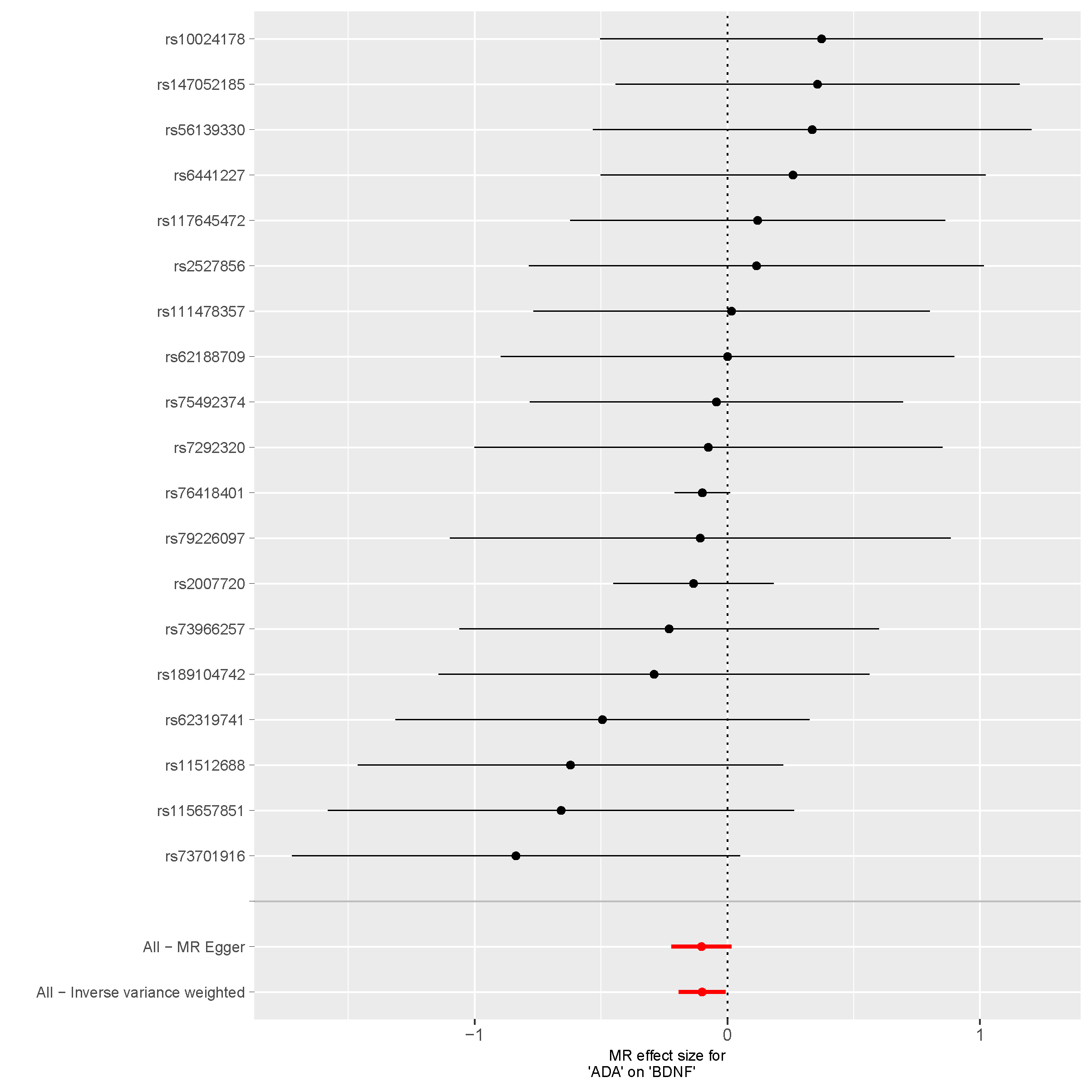


Figure S28. Forest plot to visualize the causal effect of CCL23 on BDNF.


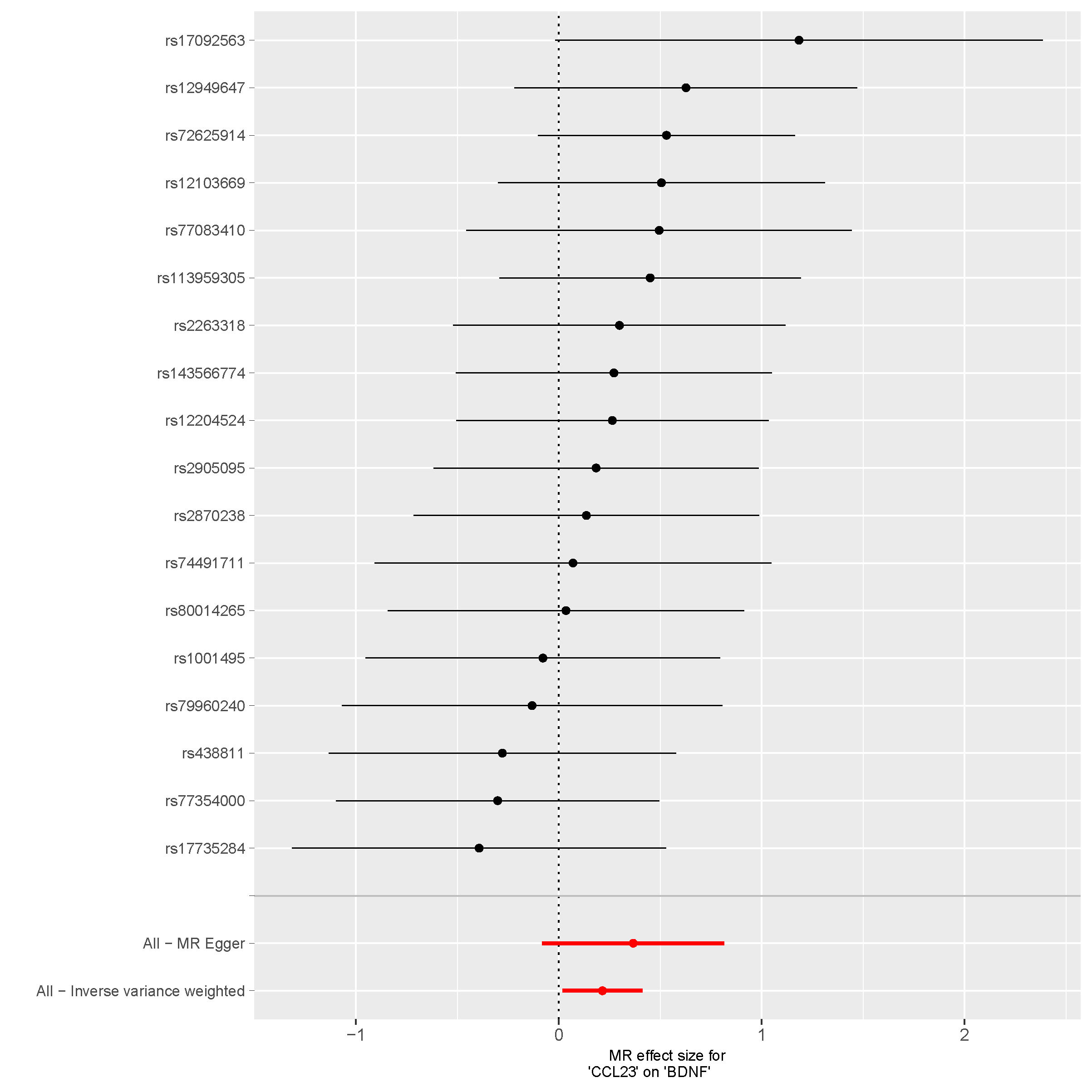


Figure S29. Forest plot to visualize the causal effect of CDCP1 on BDNF.
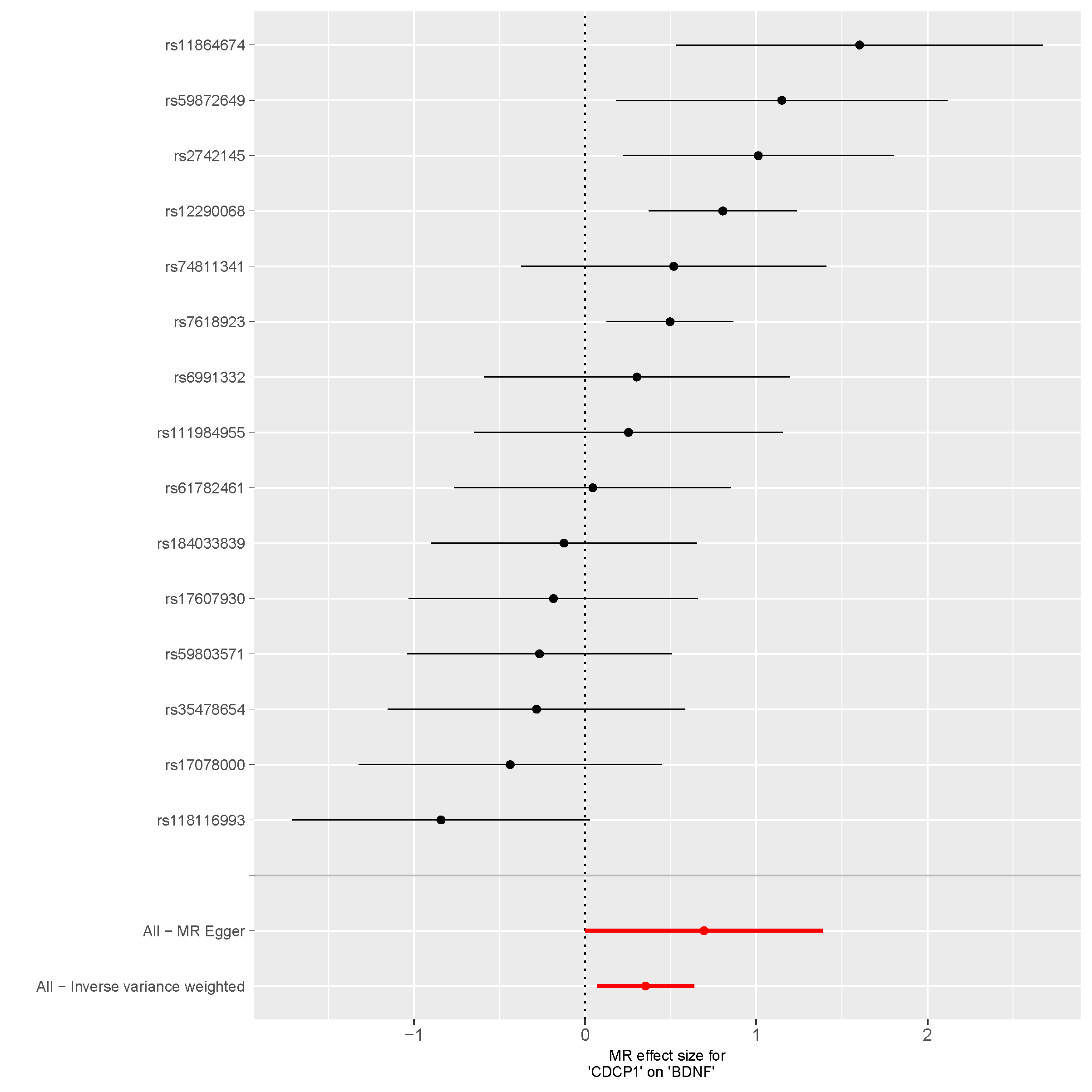


Figure S30. Forest plot to visualize the causal effect of CST5 on BDNF.
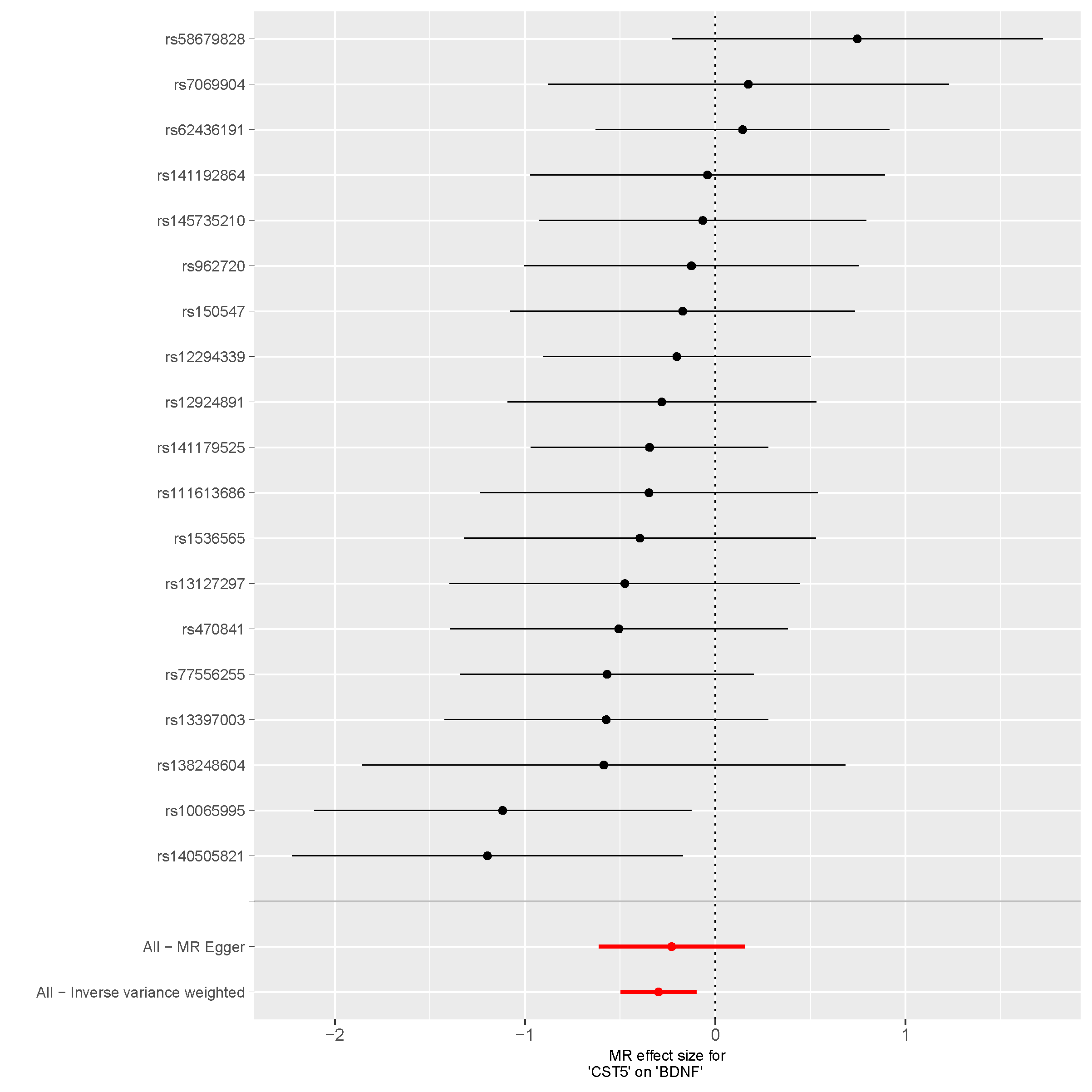


Figure S31. Forest plot to visualize the causal effect of IL-13 on BDNF.
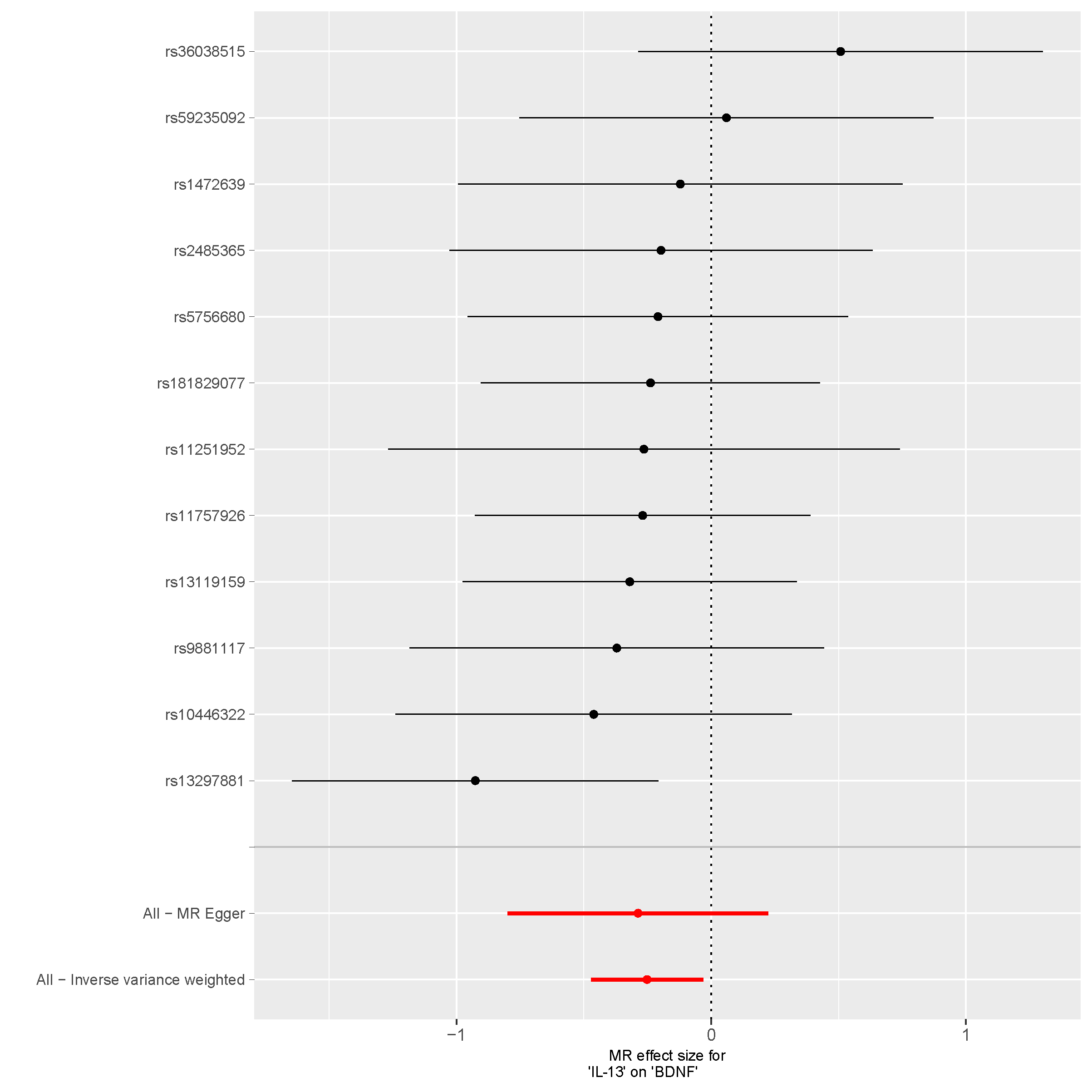


Figure S32. Forest plot to visualize the causal effect of IL-17A on BDNF.


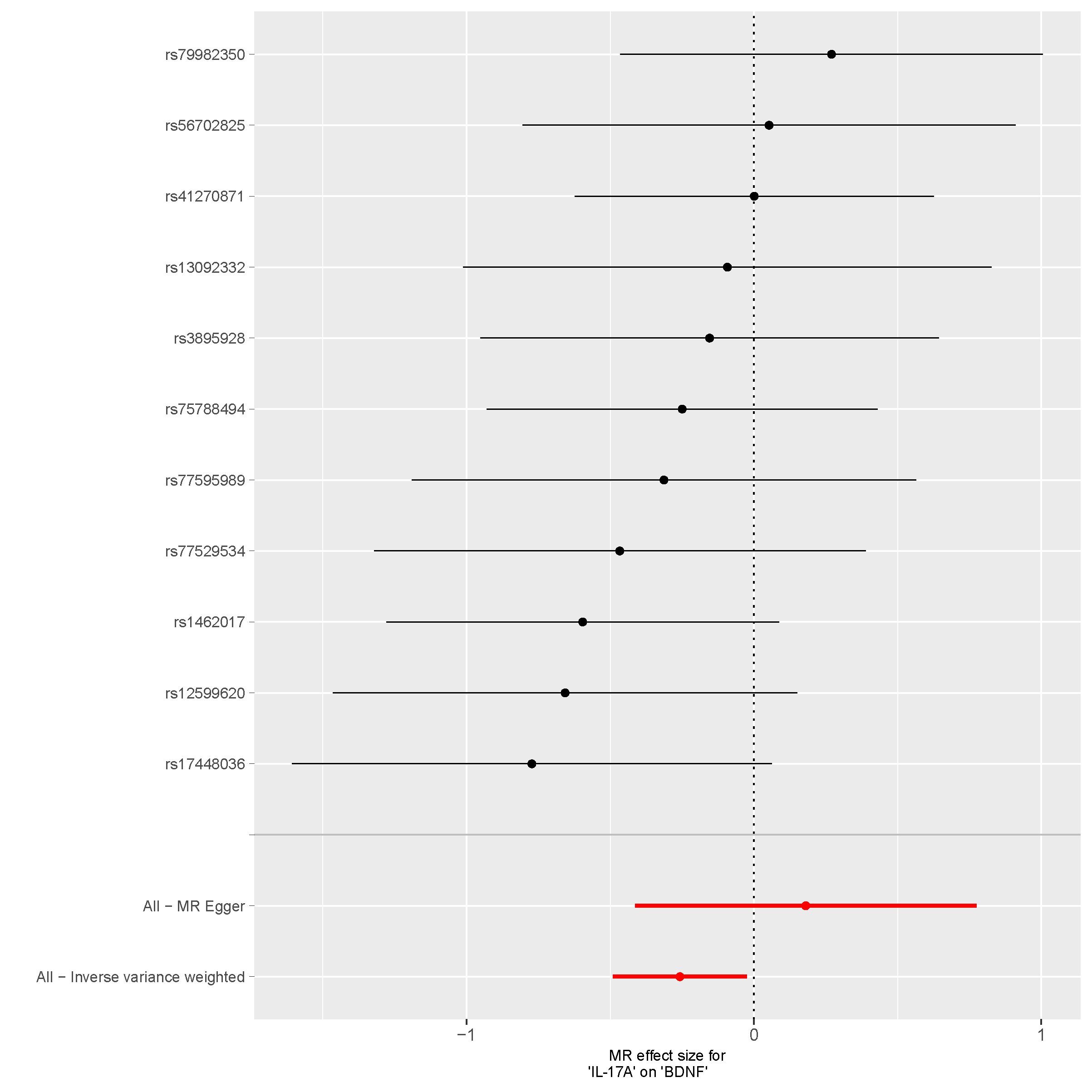


Figure S33. Forest plot to visualize the causal effect of NRTN on BDNF.
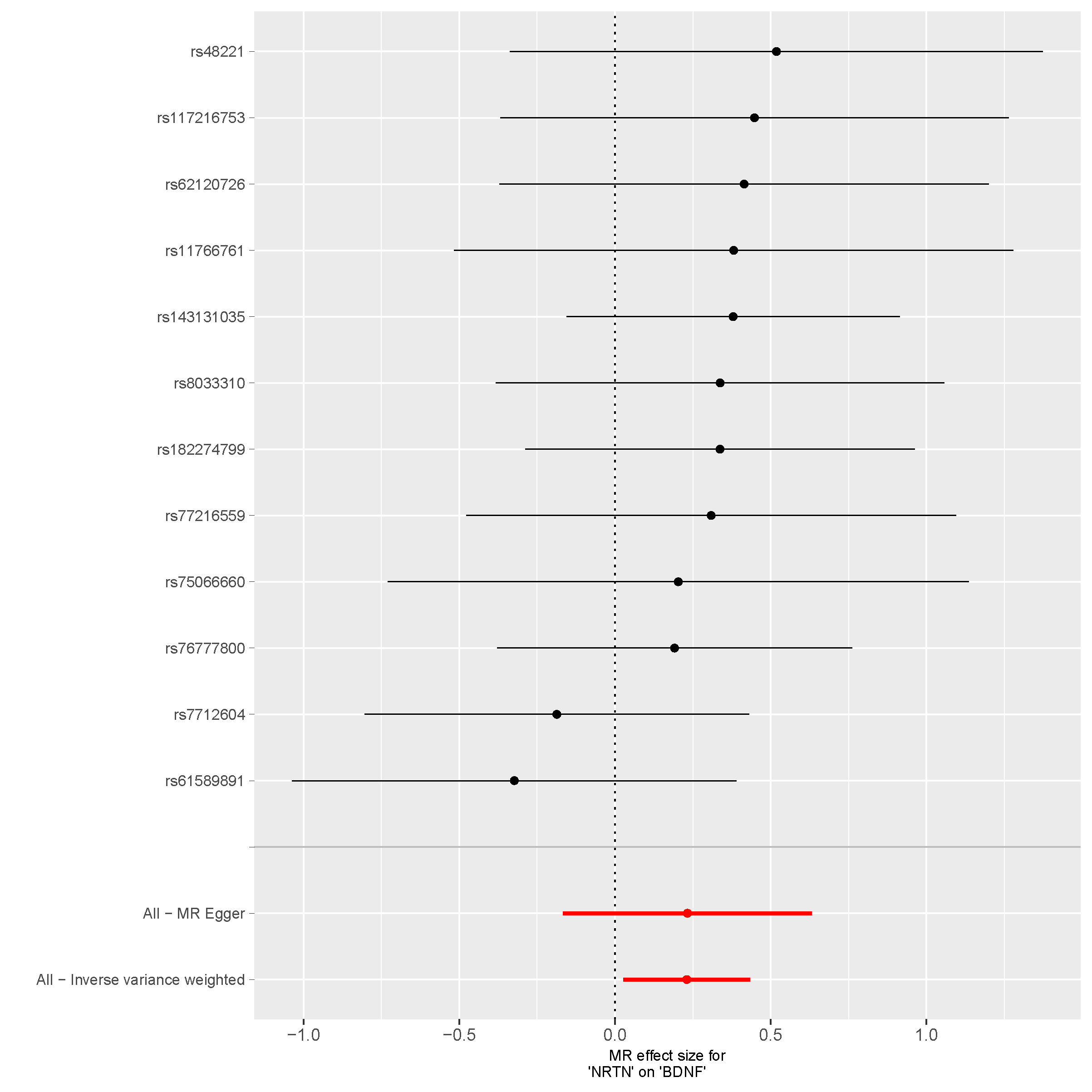


Figure S34. Forest plot to visualize the causal effect of VEGF_A on BDNF.
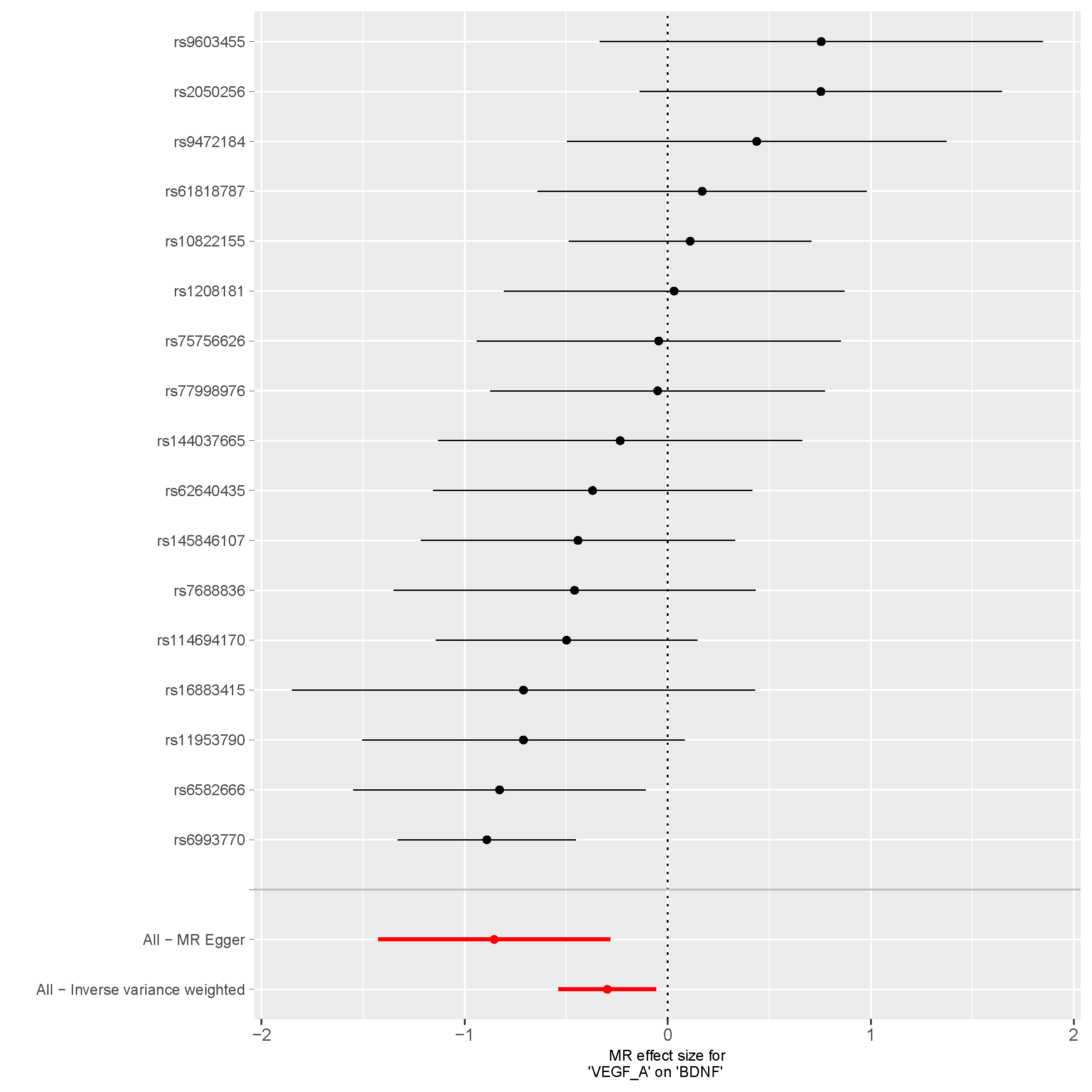


Figure S35. Leave-one-out analysis for the causal effect of ADA on BDNF.
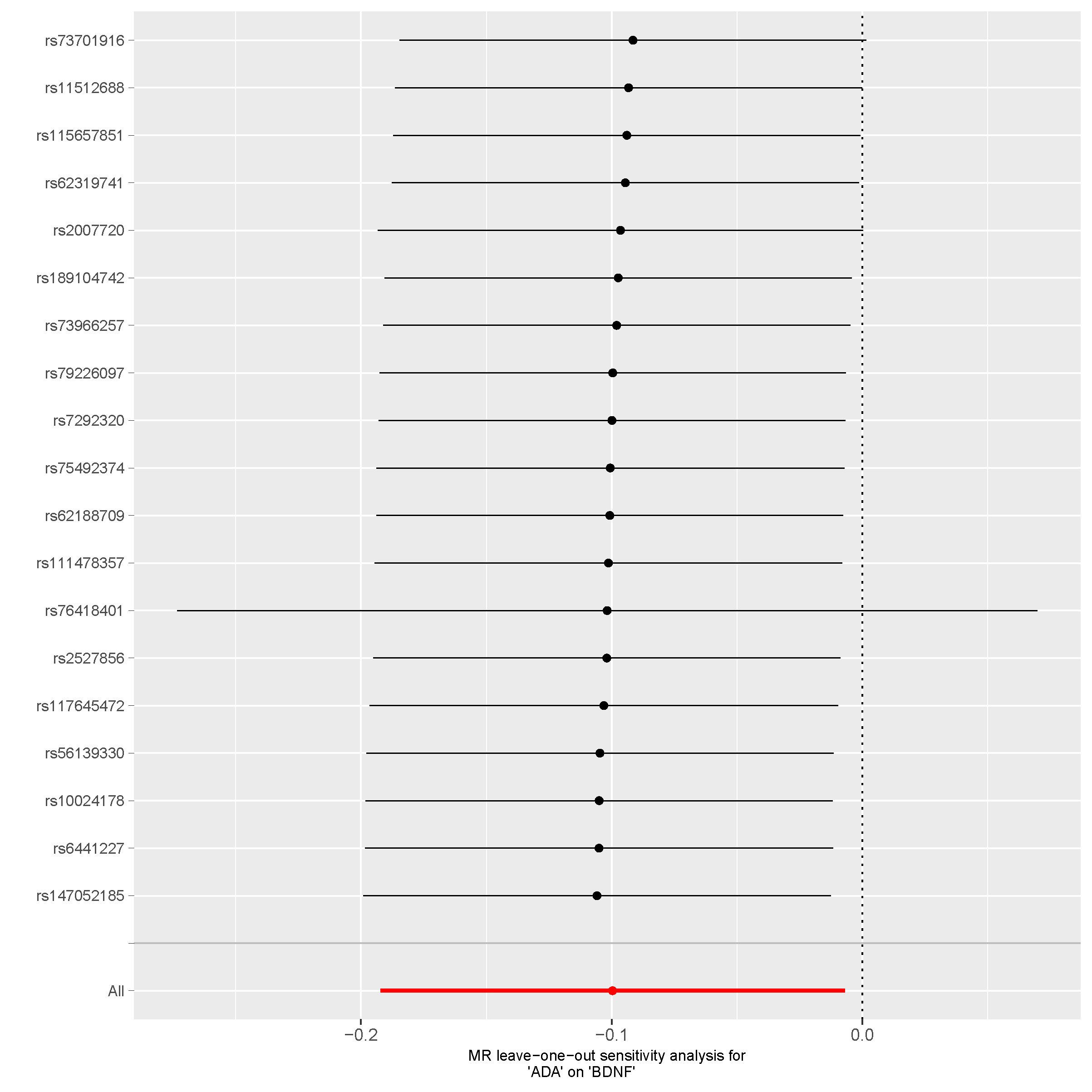


Figure S36. Leave-one-out analysis for the causal effect of CCL23 on BDNF.
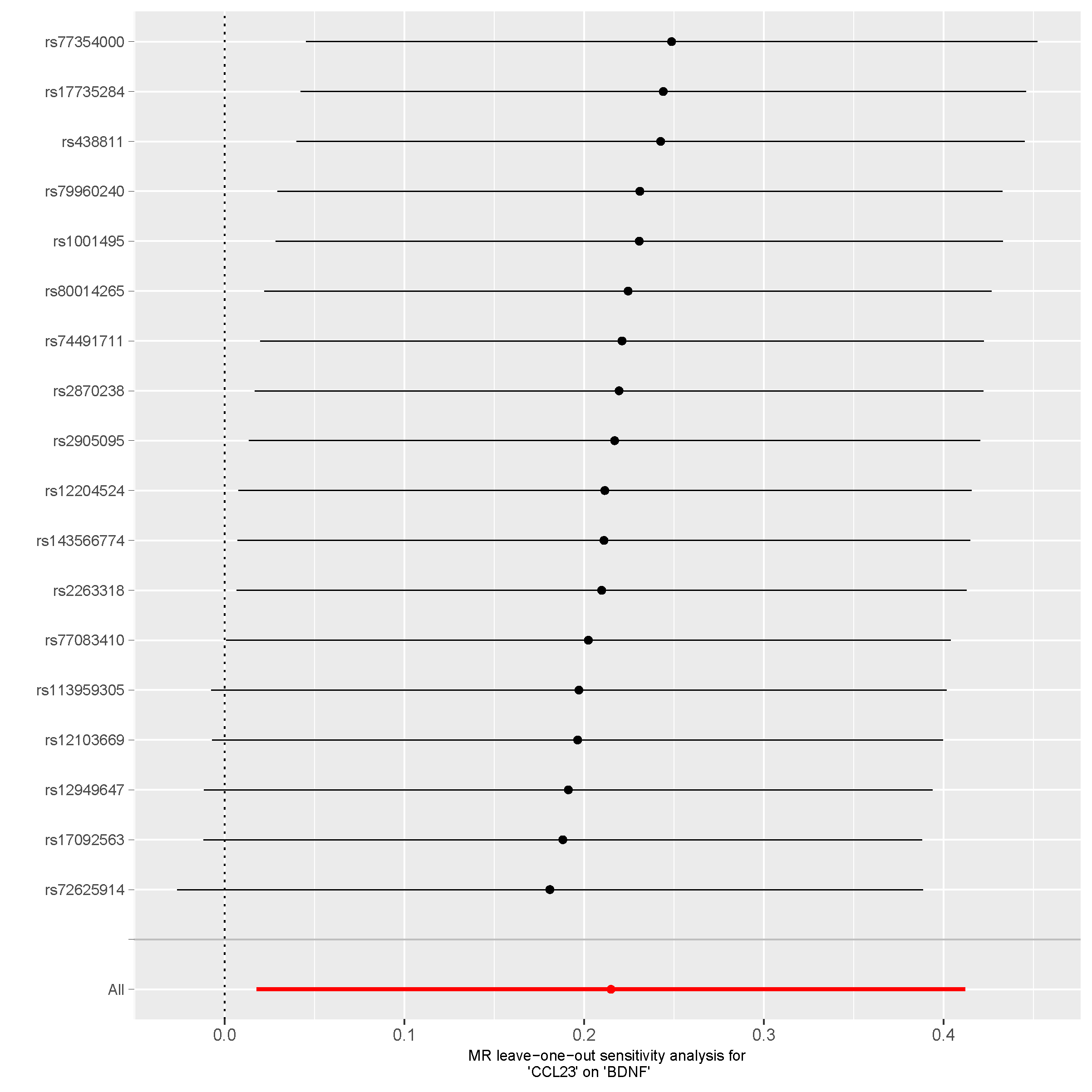


Figure S37. Leave-one-out analysis for the causal effect of CDCP1 on BDNF.
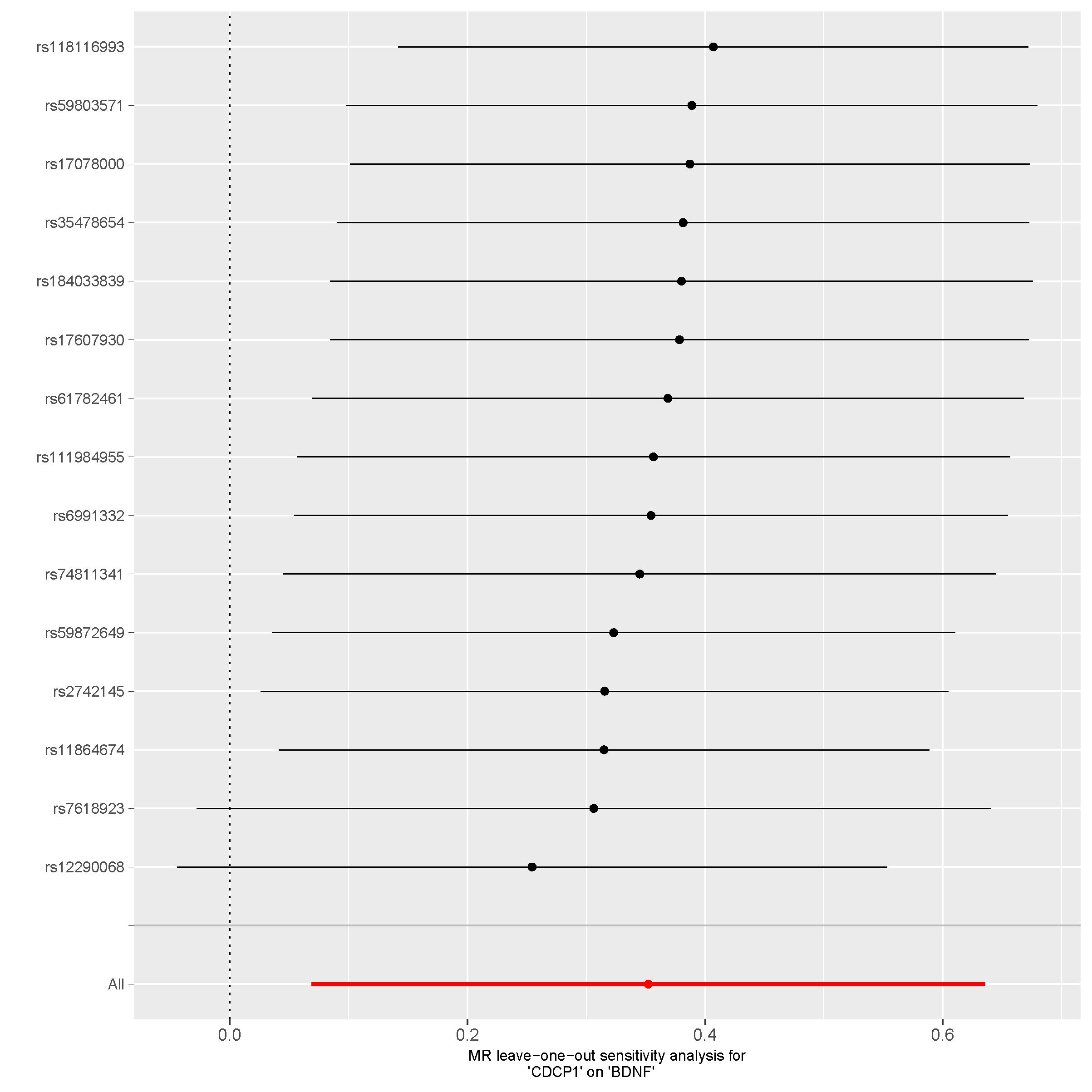


Figure S38. Leave-one-out analysis for the causal effect of CST5 on BDNF.
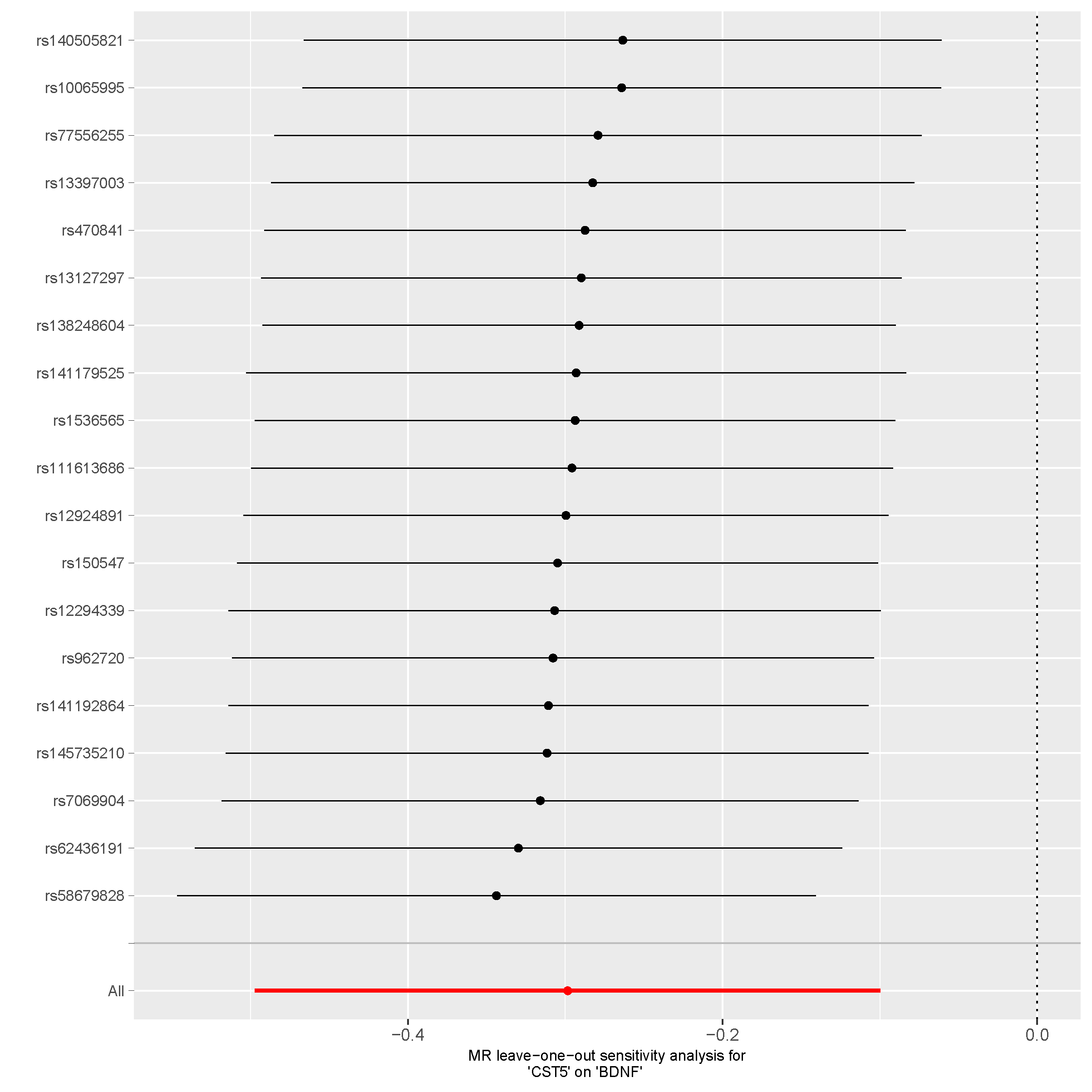


Figure S39. Leave-one-out analysis for the causal effect of IL-13 on BDNF.
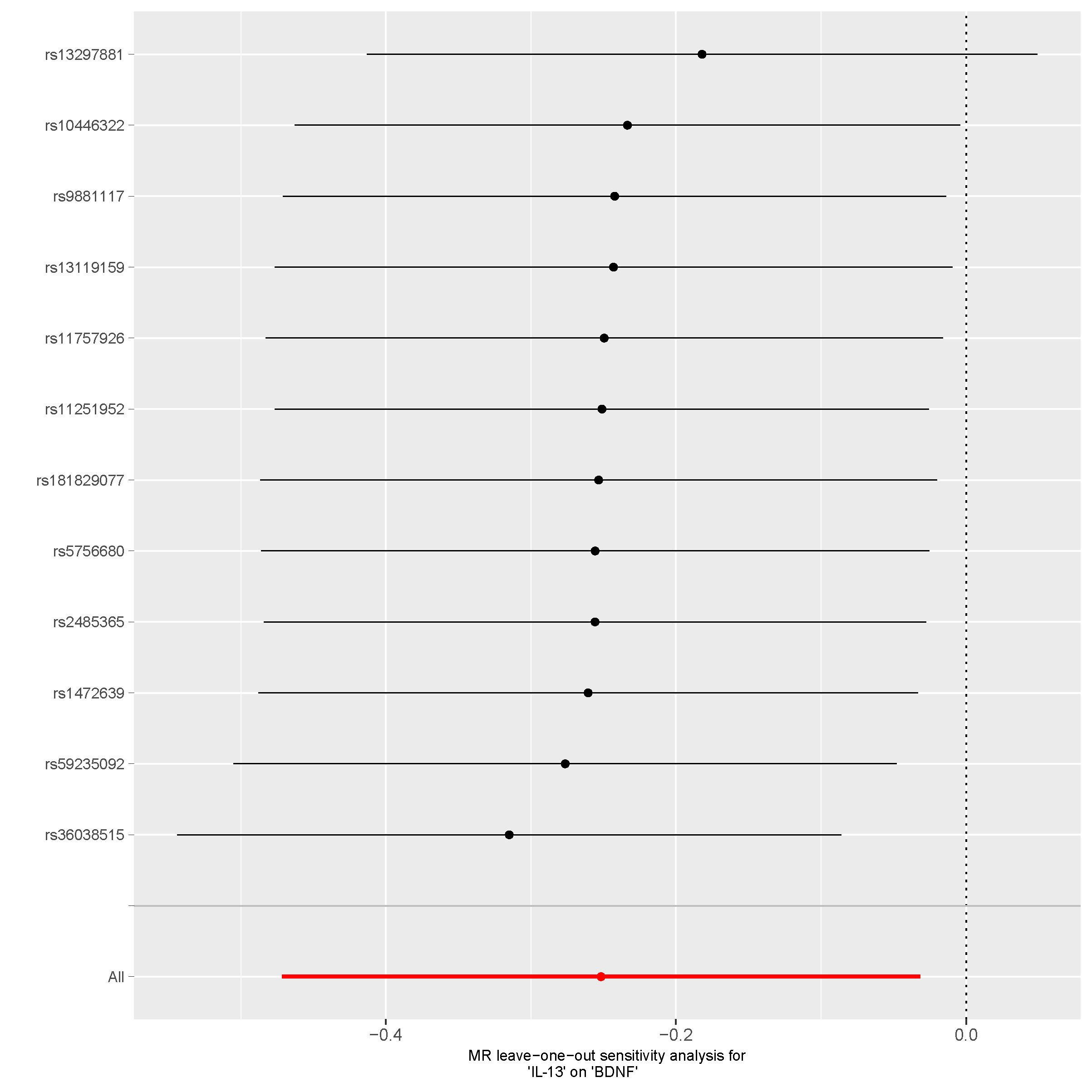


Figure S40. Leave-one-out analysis for the causal effect of IL-17A on BDNF.
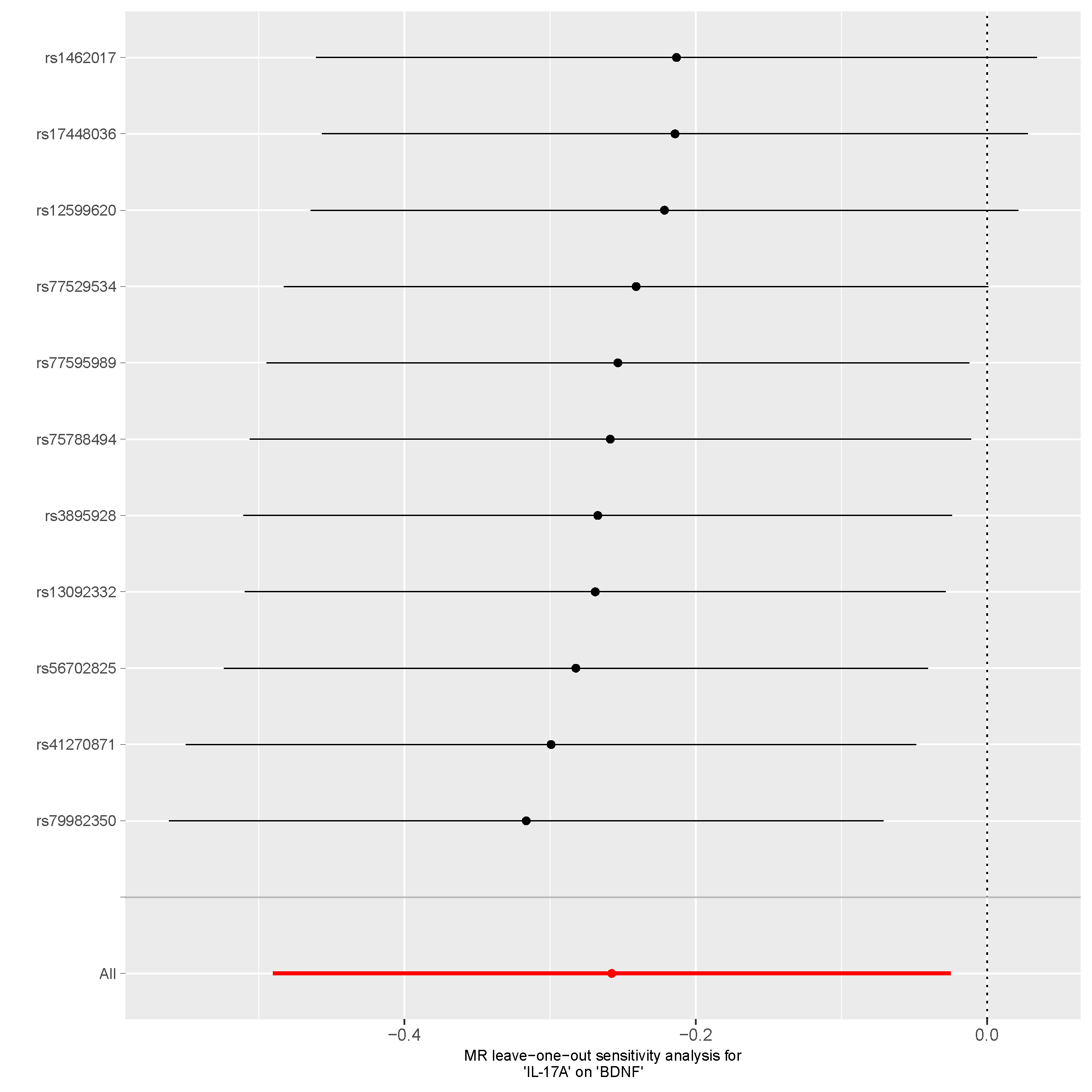


Figure S41. Leave-one-out analysis for the causal effect of NRTN on BDNF.
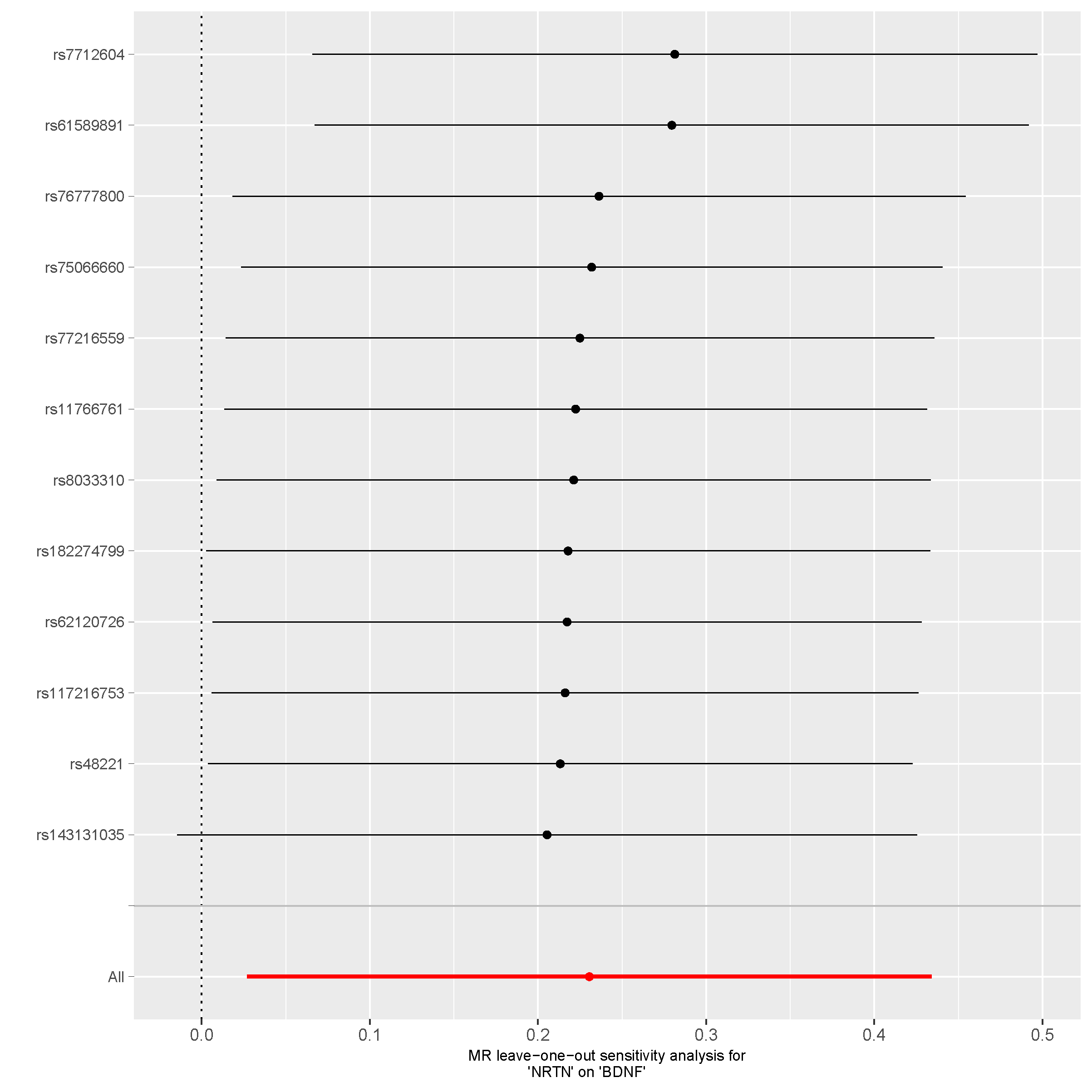


Figure S42. Leave-one-out analysis for the causal effect of VEGF_A on BDNF.
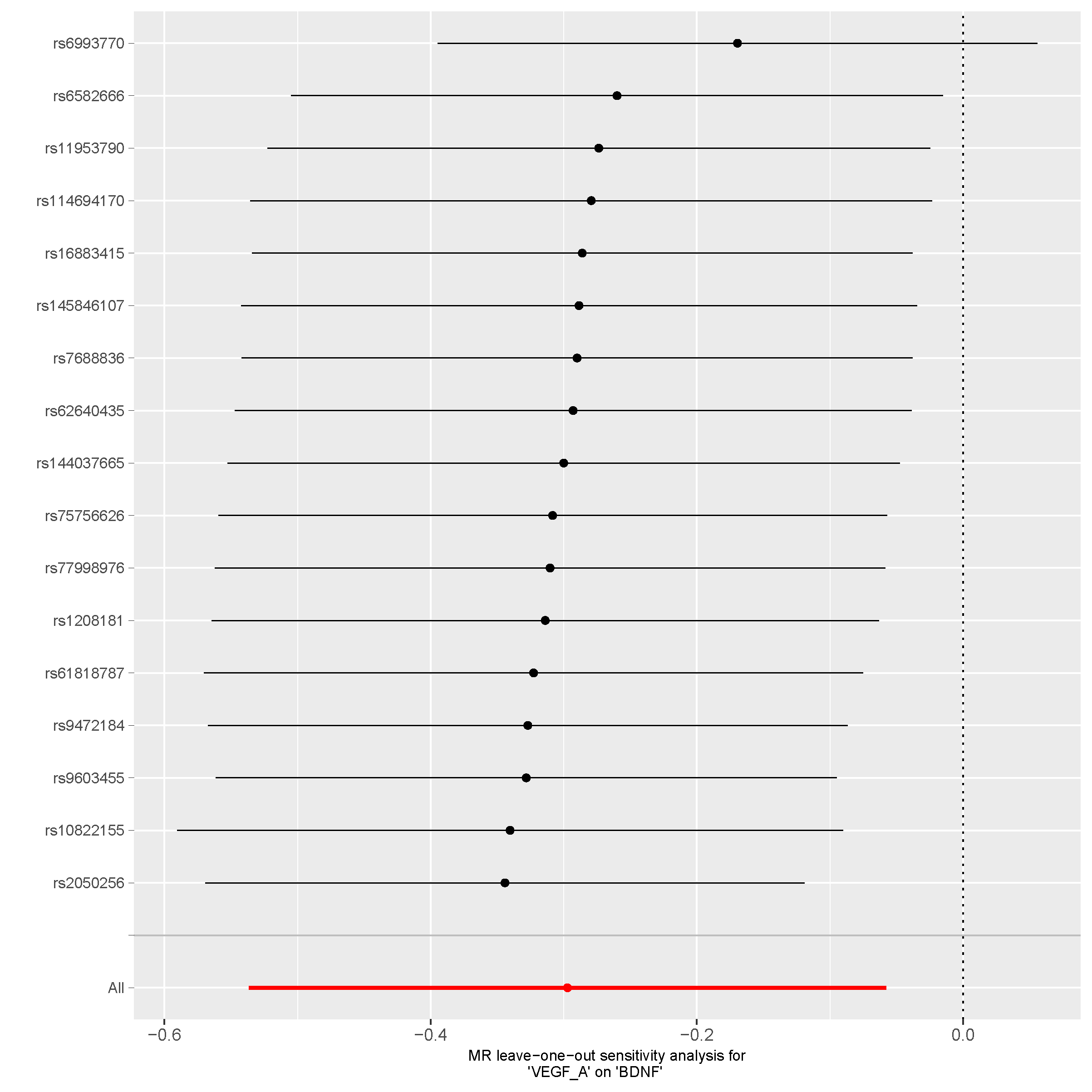

Supplement: Supplementary file 1 — Supplementary Material 1 [file 41598_2025_95546_MOESM1_ESM.doc]
